# Effectiveness of self-management interventions for long-term conditions in people experiencing socio-economic deprivation in high-income countries: a systematic review and meta-analysis

**DOI:** 10.1093/pubmed/fdad145

**Published:** 2023-08-08

**Authors:** Tosan Okpako, Abi Woodward, Kate Walters, Nathan Davies, Fiona Stevenson, Danielle Nimmons, Carolyn A Chew-Graham, Joanne Protheroe, Megan Armstrong

**Affiliations:** Research Department of Behavioural Science and Health, University College London, London WC1E 6BT, UK; Research Department of Primary Care and Population Health, University College London, London NW3 2PF, UK; Research Department of Primary Care and Population Health, University College London, London NW3 2PF, UK; Research Department of Primary Care and Population Health, University College London, London NW3 2PF, UK; Research Department of Primary Care and Population Health, University College London, London NW3 2PF, UK; Research Department of Primary Care and Population Health, University College London, London NW3 2PF, UK; Research Department of Primary Care and Population Health, University College London, London NW3 2PF, UK; School of Medicine, Keele University, Keele ST5 5BG, UK; School of Medicine, Keele University, Keele ST5 5BG, UK; Research Department of Primary Care and Population Health, University College London, London NW3 2PF, UK

**Keywords:** chronic disease, public health, systematic review

## Abstract

**Background:**

Long-term conditions (LTCs) are prevalent in socio-economically deprived populations. Self-management interventions can improve health outcomes, but socio-economically deprived groups have lower participation in them, with potentially lower effectiveness. This review explored whether self-management interventions delivered to people experiencing socio-economic deprivation improve outcomes.

**Methods:**

We searched databases up to November 2022 for randomized trials. We screened, extracted data and assessed the quality of these studies using Cochrane Risk of Bias 2 (RoB2). We narratively synthesized all studies and performed a meta-analysis on eligible articles. We assessed the certainty of evidence using GRADE for articles included in the meta-analysis.

**Results:**

The 51 studies included in this review had mixed findings. For the diabetes meta-analysis, there was a statistically significant pooled reduction in haemoglobin A1c (−0.29%). We had moderate certainty in the evidence. Thirty-eight of the study interventions had specific tailoring for socio-economically deprived populations, including adaptions for low literacy and financial incentives. Each intervention had an average of four self-management components.

**Conclusions:**

Self-management interventions for socio-economically deprived populations show promise, though more evidence is needed. Our review suggests that the number of self-management components may not be important. With the increasing emphasis on self-management, to avoid exacerbating health inequalities, interventions should include tailoring for socio-economically deprived individuals.

## Background

Long-term conditions (LTCs) are any health problem requiring active, ongoing management over at least a year, where there is no cure.^1^ LTCs affect approximately 43% of the adult population in England and are more prevalent in socio-economically deprived groups.^2^ The least affluent social class has a 60% higher prevalence of LTCs than the most affluent social class.^1^ Major socio-economic inequalities in the distribution of LTCs exist even when accounting for common risk factors such as smoking, diet and exercise.^3^

Individuals with LTCs have greater care needs than the general population.^4^ Around 70% of all health and social care funding goes to supporting people with LTCs.^1^ In England, people with LTCs account for around 70% of hospital bed days.^4^ Consequently, improving the self-management capacity of individuals has been a proposed solution to reduce the strain LTCs place on health systems.^4^ Evidence has shown that self-management approaches can improve clinical outcomes and reduce health service utilization.^5^^,^^6^

According to Barlow and colleagues, self-management can include (i) providing information about the condition, (ii) drug management, (iii) symptom management, (iv) management of psychological consequences, (v) lifestyle changes, (vi) Social support and (vii) communication with doctors.^7^ Self-management interventions aimed at the general population are less effective in people experiencing deprivation and may help maintain existing inequalities.^8^ This could be because people experiencing socio-economic deprivation are less likely to engage with the intervention.^8^ In addition, self-management involves taking a proactive approach, such as accessing preventative services, which is reduced in this population.^9^ Those experiencing deprivation have reported feeling less able to ask their doctor questions.^10^ These, along with other unexplored factors, impact the ability of interventions to effectively improve self-management in this population.

Whilst we know self-management interventions overall are less effective for people experiencing socio-economic deprivation, it has not been explored whether self-management interventions targeted specifically at this population are effective. By exploring tailored interventions, we may identify intervention active components for this population.

### Aims

This review aimed to explore whether self-management interventions targeted at people experiencing socio-economic deprivation are effective at improving outcomes. The second aim was to explore how interventions are tailored and activate components is explored.

## Methods

### Protocol registration

The protocol was registered on PROSPERO on 8 December 2021 (CRD42021289674), available from: https://www.crd.york.ac.uk/prospero/display_record.php?ID=CRD42021289674.

### Information sources and search strategy

We used previous reviews^8^^,^^11^^,^^12^ as a guiding point to develop a comprehensive list of search terms (Supplementary Material 1). The search was run in AMED, EMBASE, Medline, PsycINFO and CINAHL plus on 15 December 2021. We updated the search on 14 November 2022. The screening of titles, abstracts and full texts was undertaken independently by two authors (TO and MA). Any disagreements were resolved in collaboration with the multi-disciplinary team of authors. Data extraction and quality assessment were undertaken by TO, and all were checked by MA.

### Eligibility criteria

For studies to be eligible, the population must be adults, with a least one LTC and be experiencing socio-economic deprivation. Indicators of socio-economic deprivation considered in this review include education, income and area-level indicators (such as the index of multiple deprivation).^13^ The intervention must primarily be focused on self-management. The design must be an intervention study with a comparator population such as a randomized control trial (RCT).

The exclusion criteria were (i) the study population did not capture a dimension of socio-economic deprivation, (ii) palliative patients, (iii) no full text in English and (iv) review articles, editorials and conference proceedings. Qualitative studies were excluded from this review, but identified papers have been analysed separately to explore the barriers and facilitators of self-management in this population.^14^ Self-management of LTCs occurs within a unique socio-economic and public health context. Therefore, suitable interventions are likely to differ widely between lower and high-income countries.^15^ Whilst exploring self-management of LTCs in lower income countries is important, it requires its own discussion beyond the scope of this review. Therefore, we excluded studies set in low-income countries.

### Critical appraisal (risk of bias)

We evaluated the risk of methodological bias using version 2 of the Cochrane tool for assessing the risk of bias in randomized trials (RoB2) and version 2 for cluster-RCTs (RoB2 CRT).^16^^,^^17^ We assessed each domain using information from the trials’ main published journal articles, published protocols, clinical trial registries and supplementary appendices, when available.

### Data extraction

We created three data extraction tables on the study characteristics, intervention characteristics (modelled after the template for intervention description and replication (TiDier) guidelines^18^) and the self-management components.

### Narrative synthesis

Due to the heterogeneity of studies included in this review, the main results are presented as a narrative summary. We tabulated and compared positive study outcomes against a selection of study and intervention characteristics.

### Meta-analysis

Studies with the same outcome were screened for inclusion in a random-effects meta-analysis. Studies had to contain data on the mean change from baseline to end for both the intervention and control groups, and the standard deviations (SD). If the SDs were not reported, we converted the standard error (SE) or 95% confidence intervals.^19^ If a study did not report this data and was not available from the authors, it was excluded from the meta-analysis. To measure heterogeneity, *I*^2^ was the preferred measure because *Q*’s power is reduced when the studies are unbalanced in sample size.^20^ We assessed publication bias using a contour-enhanced funnel plot and Egger’s test. In this review, only diabetes studies were eligible.

## GRADE

The GRADE methodology was used to assess the certainty of the body of retrieved evidence from the studies included in the meta-analysis. We assessed GRADE using developed checklists^21^ and using a series of guidelines by Guyatt and colleagues, 2011.^22–26^

## Results

### Study selection

**Fig. 1:**
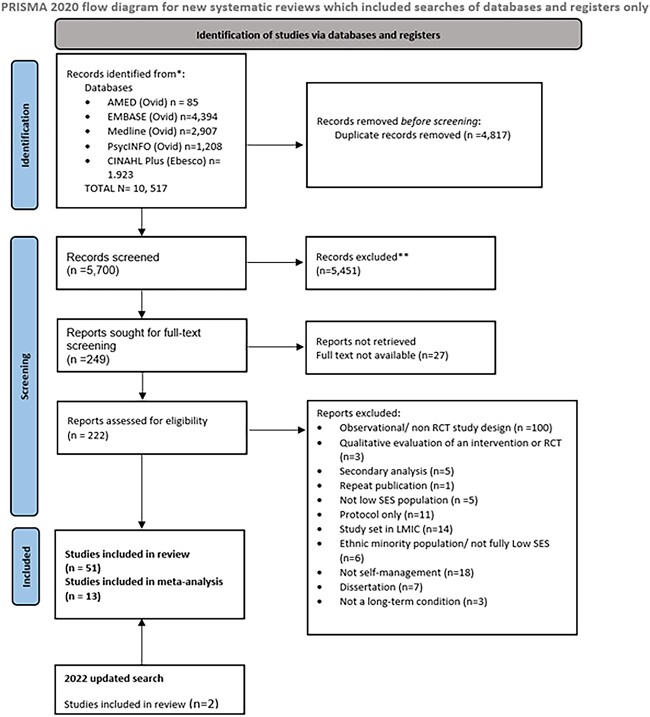
PRISMA study selection process.

After full-text screening, 49 articles met the inclusion criteria. The updated search brought this total up to 51 studies. Figure 1 summarizes the selection process.

### Study characteristics

The study characteristics are outlined in Table 1. Almost all the trials took place in the USA. The sample sizes varied from 25 to 5599 participants. Common dimensions of socio-economic deprivation in the included samples were low income or uninsured participants or the study setting was in an area of high deprivation. Most studies had predominantly African American or Hispanic/Latino study samples.

**Table 1:** Study characteristics

Ref	Author, date	Country	Follow-up time	Long term condition	Population socioeconomic status	Sample size (intervention/control)	Mean age (SD)	*n* Female (%)	Primary ethnicity (%)	Outcome	Results summary	*P* value
^27^	Anderson *et al*., 2010	USA	12 months	Type 2 diabetes	Area of deprivation: patients are at or below 200% of the federal poverty level	295 (146/149)	NP	171 (58)	African American and Hispanic/Latino	Mean HbA1c value	The difference in HbA1c between the intervention and control group was not significant	0.63
^28^	Arora *et al*., 2013	USA	6 months	Type 2 diabetes	Area of deprivation: low-income patients of a safety-net hospital	128 (64/64)	50.7 (10.2)	82 (64)	Hispanic/Latino (87)	Mean change in HbA1c (%)	The intervention group had a −0.45 (95% CI: −0.27 to 1.17) greater decrease in HbA1c levels compared to control	0.230
^29^	Baig *et al*., 2015	USA	6 months	Type 2 diabetes	Area of deprivation: low-income neighbourhood	100 (50/50)	53.7 (11.6)	81 (81)	Hispanic/Latino (97.9)	Mean change in HbA1c (%)	The intervention group had a −0.21 (CI: −0.98 to 0.55) greater decrease in HbA1c levels compared to control	>0.05
^30^	Berry *et al*., 2016	USA	15 months	Type 2 diabetes	Low income: annual household income <200% of federal poverty guidelines	80 (40/40)	51.4 (8.5)	72 (89.3)	African American (77.4)	Mean change in HbA1c (%)	Patients in the experimental group decreased their HbA1C significantly more than the control group	0.001
^31^	Chamany *et al*., 2015	USA	12 months	Type 1 and 2 diabetes	Low income	941 (443/498)	56.3 (11.7)	599 (63.7)	Hispanic/Latino (67.7)	Mean change in HbA1c (%)	The intervention group had a 0.4% greater mean decrease in HbA1c compared to the control group	0.01
^32^	Clancy *et al*., 2007	USA	12 months	Type 2 Diabetes	Inadequately insured patients	186 (96/90)	56.1	134 (72%)	African American (82.8)	Mean change in HbA1c (%)	There was no difference in HbA1c change between the groups over 12 months	NP
^33^	Davis *et al*., 2010	USA	12 months	Type 2 diabetes	Area of deprivation: rural, medically underserved and low income	165 (85/80)	59.9 (9.4)/59.2 (9.3)^*^^*^	123 (75%)	African American (75.3/72.5)	Mean change in HbA1c (%)	The improvement in HbA1c was greater in the intervention group compared with usual care	0.004
^34^	Fitzpatrick *et al*., 2022	USA	6 months	Type 2 diabetes	At least one of the four social risks (food insecurity, unstable housing, difficulty paying for medical care and lack of transportation)	110 (56/54)	53.3 (12)	77 (70)	Multi-ethnic	Mean change in HbA1c (%)	Within each group, there was a clinically significant reduction in HbA1c. −0.72% in the intervention group and −0.54% in the control	Between-group difference not reported
^35^	Fortmann al., 2017	USA	6 months	Type 2 diabetes	Low income, uninsured and low educational attainment	126 (63/63)	48.43 (9.80)	94 (75%)	Hispanic/Latino (100)	Mean change in HbA1c (%)	There was a significant time-by-group interaction effect for HbA1c, indicating that over time, the intervention group had greater glycaemic control compared to the control group	0.03
^36^	Frosch *et al*., 2011	USA	6 months	Type 2 diabetes	Low income and underinsured	201 (100/101)	56.7 (8.3)/54.3 (8.9)	97 (48.3%)	Hispanic/Latino (55.80)	Mean change in HbA1c (%)	There was an overall decrease in HbA1c values for both groups. However, there was no significant interaction effect of group by time	0.49
^37^	Gary *et al*., 2009	USA	24 months	Type 2 diabetes	‘Socioeconomically disadvantaged’	488 (235/253)	58 (11)	358 (73)	African American (100)	Mean change in HbA1c (%)	There were no within-group or between-group differences in HbA1c change	0.44
^38^	Greenhalgh *et al*., 2011	UK	6 months	Type 2 diabetes	Socio-economically deprived area	157 (79/78)	58 (12)	110 (70)	Multi-ethnic	Mean change in HbA1c (%)^*^	There was no significant difference in the within-group change in HbA1c between the intervention and control	0.364
^39^	Hill-briggs *et al*., 2011	USA	9 months	Type 2 diabetes	Low income	56 (29/27)	61.3 (10.9)	33 (58.9)	African American (100)	Mean change in HbA1c (%)	The intervention group had a larger reduction in HbA1C change	0.02
^40^	Lynch *et al*., 2014	USA	6 months	Type 2 diabetes	Low income	61 (30/31)	54.1 (10.0)	41 (67.2)	African American (100)	Mean change in HbA1c (%)^*^	There was no significant difference in HbA1c reduction between the groups	0.10
^41^	Lynch *et al*., 2018	USA	12 months	Type 2 diabetes	Low income	211 (106/105)	55.0 (10.3)	148 (70.1)	African American (100)	Mean change in HbA1c (%)	While the HbA1c change was greater in the intervention group than the comparison group, the difference was not statistically significant	0.52
^42^	Nelson *et al*., 2017	USA	12 months	Type 2 diabetes	Low income: household income of less than 250% of the federal poverty level	287 (145/142)	52.5 (9.3)	140 (48.8)	Multi-ethnic	Mean change in HbA1c (%)	There was no significant difference in the mean HbA1c change in the intervention group compared to the control group	0.54
^43^	Pérez-Escamilla *et al*., 2015	USA	12 months	Type 2 diabetes	Low income	211 (105/106)	56.3 (11.8)	155 (73.5)	Latino/Hispanic (100)	Mean change in HbA1c (%)	The intervention led to a greater reduction in HbA1c, compared to the control	0.021
^44^	Philis-Tsimikas *et al*., 2011	USA	4 months	Type 2 diabetes	Underinsured, low income	207(104/103)	52.2 (9.6)/49.2 (11.8)	146 (70.5)	Mexican American (100)	Mean change in HbA1c (%)	The intervention group had a significant decrease in HbA1c, from baseline to month 4 (−1.7%, *P* = 0.001). The control group had a non-significant reduction of −1.1% (*P* = 0.14)	Between-group difference not reported
^45^	Protheroe *et al*., 2016	UK	7 months	Type 2 diabetes	Residents from an area of deprivation	76 (39/37)	64.7 (11.2)/61.5 (10.1)	38 (50)	NP	Mean change in HbA1c values	No difference in HbA1c change between the groups	0.183
^46^	Pyatak *et al*., 2018	USA	6 months	Type 1 and 2 diabetes	Low income/education—self-reported household income was below 250% of the federal poverty level or neither parent had a bachelor’s degree	81 (41/40)	22.6 (3.5)	51 (63)	Hispanic/Latino (78)	Mean change in HbA1c (%)	The intervention group had greater improvement in HbA1c compared to the control group	0.01
^47^	Rosal *et al*., 2005	USA	6 months	Type 2 diabetes	Low-income	25 (15/10)	62.6 (8.6)	20 (80)	Hispanic/Latino (100)	Mean change in HbA1c (%)	The HbA1c decrease was larger in the intervention group compared to control	0.005
^48^	Rosal *et al*., 2011	USA	12 months	Type 2 diabetes	Low income	252 (124/128)	NP	93 (76.6)	Hispanic/Latino—Puerto Rico (87.7)	Mean change in HbA1c (%)	The intervention effect was not significant	>0.293
^49^	Ruggiero *et al*., 2014	USA	12 months	Type 2 diabetes	Low income	266 (134/132)	53.15 (12.36)	183 (68.8)	African American (52.6) and Hispanic/Latino (47.4)	Mean change in HbA1c (%)	No intervention effect was found, and no differences were found for A1C	NP
^50^	Schillinger *et al*., 2009	USA	12 months	Type 2 diabetes	Low income and underinsured	339 (113/112/114)	56.1 (12.0)	200 (59.0)	Multi-ethnic	Patient assessment of chronic illness care (PACIC)	Both the intervention groups showed a greater improvement in PACIC compared to control	For ATSM: *P* < 0.0001 For GMV: *P* = 0.04
^51^	Schoenberg *et al*., 2017	USA	7 months	Type 2 diabetes	Area of poverty	41 (20/21)	58.24 (10.77)	30 (65.85)	Anglo-white (100)	Mean change in HbA1c (%)	There was no overall difference in HbA1c change over time	0.22
^52^	Seligman *et al*., 2018	USA	6 months	Type 2 diabetes	Food insecure—food bank recipients	568 (285/283)	54.8 (11.4)	384 (68.3)	Hispanic/Latino (52.1)	Risk difference in mean HbA1c (%) at follow-up	No evidence of a difference in HbA1c at follow-up	0.16
^53^	Shea *et al*., 2006	USA	12 months	Type 2 diabetes	Medicare beneficiaries	1665 (844/821)	70.82 (6.63)	1040 (62.82)	Multi-ethnic	Mean change in HbA1c (%)	The intervention group had a greater reduction in mean HbA1c level compared to control	0.006
^54^	Sixta and Ostwald, 2008	USA	6 months	Type 2 diabetes	Low income	131 (63/68)	56.3	93 (71)	Mexican American	Mean change in HbA1c (%)^*^	There was no difference in the HbA1c level over the study period, within both the intervention and control group	NP
^55^	Skelly *et al*., 2009	USA	9 months	Type 2 diabetes	Rural, low income	180 (60/60/60)	67	180 (100)	African American (100)	Mean change in HbA1c (%)	There were no differences in the amount of decline between the 3 study arms	NP
^56^	Spencer *et al*., 2018	USA	18 months	Type 2 diabetes	Area of deprivation residents	222(60/89/73)	48.9 (10.6)	135 (60.8)	Latino	Mean change in HbA1c (%)	From 6 to 12 months, improvements in HbA1c were sustained for participants randomized to the enhanced intervention group (*n* = 60) (−0.63% [95% CIs: −1.06 to −0.19]; *P* < 0.01) but not the regular intervention or the control groups	NP
^57^	Talavera *et al*., 2021	USA	6 months	Type 2 diabetes	Low education and low income	456 (225/231)	55.72 (9.82)	290 (63.7)	Hispanic/Latino (96.5)	Mean change in HbA1c (%)	The group × time interaction effect (−0.32, 95% CI: −0.49 to −0.15) indicated greater improvement in HbA1c level over 6 months in the intervention group compared to control	<0.01
^58^	Thom *et al*., 2013	USA	6 months	Type 2 diabetes	Low income	299 (148/151)	55	156 (52.2)	Multi-ethnic	Mean change in HbA1c (%)	Patients in the intervention group had a 0.77% greater decrease in HbA1c levels at 6 months compared to control	0.01
^59^	Wang *et al*., 2018	USA	6 months	Type 2 diabetes	Low income, underinsured and uninsured	26 (11/9/6)	56.4	16 (62)	African American/Black (65.38)	Mean change in HbA1c (%)	At 6 months, there were no statistically significant group differences in HbA1c level change	0.44
^60^	Wayne *et al*., 2015	Canada	6 months	Type 2 diabetes	Low income	97 (48/49)	53.2 (11.3)	70 (72)	Black-Caribbean (40)	Mean change in HbA1c (%)	There was no between-group differences in mean HbA1c change from baseline to 6 months	0.48
^61^	Whittemore-2020	Mexico/USA	6 months	Type 2 diabetes	Low income	47 (26/21)	55.35 (8.75)	31 (68)	Hispanic/Latino	Mean change in HbA1c (%)	There was little difference of changes between the groups	0.11
^62^	Aikens *et al*., 2022	USA	12 months	Depression	Low-income	204 (108/96)	48.6 (12.2)	165 (80.8)	Caucasian (74.1)	Depressive symptom severity (Patient Health Questionnaire 9)	The intervention group’s mean PHQ-9 total had a greater reduction compared to the control	0.004
^63^	Apter *et al*., 2019	USA	12 months	Asthma	Area of deprivation: residents of a neighbourhood in which 20% of households had incomes of less than the federal poverty level	301 (151/150)	49 (13)	270 (89.7)	African American (75.4)	Mean difference in Asthma Control Questionnaire score	The intervention had greater reduction in ACQ score, but the difference was not statistically significant	NP
^64^	Krieger *et al*., 2015	USA	12 months	Asthma	Low income: household income of less than 250% of the federal poverty level (2007)	366 (177/189)	41.3	268 (73.2)	Multi-ethnic	‘Symptom-free days’ over 2 weeks	The intervention group had significantly greater and clinically meaningful increases in symptom-free days compared to control	<0.001
^65^	Martin *et al*., 2009	USA	3 months	Asthma	Low income	42 (20/22)	33 (9) versus 37 (8)	29 (69.05)	African American (92.86)	Asthma self-efficacy score	Self-efficacy increased in the intervention group and either remained the same or decreased in the control group, controlling for baseline variables	<0.001
^66^	Young *et al*., 2012	USA	6 months	Asthma	Income less than or equal to 200% of the federal poverty level	98 (49/49)	44.6 (15.8)	75 (76.5)	White (92.9%)	Patients’ asthma control (Asthma Control Test (ACT))	Results did not indicate a significant difference between the control and intervention groups	NP
^67^	Evans-Hudnall *et al*., 2014	USA	4 weeks	Stroke	Low income and education and underinsured	52 (27/25)	56.03 (9.9)/46.95 (10.74)	20 (38.5%)	African American (57)	Tobacco use (Behavioral Surveillance Survey [BRFSS])	There was a greater proportion of patients with treatment-compliant tobacco use in the intervention group compared to control	0.01
^68^	Kronish *et al*., 2014	USA	6 months	Stroke	Low income	600 (301/299)	63 (11)	354 (59)	Multi-ethnic: Hispanic/Latino and African American (86)	Proportion of sample who achieved a composite outcome of control of blood pressure lipids and regular use of antithrombotic medication	There was no difference in the proportion of intervention and control participants who at 6 months had attained their composite control measure	0.98
^69^	Tiliakos *et al*., 2013	USA	6 months	Rheumatoid arthritis	Low income	104 (52/52)	53.55	82 (79)	African American (90)	Proportion of patients who achieved 20% improvement from baseline according to the American College of Rheumatology (ACR20)	The test for interaction between intervention group and time was not statistically significant	0.7
^70^	Eakin *et al*., 2007	USA	6 months	Two or more chronic conditions	Low income	200 (101/99)	50 (13)/49 (13)	157 (78.5%)	Hispanic/Latino (80.2/1.1)	Dietary behaviour (Kristal Fat and Fibre Behavior Questionnaire [FFB])	The intervention group showed a significantly greater improvement in dietary behaviour compared to the control group	*P* = 0.003
^71^	Kangovi *et al*., 2017	USA	6 months	Two or more chronic conditions	Area with high poverty rate, underinsured/publicly insured	302 (150/152)	56.3 (13.1)	228 (75.5)	African American (94.7)	Mean change in score of participant’s chosen parameter (HbA1c, BMI, SBP and number of cigarettes per day)	There were positive differences in the 6-month change in chronic disease parameters, favouring the intervention arm	0.08
^72^	Kennedy *et al*., 2013	UK	12 months	Irritable bowel syndrome, chronic obstructive pulmonary disease or type 2 diabetes	Area of high deprivation	5599 (2295/3304)	NP	2990 (53.5)	White (96.7)	Change in shared decision making (health care climate questionnaire)	There was no difference between the groups	0.66
^73^	McKee *et al*., 2011	USA	6 months	Hypertension and type 2 diabetes	Low income	55 (31/24)	61.2 (11.2)/58.6 (7.9)	36 (65.45)	Hispanic/Latino (72.73)	Change in proportion at goal for HbA1c (≤7%)^*^	A significantly larger proportion of the intervention group was at goal for HbA1c compared to control	0.049
^74^	Mercer *et al*., 2016	UK	12 months	Two or more chronic conditions	Socio-economically deprived area (Scottish Index of Multiple Deprivation)	152 (76/76)	52	85 (55.92)	NP	Mean change in patient-reported health-related quality of life (EQ-5D-5L).	Positive improvements in quality of life favoured the intervention group at 12 months. However, the overall effect size was not significant	0.15
^75^	Riley *et al*., 2001	USA	1 month	1 or more chronic diseases	Low income	28 (15/13)	58 (9.5)	23 (82)	Anglo-white (55)	Use of social-environmental resources (Chronic Illness Resources Survey [CIRS])	The intervention group had increased their use of social-environmental resources significantly more than those in the control group	<0.03
^76^	Swerissen *et al*., 2006	Australia	6 months	Chronic diseases (general)	Low income	474 (320/154)	66 (9.52)	355 (74.9)	Multi-ethnic (Greek, Vietnamese, Chinese, Italian)	Health status (self-rated health)	At 6 months, the intervention group had a better mean self-rated health score compared to control	0.000
^77^	Willard-grace-2015	USA	12 months	Hypertension and/or hyperlipidaemia and/or diabetes	Low income, uninsured or publicly insured	441 (224/217)	52.7 (11.1)	244 (55.3)	Latino/Hispanic (70.1)	Composite clinical outcome measure—proportion of treatment group with improvement in either HbA1C, SBP or LDL according to predefined thresholds	Participants in the intervention arm were more likely than those in the control group to achieve the primary composite measure	0.02

Of the 51 studies, there were three cluster RCTs, 15 pilot studies and 33 RCTs. Three RCTs had three arms, making 54 unique interventions in total. Most trials looked at diabetes (*n* = 35). In addition, eight were on general chronic conditions or multi-morbidity, four on asthma, two on secondary stroke prevention, and one each for arthritis and depression.

### Intervention details

Table 2 describes the interventions. In addition, 25 of the 51 studies (49.0%) were underpinned by at least one named behaviour change theory. The most common were social cognitive theory (*n* = 8), the transtheoretical model (*n* = 5) and self-efficacy theory (*n* = 6). The 54 intervention arms were delivered either face to face (*n* = 23, 42.6%), remotely (*n* = 9, 16.7%) or a combination of both (*n* = 22, 40.7%). Few made use of smartphone applications (*n* = 2, 3.7%). In addition, 29 (53.7%) of the intervention arms were delivered to participants individually. The rest were delivered to groups (*n* = 12, 22.2%) or used a combination of individual and group delivery (*n* = 11, 20.4%). The most common intervention providers were community health workers (CHWs). They were also referred to as peer leaders, lay leaders and peer supporters (*n* = 22). Other intervention providers included nurses, health educators and dieticians.

**Table 2:** Intervention details

Author, date	Theory	Materials and procedures	Intervention provider(s)	Mode(s) of delivery	Setting(s)	Frequency	Low SES tailoring	Planned fidelity assessment	Actual fidelity	Financial incentives
Anderson *et al*., 2010	No	Patients received unscripted phone calls on disease management followed by mailed educational materials	Nurses were trained by a ‘master trainer’ who was an expert in commercial disease self-management	Telephone: individual	Centralised call centre	If HbA1c > 9, weekly calls, 7 < HbA1c < 9 or HbA1c < 7 with HTN/depression/retinopathy/neuropathy bi-weekly calls and HbA1c < 7 monthly calls	Educational materials were available in English and Spanish and at fourth grade reading level	Nurses documented phone encounters on patients’ electronic heath record. Intervention fidelity monitored through chart review by project co-ordinator	NP	$25 gift card to a local store after completing their 6- and 12-month assessments
Arora *et al*., 2013	No	Participants received unidirectional, SMS text messages sent. text messages were based on content from the National Diabetes Education Program	Automated	Text messages: individual	Remote	Two daily text messages (9 am and 5 pm) over 6 months. Each text is a 160-character phrase	Texts were available in English and Spanish at fifth grade reading level. If needed patients were financially compensated ($20 per month) to upgrade to an unlimited messaging plan on the phones	No	NP	$175 during 6 months for time and travel costs associated with study follow-up visits
Baig *et al*., 2015	Self-determination theory, social cognitive theory and the transtheoretical model (stages of change)	Patients received a faith-based diabetes self-management education program (DSME)	Trained, lay leaders, who either had diabetes themselves or knew a friend or family member with diabetes. Lay leaders underwent three 3 hour training sessions on coaching skills through modelling, program content, feedback and role play	Face to face: group	Churches	Eight sessions weekly, 90 min each	The DSME was faith based and culturally tailored. Lay leaders were bilingual in English and Spanish	Members of the academic team observed the class leaders during the first 8-week class and then periodically to ensure intervention fidelity using standard processes including checklists and direct observation	NP	No
Berry *et al*., 2016	No	Patients received group diabetes self-management education	Health nurse practitioner, a physician, a postdoctoral fellow and a trained interventionist	Face to face: group	Community health centre	Five sessions—one session every 3 months	NP	NP	NP	No
Chamany *et al*., 2015	Self-efficacy theory and the transtheoretical model (stages of change)	Patients were mailed a ‘welcome’ packet that included print materials on diabetes self-management and healthy retention incentives such as pedometers. Also, they received self-management support via telephone	Health educators who received 20 h of training in delivering behavioural counselling by phone. Health educators also attended a 10 h American Diabetes Association–recognized diabetes self-management program. They were supervised by a team consisting of a nurse-certified diabetes educator, internal medicine physician and clinical health psychologist thoroughly weekly case meetings	Telephone: individual	Remote	Four calls (one every 3 months) over 12 months if baseline HbA1c was in the >7.0% and <9.0%, or eight calls over 12 months if HbA1c was >9.0%	The health educators were bilingual in English and Spanish, and the print materials were adapted for low literacy	Fidelity to the protocol was enhanced by the use of telephone log sheets for documenting details of every call. Also, every study participant had a protocol flow sheet with exact dates by which protocol activities had to occur	NP	No
Clancy *et al*., 2007	No	Patients received group medical visits	The groups were co-led by an internal medicine physician and a registered nurse, modelling the format of Cooperative Health Care Clinics (CHCC). They were trained by a senior internist who had previous experience in group visits. Also, the previous trainer for CHCC providers, gave a 3 h educational training session to clinic staff	Face to face: group	Clinic	2 h group sessions, delivered monthly over 12 months	No	No	NP	A visit deposit fee per visit of $15 for intervention patients and $45 for control group patients
Davis *et al*., 2010	Health belief model and transtheoretical model (stages of change)	Patients received remote DSME, with content based off the ‘Pounds Off With Empowerment’ materials, the ADA (American Diabetes Association guidelines) and the Michigan Diabetes Research and Training Center’s Life with Diabetes Curriculum	A self-management education team, consisting of a nurse/certified diabetes educator and a dietitian	Face to face: group and video conferencing: group and individual	Academic health centre (for the providers) and primary care clinic (for the patients)	Over 12 months, there were 13 sessions in total, with two being held in the first month (one group and one individually). Sessions were monthly thereafter. 10 sessions were group based, and the remaining three were individual	Modifications included considerations for a low-literacy and a rural population	No	NP	Participants were given a gift card for each of the three completed visits
Fitzpatrick *et al*., 2022	No	Patients received resource navigation in addition to a problem-solving based, ADA recognized DSME programme	Community health workers (CHWs) who were already embedded in culturally specific community-based organizations. CHWs received 20 h of training in diabetes, delivering the DSME programme and addressing social needs	Face to face and telephone: individual	Patients’ homes or community settings (churches, cafes, libraries)	9 modules were delivered over the 6 months on a weekly and bi-weekly basis. Resource navigation support was provided as needed	The DSME curriculum was adapted for low literacy. All materials were available in English and Spanish and the CHWs were ethnically diverse	A random selection of CHW visits were audio-recorded and reviewed as a check for fidelity	NP	Participants were given a $50 gift card for completing the 6-month follow-up
Fortmann *et al*., 2017	No	Patients received an m-health SMS-text-based self-management intervention. Text message content was based on the Project Dulce DSME curriculum. The text bank included 119 different messages, less than 160 characters in length	Bilingual study co-ordinator	Text messages: individual	Remote—texts were sent out via a contracted patient health management technology platform	At the start of the intervention period, texts were 2–3 times per day at standardized h. Frequency tapered off as the study progressed	Patients who did not have a mobile-phone with texting capability were provided free of charge. Those with their own phones had the costs of the additional texts covered by the study ($12/month). Texts were in English and Spanish	No	NP	Participants received incentives at baseline, 3-month and 6-month assessment
Frosch *et al*., 2011	No	Patients received one 24-min-long DVD program with an accompanying booklet called ‘Living with Diabetes: Making Lifestyle Changes to Last a Lifetime’, which was developed by the Foundation for Informed Medical Decision Making. Patients could also receive additional telephone support	A nurse educator trained in patient-centred approaches to diabetes management and motivational enhancement	Mail and telephone: individual	Patients receive the calls and material remotely, from their homes	Five phone calls in total. Call 1 being up to 60 min. Calls 2 and 3 up to 30 min. Calls 4 and 5 up to 15 min. Patients could receive no more than 150 min (2.5 h) worth of telephone support. The time interval between calls was at the discretion of the patients and nurse educator	The nurse educator was bilingual in English and Spanish	To improve fidelity, patients received a call 1 week after enrolment in the study to remind them to review the intervention materials provided and again to schedule a telephone session. Fidelity was assessed as the number of phone sessions each patient underwent	73.0% completed five sessions of telephone coaching. The mean (SD) number of sessions completed was 4.0 (1.9)	No
Gary *et al*., 2009	PRECEED-PROCEED Framework	Patients received individualised care and self-management support, in the form of intervention action plans (IAPs) based on a clinical algorithm	Both nurse case managers (NCMs) ad community health workers (CHWs) received 6 weeks’ worth of training. CHWs continued to have weekly meetings with the project co-ordinator to reinforce initial training and go over any problems	Face to face and telephone: individual	Clinic, patients' homes, community settings or remote	NCMs conduct a minimum of one face-to-face clinic visit per patient per year. CHWs conducted home visits at least 3 times a year. However, the frequency and intensity of the intervention for each patient is guided by the algorithm which triages them according the diabetes control level. For example, those with ‘poor control’ will receive weekly contact versus every other week for those with optimal control	CHWs are also African American	No	NP	No
Greenhalgh *et al*., 2011	No	Participants took part in a semi-structured, informal group story-telling intervention, each session based around themes. Participants shared the experiences self-managing diabetes as it related to the group-selected theme	The story-telling facilitator (bilingual health advocate [BHA]) was a non-clinical professional or volunteer trained in the sharing stories model. Medical professionals such as a dietician, exercise specialist or diabetes nurse were invited to one-off sessions on a case-by-case basis	Face to face: group	Informal community settings	Each session lasted 2 h and was held every 2 weeks for 6 months	BHAs were bilingual, groups were offered in Bengali, Tamil, Punjabi, Urdu, Gujaratis and English. Those with mobility needs were offered minicab transport, allowing ‘housebound’ patients the opportunity to join the study	A researcher attended all but 8 of the 72 story-sharing sessions and checked that they followed the established protocol and format	NP	No
Hill-briggs *et al*., 2011	D’Zurilla and Nezu problem-solving therapy	Patients received a diabetes and CVD education session and problem-solving training sessions. Patients also received two workbooks: ‘Diabetes and Your Heart Facts & Information Workbook’ and ‘Hitting the Targets for Diabetes and Your Heart: Your Problem-Solving Workbook’	The interventionists underwent training and followed prepared manuals	Face to face: group	NP	One education session and 8 problem-solving training sessions, each lasting 90 min, delivered biweekly	The sessions and materials (workbooks) were adapted for accessibility and usability in low literacy and functionally impaired populations. The workbooks made use of colours (red vs green) and symbols to simplify concepts	All sessions were audiotaped, and randomly selected audiotapes were reviewed	NP	No
Lynch *et al*., 2014	Information processing model.	Participants received a community-based, group intervention that focused on diet and physical activity, and follow-up peer support	Classes were facilitated by a registered dietitian, who was assisted by two peer supporters. Peer supporters trained weekly for 8 weeks (2 h per week) with a psychologist, dietitian and health educator. Training sessions familiarized the peer supporters with goal setting and the nutrition education materials	Face to face: group and telephone: individual	Local city park building near the recruitment clinic	18, 2 h LIFE classes, which were weekly for the first 3 months and every other week for the second 3 months. Telephone support was weekly	Peer supporters were also African American and had diabetes or hypertension. They came from the same community as the participants	No	NP	No
lynch *et al*., 2018	Cognitive-behavioural models of behaviour change and information processing model	Participants received group-based DSME and individualized peer support. The bulk of the LIFE intervention curriculum focused on diet change and goals. It was based on a modified plate method referred to as ‘the Plate of LIFE’. Participants also received educational materials and workbooks	The intervention team for each group session consisted of a registered dietitian, a group facilitator and 1–2 peer supporters. A clinical psychologist supervised peer supporters. Peer supporters completed 8 h of training. They were trained to reinforce progress on goals with verbal praise and apply simplified motivational interviewing and problem-solving techniques. Peer supporters provided telephone support	Face to face: group and telephone: individual	Community settings near the main clinic	28, 2 h group sessions over 12 months: weekly for the first 4 months, biweekly for the second 4 months and monthly for the third 4 months. Two additional maintenance sessions were held at months 15 and 18. Telephone support was delivered at the same frequency	Peer supporters were also African American and the curriculum was culturally tailored. To address literacy barriers, the sessions and materials made use of graphics, simplified food lists, and physical demonstrations and hands-on activities to reinforce more abstract concepts. Numeracy barriers were addressed by repeated visual and tangible exercises counting out carb portions (using real food)	Yes: fidelity was monitored using checklists developed for each session to assess content delivery. Group sessions were recorded using a digital voice recorder. The project director reviewed fidelity data and provided feedback	NP	US$100 for each of the three full assessment and $25 for brief assessments
Nelson *et al*., 2017	Self-efficacy theory	Patients received home visits where their current diabetes self-management was assessed using a structured interview. Community health workers (CHWs) worked with participants to set health goals and develop an action plan. Participants also received complimentary educational materials	Community health workers (CHWs) who received 60 h of mandatory training in health coaching and motivational interviewing by a professional health coach and training in how to use a blood pressure monitor. Each CHW passed a competency test prior to intervention delivery	Face to face: individual	Participants’ homes	4 mandatory home visits that took place 0.5, 1.5, 3.5 and 7 months after enrolment. There was a fifth optional visit at month 10	The CHWs were bilingual in English and Spanish and educational materials were available in both languages. Materials were also adapted for those with low literacy	Yes: CHWs completed an encounter form after each visit. The forms were reviewed monthly by a certified diabetes educator to ensure that each participant receives the required components of the intervention	NP	US$25 at both baseline and 12-month assessment
Pérez-Escamilla *et al*. 2015	Transtheoretical model (stages of change) and problem solving theory	Participants received home visits were they were taught the DIALBEST curriculum in modules and received a tailored self-management plan	Community healthcare workers (CHWs) (nurse and medical assistant) who were employed by a community-based non-profit organization. They received 65 h of core training and 25 h of supplementary training by an interdisciplinary team of academics and practitioners in topics such as diabetes pathology, lifestyle strategies for glycaemic control, motivational interviewing, communication skills and social determinants of health	Face to face: individual	Participants’ homes	17 visits over a 12-month period. Visits were weekly during the first month, biweekly during months 2 and 3, and monthly until month 12	CHWs were bicultural/bilingual in English and Spanish. The curriculum was designed to be both culturally and health literacy appropriate. The self-management plans were individually tailored to meet the participants’ socio-economic circumstances	Yes: an ancillary study was conducted to audit the CHW progress notes and phone records to document intervention fidelity	Over half of the participants (51%) received the scheduled 17 visits, with an average duration of 87.8 (18.2) min per home visit	No
Philis-Tsimikas *et al*., 2011	No	Participants received diabetes self-management education based on the Project Dulce ‘Diabetes among friends’ curriculum	Peer educators (PE) known as ‘promotoras’. They were individuals with diabetes, identified as ‘natural leaders’ from a patient population. Over a 3-month period, they received 40 h of training in the curriculum, group instruction, mediation and behaviour change techniques	Face to face: group and telephone: individual	NP	8, 2 h group classes, delivered weekly and then monthly support groups. Telephone contact was made before each class to increase attendance	PEs were bilingual in English and Spanish	Yes: to ensure the fidelity of intervention delivery, all classes were audio-recorded and reviewed using checklists to monitor the delivery or omission of curriculum component	NP	Yes: participants were given small gift cards at each of the three assessment points (amount not disclosed)
Protheroe *et al*., 2016	No	Patients received an individualized self-management plan, following an interview with a lay health trainer, along with follow-up telephone support and a printed pamphlet. The interview identified areas for improvement in their health	Lay health trainers (LHTs) received training from the research team on evidence-based diabetes care and appropriate lifestyle advice	Face to face and telephone: individual	NP and remote	1 interview took place at the start of the intervention period, followed by up to 3 2-monthly support phone calls	Pamphlets were adapted for low health literacy	No	NP	No
Pyatak *et al*., 2018	Social-ecological model of health behaviour and complexity theory	Participants received an adaptation of the Lifestyle Redesign OT intervention framework, which involves a manualized, individually tailored intervention, composed of 7 flexible modules	Two licensed occupational therapists (OTs) who received 20 h of training in the intervention manual, 12 h of training in motivational interviewing and 20 h training in diabetes self-management education. An endocrinologist and a licensed clinical social worker were available on an as-needed basis for issues identified that were outside the main scope of the intervention	Face to face: individual	Participants’ homes and community settings	12 biweekly sessions averaging 1 h each, over 6 months. Timings were flexible; however, the aim was to deliver an intervention dose of between 10 and 16 h per participant	NP	Intervention fidelity was maintained through three strategies. First, therapists documented intervention dose, timing and treatment activities in notes completed after each session. Second, approximately 10% of sessions were observed by a second therapist trained in the intervention protocol, who completed a fidelity checklist and shared feedback with the treating therapist. Third, all team members trained in the intervention met weekly to facilitate problem-solving and prevent intervention drift	Fidelity monitoring showed that the therapists had 96% adherence to the intervention’s key components	Yes: received US$25 at baseline and US$50 at follow-up
Rosal *et al*., 2005	Social cognitive theory	Participants received an interactive self-management education program. The program involved direct instruction and modelling through a soap opera, skill-building activities, personalized goal setting and skill reinforcement activities	A nutritionist, nurse and intervention assistant were trained in the intervention’s theoretical and delivery models, intervention goals, counselling skills and use of materials	Face to face: individual and group	A community room 3 blocks from the health centre and 2 blocks away from the elder service	One initial 1 h individual session, followed by 10 weekly 2.5 to 3 h group sessions and two 15-min individual sessions that occurred during the 10-week period immediately prior to the group session	The intervention team was bilingual and the interventions used the traffic light concept and visual aids as a means of simplifying educational messages	No	NP	Participants were offered incentives equivalent to US$90 for completing the assessment spread out over the three assessment points
Rosal *et al*., 2011	Social cognitive theory	Participants received an interactive self-management education program. The program involved direct instruction and modelling through a soap opera, skill-building activities, personalized goal-setting and skill reinforcement activities	The intervention delivery team consisted of two leaders and an assistant (either a nutritionist or health educator and trained lay individuals or three lay individuals). The intervention staff received approximately 40 h of extensive training in accordance with a protocol that covered diabetes self-management, the theoretical foundation of the intervention and group management skills	Face to face: individual and group	Participants home (first individual session only) and community settings such as senior centres and local YMCAs	One 1 h individual session followed by 11 weekly 2.5 h group sessions and 8 monthly group sessions. Each group session included a 10-min one-to-one session for each participant with one of the intervention teams	The intervention was culturally and language tailored (English and Spanish). The content was adapted for low literacy by simplifying concepts, minimizing didactic instruction and using picture and colour-coded based guides	Yes: fidelity checklists monitored delivery or omissions of intervention components. Supervision of interventionists included a review of completed checklists following the sessions	NP	No
Ruggiero *et al*., 2014	Transtheoretical model (stages of change) and empowerment theory	Patients received a self-care coaching intervention. The aim was to help the patients learn the necessary information and skills to make informed self-care goals and changes, using the 5As framework and motivational interviewing as the primary coaching methods. They were also provided with written materials matched to their stage of change in the framework	Medical assistants served as medical assistant coaches (MACs). In addition to the standard medical assistant training, they received more than 40 h of initial project training and ongoing boosters. They were trained by the multidisciplinary team in diabetes self-management, behavioural counselling strategies guided by the theory, motivational interviewing and the 5As framework	Face to face and telephone: individual	The clinic and remote	Face-to-face clinic visits were delivered quarterly during routine diabetes visits at the clinic and were less than 30 min in length. Telephone follow-ups were monthly and less than 15 min in length	MACs were of the same ethnicity (African American or Hispanic/Latino) as the patients at their clinic, educational materials were culturally tailored, written at a fifth grade or below reading level and were available in both English and Spanish	Yes: the PI and project coordinator reviewed and tracked intervention reports, notes and charts. There was occasional direct observation by a trained research assistant and periodic PI observation of the ongoing training	The majority of patients did not receive the intended dose of the intervention	US$20 cash for baseline assessment and US$25 cash for the two follow-up assessments
Schillinger *et al*., 2009	Self-efficacy theory	Patients received individual action plans and took part in collaborative goal setting. This was achieved through an Automated Telephone Self-Management Support System (ATSM), where patients responded to automated queries regarding their self-care. Action plans and interactions are linked to the patients' clinic record	IDEALL clinical staff, including the nurse diabetes care managers for the ATSM, were trained in model protocols, motivational interviewing and communication techniques for patients with limited literacy	Telephone: individual	Remote—patients received calls in their homes	Weekly calls over 39 weeks (9 months). Each call takes between 6 and 10 min to complete	Intervention was delivered in English, Spanish or Cantonese	No	NP	US$15 and US$25 at baseline and 1-year follow-up, respectively. No additional incentive to answer calls
		Patients received individual action plans and took part in collaborative goal setting. This was achieved through Group Medical Visits (GMVs). The visits involved discussing concerns, problems or progress with the plans and modelling of self-management behaviours	IDEALL clinical staff for GMV included a physician, pharmacist and health educator. In addition to the training described above, they received training in group facilitation techniques	Face to face: group	NP	Monthly visits of 9 months, each lasting approximately 90 min	Intervention was delivered in English, Spanish or Cantonese. Participants were given bus tokens to assist with transportation costs	No	NP	US$15 and US$25 at baseline and 1-year follow-up, respectively
Schoenberg *et al*., 2017	No	Patients received a hybrid model of diabetes self-management classes (with goal setting) combined with care navigation based on the chronic care model	Trained community health workers (CHWs)	Face to face: group	Field office	The 6 classes took place every 2 or 3 weeks	The classes were culturally appropriate and material was delivered at a fifth grade or below reading level	No	NP	US$25 at both baseline and follow-up assessment
Seligman *et al*., 2018	No	Patients received food packages and DSME modelled on the American Association of Diabetes Educators AADE7 Self-Care Behaviours and adapted from components of the Type 2 Diabetes BASICS curriculum	Food bank staff, volunteers and health educators. Educators were food bank staff trained in curriculum delivery by a registered nurse and diabetes educator. Staff also received training in the following subjects: diabetes pathophysiology, screening, evaluation and treatment, client privacy and HIPAA regulations, universal precautions and sharps safety, medical waste handling and use of specific study equipment	Face to face: group	Food bank	2 mandatory group classes in the first 2 months of the intervention period, each lasting between 2 and 2.5 h. Optional ‘drop-in’ monthly sessions were available for the next 4 months and were 60 to 90 min long. Participants could receive 11 food packages picked up twice monthly over the 6-month intervention period	Classes and material were available in English and Spanish. The DSME curriculum and intervention was tailored to address literacy, numeracy, transportation barriers and costs, food-access barriers and food insecurity	No	No	US$15 gift cards at each assessment
Shea *et al*., 2006	Social cognitive theory	Patients received telehealth case management via a home telemedicine unit (HTU), which consisted of a web-enabled computer with video conferencing capabilities	Nurse case managers and dieticians conducted the tele-health visits	HTU video: individual	Remote	Every 4–6 weeks across a 5-year period	The intervention providers for ethnic minority patients were bilingual in English and Spanish and were Hispanic/Latino or African American so advice could be tailored to the patients cultural background	No	NP	No
Sixta and Ostwald, 2008	No	Patients received a diabetes self-management course, according to a scripted course curriculum	Promotores, employed by the clinic, led the course sessions in pairs, supervised by nurses. The nursing director oversaw quality control and promotores' education and training	Face to face: group	Community clinic	10, 1.5 h sessions held weekly	The course curriculum was presented in Spanish, was culturally sensitive and used pictures to aid understanding	No	NP	No
Skelly *et al*., 2009	No	Patients received a symptom-focused diabetes intervention (teaching and counselling) based on the University of California, San-Francisco symptom management model. Half of patients also received a telephone booster, reinforcing the content and strategies of the home visit	Nurses	Face to face and telephone: individual	Patient homes and remote	The 4 home visits were 60 min and took place bimonthly. The 4 telephone boosters took place 3 months after the last visit and occurred every 2–3 weeks. Each call averaged 15 min in length	The teaching was individualized and made specific to each patient home and community. Also, the visits took place at the patients' homes to avoid transport-related barriers	No	NP	No
Spencer *et al*., 2018	Social cognitive theory	Participants received an empowerment-based group diabetes self-management education (DSME) classes, based on the Racial and Ethnic Approaches to Community Health (REACH) curriculum for Latinos. In addition, they received home visits and accompanied clinic visits	Community health workers (CHWs), who underwent more than 160 h of CHW training, more than 80 h of diabetes education, including home visit experiences, training in human subjects protocols, behaviour modification strategies, cultural competency and community-based participatory research	Face to face: group and individual	Community locations, participants homes and clinics and remote	Eleven 2 h group DSME sessions held every 2 weeks, two 60-min home visits each month and 1 accompanied clinic visit over a 6-month period	The CHWs were Latinas, bilingual in English and Spanish and were from the same community/area as the participants	No	NP	No
		After the initial 6 month CHW intervention patients could receive ongoing emotional and behavioural support	Peer leaders (PLs) recruited by the CHWs. They received 46 h of training over 12-weeks and monthly booster sessions over the 12 month intervention period	Face to face: group and telephone: individual	Community locations and remote	Group drop-in sessions held weekly over a 12-month period. PLs made calls to any participants who had not attended three sessions in a row	Peer leaders were from the same community as participants but had already done the DSME curriculum previously	No	No	No
Talavera *et al*., 2021	No	Patients received a team-based integrated care and behavioural intervention based on the 5As framework. It consisted of a medical visit, behaviour visit, group DSME classes and care co-ordination. The DSME class curriculum materials were developed as an adaptation of the Pasos Adelante/Steps Forward intervention	The team consisted of a physician/medical provider, a specialty behaviour health provider and a peer health educators	Face to face: individual (medical and behaviour visits) and group (self-management classes)	The partnership clinic	4 medical and behaviour visits over a 6-month period and six 2 h DSME self-management classes	All intervention providers were bilingual in English and Spanish and Latino. DSME classes emphasized visuals and minimal text to accommodate varied levels of literacy	Yes: the number of medical and behavioural visits and DSME classes were tracked. DSME classes were audiotape recorded and reviewed by a trained research assistant who used a checklist to evaluate coverage of key content and ensure delivery as intended. The behavioural health providers completed a checklist based on the 5As framework after each visit	No major deviations. Fidelity by the 5As framework showed the following: Assess (99%), Advise (96%), Agree (78%), Assist (75–97%, depending on the topic) and Arrange (79%). 47 participants received no intervention contact	No
Thom *et al*., 2013	No	Patients received peer health coaching	Peer health coaches who were patients at the clinic, who had an HbA1c level of less than 8.5% within the past 6 months. They received 36 h of training over 8 weeks, conducted by two of the research investigators. Trainees who passed both a written and an oral examination became peer coaches. Trainees received US$150 for completing the training, regardless of if they passed	Face to face and telephone: individual	The public health clinic and remote	Telephone contact must be at least twice a month and in-person contact at least 2 times during the 6-month intervention period	Peer coaches were from the same clinic/community and spoke either English or Spanish	No	NP	Patients received US$10 after baseline data collection
Wang *et al*., 2018	Social learning theory and self-regulation theory	In addition to usual diabetes care and education, participants received lifestyle-based intervention sessions and tracked progress using a series of mobile apps (Diabetes Connect app and LoseIt!)	Lifestyle counsellors were trained using publicly available materials and a digital optical disc and printed training materials from the Group Lifestyle Balance (GLB) program and the Look AHEAD intervention	Face to face and mobile: group and individual	Community centre	11 group sessions: weekly for month 1, biweekly for months 2 and 3, and monthly for months 4 to 6—and an individual session after month 3. Each session was 1 to 2 h	All intervention materials were modified to be at ninth grade reading level. Also, participants without a smartphone were lent one for the study duration	A checklist was used for each group and individual session to track the content delivered. The principal investigator (PI) attended at least 80% of the group sessions for both paper and mobile groups to ensure treatment fidelity	NP	No
		In addition to usual diabetes care and education, participants received lifestyle-based intervention sessions and tracked progress using CalorieKing food and exercise journals					All intervention materials were modified to be at ninth grade reading level.			
Wayne *et al*., 2015	Intervention described as theory driven but details not provided	Participants received health coaching with additional mobile phone support, based on a behaviour change curriculum co-designed by the study authors. The app used was the Connected Wellness Platform (CWP) provided by NexJ Systems, Inc	Health coaches who were behaviour change counselling specialists, with expertise in chronic disease management. They were either certified exercise physiologists or personal trainers. All coaches attended weekly seminars and meeting prior to and throughout the trial for training in the curriculum	Mobile app and face to face: individual	Remote	App communication was 24 h a day/7 days a week basis	Participants were provided with a Samsung Galaxy Ace II mobile phone during the intervention period. Also, the health coaching curriculum was adapted for the socio-economic context and ethnocultural backgrounds	No	Mean contact time between participants and health coaches was 38 min/week (SD 25)	No
Whittemore-2020	Social cognitive theory, Empowerment theory and Health Action Process Approach Model (HAPA)	Participants received education sessions supplemented by text messages	The group session coordinators were a registered nurse and social worker who received one week's worth of training. The training program consisted of content on the program and its theoretical underpinnings, the pathophysiology and treatment of type 2 diabetes, and the social determinants of health in Mexico City	Face to face: group, text messages and telephone calls: individual	In 5 Seguro Popular clinics	Seven group sessions, which were followed up by a phone call every 2 weeks and daily text messages	Texts were written at a third to fourth grade reading level, with simple pictures to enhance understanding. For those unable to receive texts, the text content and pictures were printed on card. The group sessions were made to be culturally relevant and appropriate for adults with low health literacy. Group activities were tailed to the cultural and socioeconomic context of participants	A 5-item fidelity checklist and attendance were completed by the group session leaders. Also, approximately 35% of sessions were observed by a trained research assistant to ensure protocol fidelity	Average group session attendance was 89%. 100% of participants received texts at 6 months (96% at 3 months). 88% received picture messages at 6 months (83% at 3 months)	Participants received department store gift cards after each data collection point—$200 Mexican pesos (∼$10 USD) at baseline, $300 Mexican pesos (∼$15 USD) at 3 months and $400 Mexican pesos (∼$20 USD) at 6 months
Aikens *et al*., 2022	No	Patients and their nomination ‘care partner’ (CP) received depression self-management advice via an automated interactive voice response (IVR) telephone system	Automated: the structured algorithm determines which pre-recorded queries patients hear	Telephone: individual	Remote	Over a 12-month period, patients received calls weekly, with each call lasting 5 to 10 min	NP	No	No	After each of the three planned assessments, patients, their CPs and in-home supporters were offered a $50 cash card for attendance. Patients could receive up to $150 during the study
Apter *et al*., 2019	No	Patients received one-off training in patient portal use on how to locate a laboratory test result, check an upcoming doctor’s appointment, schedule an appointment, locate medication lists, find their immunization record, request a prescription refill and send a secure message. They also received home visits for care coordination and to promote their online patient portal use and familiarity with health information technology	Community health workers (CHWs) who were trained as lay health educators	Face to face: individual	Patients’ homes	Four visits over 6 months at weeks 2–4, 4–7, 6–11 and 23–27	CHWs were from the same community as the patients	No	NP	No
Krieger *et al*., 2015	Social-cognitive theory and self-regulation theory	Participants received home-based asthma education, support and service coordination. Through motivational interviewing, they developed a tailored asthma self-management plan	Community health workers (CHWs) with personal experience of asthma. They received 80 h of classroom training followed by biweekly training sessions. A health educator and nurse provided clinical support and a manager provided oversight	Face to face, telephone and email: individual	Patient homes	1 initial visit/assessment at baseline, followed by 4 follow-up visits 0.5, 1.5, 3.5 and 7 months later. Additional telephone and email support was on an as-needed basis	CHWs could speak Spanish and English and were from the same community as participants	The project nurse conducted monthly audits of home visit records. The project manager or nurse observed at least 1 home visit per month per CHW and rated it with a structured tool	90% of identified problems on each participant’s asthma problem list were addressed with the correct protocol, 86% of mandatory protocols were discussed and 83% of active problems were addressed at each visit	US$35 and US$50 for completing baseline and exit data collection
Martin *et al*., 2009	Self-efficacy theory and social learning theory	Patients received group education sessions and home visits and co-developed an asthma self-management plan. Sessions and home visits involved environmental restructuring, problem solving, and asthma related goal setting as mechanisms for improving self-management skills.	A social worker led the group sessions with the support from community health workers (CHWs). CHWs delivered the home visits. CHWs were trained to establish relationships with participants, successfully implement home visits, and teach basic asthma facts, skills, and self-management techniques. The social worker was trained to effectively lead self-management group sessions and to supervise the CHWs. Altogether both the CHWs and social worker received 113 h of training. CHWs were evaluated by study investigators using a standardized role-play scenario to determine their readiness.	Face to face: individual and group	Primary care clinic (group sessions) and patients’ homes	4 group sessions (2 h each) and 4 to 6 CHW home visits over a 12-week period	NP	CHWs and investigators met weekly throughout the study implementation phase to review documentation	CHWs reported covering all the required areas of asthma education, with the most emphasis on controller medications and taking medications correctly	Participants received US$25 after attending each group session and were mailed US$10 after each home visit
Young *et al*., 2012	Self-efficacy theory	Patients receive counselling based off materials from the Indian Health Services’ patient-counselling model and the pharmacist–patient consultation program, which formed the communication guide	Trained pharmacists who were certified in the National Asthma Educator Certification Board Exam. Pharmacists were trained by a patient–provider communication expert	Telephone: individual	Remote—patients received calls in their homes	Three phone calls over a 3-month period	NP	During the intervention, pharmacists were evaluated during the intervention by a health communication scientist to examine their fidelity. Using the standardized counselling framework as a guide, the scientist reviewed and commented on the pharmacists’ adherence to the protocol	NP	Participants were reimbursed $75 for study participation: $50 at the beginning and $25 at study completion
Evans-Hudnall *et al*., 2014	No	Patients received self-care sessions, with content based on the AHA (American Heart Association’s) guidelines and the 5As framework and cognitive behavioural therapy (CBT)	A health educator with a bachelor’s degree in health education and several years’ experience conducting chronic illness self-care education sessions. They also received training in stroke-specific health problems and CBT techniques	Face to face and telephone: individual	Intensive care unit and remotely in patient's homes	One face-to-face session at the start of the program and two phone calls, one every 2 weeks, for 4 weeks. Each session was 30–45 min	The health educator made sure to recommend free and easily accessible resources to aid in the adoption and maintenance of the specified goals. Materials were culturally tailored based on the religiosity and collectivism constructs prevalent amongst African Americans and Hispanics	No	NP	No
Kronish *et al*., 2014	No	Participants received a peer-led stroke prevention group–based workshop adapted from the Chronic Disease Self-Management Program. After each session, participants were required to make an action plan with a goal	Peer leaders who received 5 days of training in the Stanford Program’s philosophy and methods. The trainers observed the first course and 20% of subsequent courses taught by new peer leaders	Face to face: group	Community settings	6 weekly workshops, each 90 min in length	Peer leader were from similar socio-economic backgrounds as the participants. Workshops and materials were available in both English and Spanish. Concepts were taught in terms lay people could understand	No	NP	No
Tiliakos *et al*., 2013	No	Patients received an arthritis self-management program (ASMP). The ASMP was supplemented by a printed educational manual	An instructor	Face to face: group	The hospital	Weekly 2 h sessions over 6 weeks	The education manual was at an eighth grade reading level	No	NP	No
Eakin *et al*., 2007	No	Patients received a lifestyle intervention based off of the 5As framework advocated in multiple behavioural risk factor interventions (Ask, Assess, Advise, Agree, Arrange)	An experienced health educator	Face to face, telephone and newsletters: individual	Clinic or patient’s home for face-to-face visits, depending on patient preference	Two face-to-face visits lasting 60–90 min, 3 months apart. Three phone calls, two after the first visit (2 and 6 weeks after) and one after the second visit (2 weeks after). Three newsletters were also sent	Tailoring included the use of visual aids for low literacy, cultural adaptations and materials translated into Spanish. Health educators were also bilingual	Fidelity was assessed by tracking the delivery of the intervention protocol, including the number of intervention sessions delivered, and the percentage of patients who set goals on physical activity and dietary behaviour change	Of the 101 intervention participants, 48 (47.5%) received two visits, 39 (38.6%) received one and 14 (13.9%) could not be contacted for visits or calls. 46 (45.5%) received the three follow-up phone calls, 29 (28.7%) received two calls, 9 (8.9%) received one call and three (3.0%) were never reached for follow-up-calls	No
Kangovi *et al*., 2017	Goal setting theory	Participants underwent a collaborative goal-setting session, followed by ongoing support. The follow-up support included tailored coaching, social support, advocacy and navigation to community resources. Participants co-developed tailored action plans for their chosen goals with community health workers. They could also attend group support sessions	Research assistants and primary care providers provided the collaborative goal-setting and were offered a 60-min training session. Community health workers (CHWs) delivered the follow-up support. They underwent a month-long college accredited training course covering topics such as action planning and motivational interviewing. CHWs were supervised by a manager, who was typically a master’s level social worker	Face to face: group and individual, text and telephone: individual	Primary care clinic (weekly support group), participants’ homes and community settings	1 collaborative goal-setting session at baseline. Follow-up support from CHWs was delivered at least once weekly through various forms of communication. The support groups were weekly, but optional over the 6-month period	Collaborative goal setting made use of visual aids. Each individual plan was developed with the social determinants of health in mind	Research assistants were observed during an initial training period to assess fidelity to the collaborative goal-setting scripts. Managers assessed fidelity of the CHW support component through a recurring series of weekly assessments such as chart review, quarterly day-long observation, calls to patients to hear about their experience and a performance dashboard	Patients and CHWs created an average of 4.6 action plans over the course of their 6-month relationship. These action plans most commonly related to health behaviour changes (58.9%) and psychosocial issues (23.5%)	US$10 pre-paid gift card upon completion of the baseline survey, US$20 upon completion of baseline laboratory testing and US$30 upon completion of the 6-month follow-up assessment
Kennedy *et al*., 2013	Normalization process theory	Patients received a whole systems model of self-management support compared within routine primary care. The patient-centred approach to the routine management of long-term conditions focuses on providing skills, resources and motivation to patients	Primary care providers (doctors, nurses, technicians) received 2 days of training by two facilitators. The training teaches three core skills: (i) assessment of the individual patient’s self-management support needs, in terms of their current capabilities and current illness trajectory; (ii) shared decision making using the PRISMS (Patient Report Informing Self-Management Engagement) tool; (iii) facilitating patient access to support	Face to face: individual	General practice	NP	NP	Yes: fidelity checks took place after training	There were varying levels of implementation in routine practice: information guidebooks were readily used (88% of clinicians reporting use, 51% ‘regularly’) whereas the PRISMS tool was least used (42% reporting no use)	No
McKee *et al*., 2011	No	Patients received home-based self-management support focused on goal setting for behaviour change and targeted health/risk communications related to improving the ‘ABCs’ (A1c, BP and cholesterol). They also received enhanced care navigation via a tele-monitoring system, whereby home readings of blood glucose and blood pressure were sent to their primary care provider	Home health nurse (HHNs) took part in workshops to enhance skills in promoting self-management, covering selected health behaviour counselling techniques (motivational interviewing) to control primarily blood pressure, as well as glucose and lipids. The program manager was a nurse certified diabetes educator. Also, clinicians were educated to meet the clinical guidelines for HbA1c and blood pressure	Face to face and telemonitoring equipment—individual	Patient homes	NP	Intervention delivered in English or Spanish	No	25 out of 31 patients received the entire protocol. There were 10.4 home nursing visits over an average of 75.2 days (SD = 35.6), or 10.7 weeks	Participants received modest incentives for completing the research interviews, but not for home visits or telemonitoring (amount not provided)
Mercer *et al*., 2016	No	Patients received a whole-systems care intervention which included longer consultations and additional patient self-management support packs containing materials such as a cognitive behavioural therapy-derived self-help booklet	General practitioners who received training over 3 half days on how to use the longer CARE Plus structured consultations to carry out a holistic assessment. This included identification of patient concerns and priorities, a focus on self-management and agreeing on a care plan. They also had 20–30 min of mindfulness-based stress reduction	Face to face: individual	General practitioner	12-month period	NP	Yes: intervention fidelity was estimated from the details recorded on the CARE Plus care plan by practitioners and the patient-reported questionnaire data	Intervention patients received longer initial consultations, with a mean length of 36.9 min (SD 9.8), according to the care plan, and a mean of 34.1 min (12.7) according to the patient report. Mean time per patient in the CARE Plus consultation was 69.2 min (SD 30.18). Practitioners reported giving the self-management pack to 97% of patients	£5 gift voucher on completion of each questionnaire
Riley *et al*., 2001	Social cognitive theory, social-ecological theory and self-efficacy theory	Participants met with a health educator to set self-management goals, identify barriers, and supports to self-management and problem-solve. Health educators provided feedback on the CIRS score and helped to identify social-environmental resources relevant to that goal. After setting up the self-management plan, participants received follow-up support	Health educator	Face to face, telephone and newsletters: individual	Participants’ homes and remote	One visit at the start of the intervention, followed by the first newsletter immediately after. One, follow-up, 5 min, phone call took place 1 week after the visit. The second tailored newsletter was sent 5 weeks after the visit	NP	Yes: using RE-AIM Framework	All intervention components were implemented 100% as intended, with the exception that one participant did not receive the follow-up phone call	No
Swerissen *et al*. 2006	Social learning theory	Participants received the Chronic Disease Self-Management Program developed at Stanford University	Peer leaders who received 20 h of training from two master trainers prior to leading the program	Face to face: group	Community settings such as senior citizens clubs, churches and community health centres	6 weekly sessions, each session being 2.5 h long	The peer leaders were bilingual. All programs were delivered in participants’ first language (Chinese, Italian, Greek or Vietnamese)	No	NP	No
Willard-grace-2015	No	Patients receive health coaching during a three-stage medical visit which consisted of a pre-visit, a collaborative medical check-up with a clinician and a post-visit session	Medical assistants retrained as health coaches. They received 40 h of training in collaborative communication, disease-specific knowledge, medication adherence, developing actions plans and knowledge of community and clinic resources	Face to face and telephone: individual	Community clinic for face-to-face visits and remotely from patients’ homes	The clinic visits were at least once every 3 months and telephone follow-ups were at least monthly over a 12-month period	The health coaches were bilingual in English and Spanish, self-identified as Latina and had not received a 4-year college education	Number of health coach interactions per patient	Mean = 12.4, (SD = 7.4)	Participants received $10 for taking part in a 45-min pre-randomization interview

Thirty-eight of the intervention arms had outlined their specific tailoring for socioeconomically deprived groups (70.4%). The most common modification was adapting the reading material for low literacy and numeracy, for example, setting the materials at the US fourth or fifth grade reading levels (ages 9 to 11) (*n* = 16, 29.6%), including visual aids and diagrams to simplify abstract and complex concepts (*n* = 8, 14.8%) and using colour codes for health guides (*n* = 3, 5.6%). Materials were provided in both English and Spanish to accommodate the high numbers of Hispanic and Latino participants (*n* = 27, 54.0%). Also, many interventions (*n* = 22, 40.7%) were delivered by bilingual CHWs or peer leaders from the same ethnicity and community as the intervention participants, who provided culturally tailored advice based on their experiences. Finally, some of the text-messaged-based interventions had financial provisions to cover the cost of texts or provided participants a mobile phone for the study duration (*n* = 4, 7.4%). Only two (3.7%) of the face-to-face interventions had tailoring that addressed transportation barriers, for example, by providing free bus tokens or minicab transportation. These modifications were often used in combination with one another.

Of the seven self-management components (Table 3), the most frequently used was ‘lifestyle changes’ (*n* = 46, 85.2%), with most intervention arms focusing on diet and physical activity. This was followed by ‘symptom management’ (*n* = 40, 74.1%), mainly about blood glucose monitoring. Both ‘information’ and ‘drug management’ were common (both *n* = 36, 66.7%). Drug management involved medication reminders, aids such as pill boxes and sessions explaining how and why to take each drug. ‘Management of psychological consequences’ (*n* = 33, 61.1%) focused on stress management. ‘Social support’ (*n* = 32, 59.3%) usually involved sessions on family and home barriers to self-management. Families could also attend group sessions and home visits. The least used component was ‘Communication’. Only 18 (33.3%) interventions explicitly had elements involving communication strategies with primary care providers.

**Table 3:** Self-management components

	Self-management components based on Barlow *et al*., 2002							Other details including medical treatment	Control description	References for additional material
Author, year	Information	Drug management	Symptom management	Management of psychological consequences	Lifestyle changes	Social support	Communication			
Anderson *et al*., 2010	Yes: during the brief clinical assessment	Yes: problem-solving discussions around medication adherence	Yes: glucose monitoring and reviewing home results	Yes: stress management	Yes: diet, exercise, smoking cessation and developing specific goals	No	No	No	Usual care	
Arora *et al*., 2013	Yes: educational texts and trivia questions on diabetes myths	Yes: medication reminder texts	Yes: texts containing glucose monitoring, blood pressure monitoring, foot care information	No	Yes: texts containing healthy living challenges, which were specific daily challenges related to diet and food choices such as not drinking juice	Yes	No	No	Usual care	
Baig *et al*., 2015	Yes: participants were given information about diabetes	No	No	No	Yes: participants were taught healthy Mexican recipes and at-home exercises not requiring special equipment. They were informed of church sponsored exercise programs. Also taught a cognitive approach to behavioural problem solving including goal setting, anticipating obstacles, stimulus control and behavioural alternatives	Yes: social support gained from the group setting	No	No	Enhanced usual care including, one 90-min lecture on diabetes self-management	^84^
Berry *et al*., 2016	Yes: teaching patients to understand the complications of diabetes	No	Yes: teaching patients to understand blood pressure, cholesterol and blood glucose–monitoring goals before meals, after meals and long-term (A1C). Also, understanding the importance of proper foot self-examination and foot care	No	Yes: teaching patients to understand the importance of nutrition and exercise goals	No	No	Patients could also have their medications reviewed and an individual medical examination during the group sessions	Individual appointment with a nurse or physician once every 3 months for 15 months	
Chamany *et al*., 2015	No	Yes: calls addressed problem solving and self-efficacy regarding medication adherence and patients were sent supportive retention aids such as a 7-day pill box, which they were encouraged to use over the phone	No	Yes: calls could address stress management as an optional topic, depending on the participants’ preferences	Yes: calls addressed self-efficacy and problem solving regarding physical activity and exercise. Patients were also sent items such as pedometers and were encouraged to use them over the phone	No	No	No	Control patients were given the same print materials and retention items	^85^
Clancy *et al*., 2007	Yes: visits discussed the complications of diabetes	Noon to	Yes: visits discussed foot care	Yes: visits discussed the emotional aspect of diabetes	Yes: visits discussed healthy eating strategies, nutrition, and exercise	No	No	Vaccinations, foot examinations, medication adjustments. Laboratory orders and referral for retinal exams also took place during the group visits	Usual care in the tradition patient–physician dyad	
Davis *et al*., 2010	No	Yes: the video conferencing included a session titled ‘Know Your Medicines’	Yes: Session titled ‘Foot Care Basics & Know Your Numbers’. Also, participants were given logs to self-monitor their blood glucose	Yes—sessions titled ‘Stick With It: Positive Thinking’ and ‘Stress Management’	Yes: the program includes goal setting and sessions titled ‘Welcome and Health Eating’, ‘Keeping well and healthy’, ‘Be a Food Detective’, ‘Healthy Eating Out’ and ‘Shop Smart’. The last session was in-person at a local grocery shop	Yes: session titled ‘Community Resources, Social Support’	No	Optional retinal examinations	Control patients were given one 20-min diabetes education session, using ADA materials, conducted individually. They were also given access to usual care and community resources, including care managers for goal setting and education	
Fitzpatrick *et al*., 2022	Yes: modules outlined information on diabetes	Yes: modules covered medication adherence	Yes: modules outlined clinical targets for blood glucose, HDLs, LDLs and blood pressure	No	Yes: modules covered lifestyle changes such as healthy eating and physical activity	No	No	No	Control patients were emailed diabetes materials monthly and received navigation support for medical and social resources. The received 2 follow-ups over a 6-month period	^86^
Fortmann *et al*., 2017	NP	Yes: example text, ‘Tick, tock. Take your medication at the same time every day!’	Yes: participants received a blood glucose meter, test strips and instructions on use. Example texts for monitoring prompts include ‘Time to check your blood sugar. Please text back your results’; 1 value >250 or <70 mg/dL or 3 values between 181 and 250 mg/dL prompted a study coordinator to call the participant to assess possible reasons for hyperglycaemia/hypoglycaemia	NP	Yes: example text, ‘Use small plates! Portions will look larger, and you may feel more satisfied after eating’	Yes: example text, ‘Get the support you need family, friends and support groups can help you to succeed’	NP	No	Control patients also received the 15-min diabetes educational video developed by Scripps, a blood glucose meter and testing strips, with instructions. Afterwards they continued with usual care which included visits with a primary care physician, certified diabetes educator and group DSME, although the use of the services was dependent on physician and patient initiative	OTHER PROJECT DULCE INTERVENTIONS INCLUDED IN THIS STUDY
Frosch *et al*., 2011	NP^*^	NP	NP	NP	Yes: during the coaching intervention the nurse educator collaborated with participants to identify desired and attainable behavioural goals that could have a positive impact on their diabetes management. Together a behavioural plan was developed and monitored	NP	NP	NP	Control patients received a 20-page brochure entitled ‘4 Steps to Control Your Diabetes for Life’, which was developed by the National Diabetes Education Program of the National Institutes of Health	
Gary *et al*., 2009	Yes: CHWs gave patients feedback on their blood pressure and blood glucose results during home visits and provided health education that would be followed up in the IAPs	Yes: IAPs could address medication adherence such as problems understanding the prescribed regime or obtaining the drugs. Follow-up actions by the CHW involved home visits to organize and monitor pill-taking behaviour	Yes: there were IAPs on foot care and home visits involved blood glucose monitoring	No	Yes: there were nutrition and physical activity IAPs. Also, during home visits CHWs could review patients’ fridges/cabinets and take them on grocery field trips to educate them on health food choices. CHWs could also facilitate group walking exercises	Yes: during home visits CHWs could involve family members and teach supportive behaviours such as how to perform glucose monitoring for a patient with poor eyesight	No	NCMs oversaw any aspect of the intervention requiring nursing expertise such as participating in the upward titration of insulin dose and prompting the physician regarding sub-optimal care patterns	The minimal intervention involved telephone calls every 6 months to remind patients of preventive screenings and a written summary of healthcare utilization was sent to their primary care provider. They also received diabetes-related educational material in the mail	^87^
Greenhalgh *et al*., 2011	Yes: exchanging diabetes knowledge during sessions	Yes: themes such as ‘medication’	Yes: stories around foot care and symptom management	Yes: discussions around the emotional impact of diagnosis and the affect it has on identity	Yes: discussions/stories around diet and exercise	Yes: themes such as ‘feeding the family’ and discussions around ‘mobilising a care network’. The group setting also provided social opportunities	Yes: themes such as ‘dealing with doctors’	No	Participants received a nurse-led group diabetes education sessions held in the hospital or community settings	^88^
Hill-Briggs *et al*., 2011	No	Yes: the education session covered the self-management behaviours of taking medications	Yes: the education session covered control of blood sugar, blood pressure, and cholesterol and self-monitoring	Yes: one of the problems solving sessions covers how to take control of stress and emotion through adaptive thinking techniques	Yes: the education session covered eating healthy and getting physical activity	No	No	No	Control patients received a condensed version of the intervention. 1 education session and 1 problem solving session	
Lynch *et al*., 2014	No	No	Yes: LIFE classes covered blood glucose self-monitoring	No	Yes: LIFE classes focused on helping participants adapt a low-sodium, moderate-carbohydrate DASH (Dietary Approaches to Stop Hypertension) diet. Participants received a nutrition education workbook and a daily food log. They also received a pedometer and were told to set a step goal. LIFE classes included a peer supporter led moderate aerobic activity along with music	Yes: LIFE classes and telephone calls also provided emotional and social support	No	No	Two 3 h self-management training classes taught by an African American community health worker. One class focused on diabetes self-management and the other on nutrition	
lynch *et al*., 2018	No	Yes: materials covered medication adherence	Yes: participants were given glucometers and glucose test strips and a daily log to monitor results. The sessions covered information on hyperglycaemia and hypoglycaemia	Yes: materials covered healthy coping	Yes: the core of the sessions DSME curriculum was focused on healthy eating such as carbohydrate counting, reading food labels, a grocery shop tour and eating more vegetables and wholegrains etc. They used a modified version of the plate method. They were also given food logs. Participants were given resistance bands and a 10-min resistance band workout was included in every group session. They were also given an accelerometer and were encouraged to track steps and meet the 10 000 steps per day goal. Peer supporters provided encouragement through telephone follow-ups	Yes: peer supporters provided social support. The group sessions had a dedicated ‘listening session’ where participants could share their struggles as well as their communal wisdom and expertise	No	No	2 DSME sessions, delivered in the clinic, by a registered dietician in the first 6 months of the study period. Control participants also received glucometers	^89^
Nelson *et al*., 2017	Yes: two of the mandatory education topics were ‘what is diabetes?’ and ‘treating diabetes’	Yes: one of the mandatory education topics was ‘diabetes medications’	Yes: two of the mandatory education topics were ‘signs and symptoms of low and high blood sugar’ and ‘blood glucose monitoring’	NP	Yes: two of the mandatory topics were ‘food and diabetes’ and ‘diabetes and physical activity’. Optional topics/activities included attending a community kitchen or a CHW-led grocery shopping tour to demonstrate how to make economical yet healthy food choices	Yes: CHWs mobilized social support for participants by encouraging family and other members of participants’ support networks to help participants by encouraging lifestyle changes and medication adherence, attending clinic visits and providing emotional support	NP	CHWs facilitated coordination with primary care and case managers and encouraged participants to visit their provider for regular check-ups	Usual care, including medical care, community resources and one CHW visit after 12 months	^90^
Pérez-Escamilla *et al*. 2015	Yes: visit 2 was an ‘intro to diabetes’. 3 subsequent visits addressed complications of diabetes	Yes: visits discussed medication adherence, especially visit 7 ‘medications’	Yes: visits discussed diabetes complications and home glucose monitoring. They were also given a glucometer and glucose test strips	Yes: visit 11 focused on mental health	Yes: visit 10 focused on physical activity. Visits 3, 4, 5, 6, 9 and 15 focused on nutrition and related topics such as portion size and food labels. Visit 9 involved an onsite grocery shopping activity	Yes: family members, if present, were allowed to sit in during the home visits	No	CHWs had weekly meetings with the health management coordination team at the clinic, to update them on self-management barriers faced by the participants. The medical providers were able to provide feedback and suggestions	Usual care—physicians were expected to check HbA1c levels every 3 months and to conduct yearly foot, urine and eye examinations. Control participants received glucometers and glucose test strips with instructions on use. They were able to purchase medications at a discounted cost. Also, referrals to the clinic dietician were provided when needed	
Philis-Tsimikas *et al*., 2011	Yes: the curriculum covered diabetes and its complications	Yes: the curriculum medication adherence and cultural myths/beliefs interfering with management such as, fear of using insulin and nopales, such as Mexican prickly pear cactus, as cures	Yes: participants were given glucometers and test strips. The curriculum covered blood glucose monitoring and cultural myths/beliefs interfering with monitoring such as relying on urine	Yes: the curriculum covered emotional experiences	Yes: the curriculum covered diet and exercise	Yes: during classes, participants could share their experience and receive advice and social support from each other	No	PE's had access to laboratory results and if they noticed that a participant was not meeting treatment guidelines, they encouraged them to seek further help from their primary care provider but did not offer medical advice themselves	Usual care and free glucometer and test strips	
Protheroe *et al*., 2016	Yes: discussed perceptions of risk from diabetes	NP	NP	NP	Yes: discussed advantages and disadvantages of behaviour change	NP	NP	LHTs advised participants about essential health care tests and checks they should receive regularly as advised by Diabetes UK	Usual medical care, including a review by their family doctor at least once every 12 months	
Pyatak *et al*., 2018	Yes: modules 2 and 7 deal with what diabetes is, its treatment and long-term complications	Yes: module 4 ‘Activity and health’ (flexible based on participants' needs)	Yes: module 4 ‘Activity and health’ (flexible based on participants' needs)	Yes: module 6, ‘Emotions and Wellbeing’ deals with emotions such anxiety, depression, anger, guilt, denial, fear; coping with diabetes burnout and self-destructive behaviours; promoting well-being and developing positive coping strategies	Yes: module 4 ‘Activity and health’ (flexible based on participants’ needs) deals with establishing and maintaining health-promoting habits and routines such as carbohydrate counting skills	Yes: module 5 ‘Social Support’ deals with managing diabetes in social situations, dealing with ‘diabetes police’, family-household life, peer relationships and intimate relationships. In some sessions, OTs engaged with family members to resolve social support problems identified by the participant	Yes: module 3 ‘Access and Advocacy’ deals with accessing health care and self-advocacy and communication in health care and community settings	No	Attention control—included an initial home visit and 11 follow-up phone calls, delivered biweekly. Phone calls followed a script and a staff member delivered a standardized set of educational materials published by the National Diabetes Education Program and MyPlate.gov	^91^ ^,^ ^92^
Rosal *et al*., 2005	Yes: session topics included enhancing understanding of basic facts about the disease	Yes: session topics included the role of medications and adherence	Yes: session topics included adherence to daily blood glucose self-monitoring and understanding of values	Yes: session topics included stress management	Yes: session topics included dietary guideline education, menu planning and a supermarket tour. Topics also included physical activity with an emphasis on walking	Yes: session topics included family support. Family members could attend sessions as a way to elicit home-based support/approval for the participant	No	No	Control participants were given a simple booklet describing the importance of lifestyle factors in diabetes management and providing recommendations for diet, PA and blood glucose monitoring	
Rosal *et al*., 2011	Yes: session 2 covers ‘what is diabetes’, other session also touched on diabetes complications	Yes: sessions cover medication adherence	Yes: participants were given glucometers and a tracking log. Sessions cover self-monitoring of blood glucose and management of hypoglycaemia	Yes: sessions covered stress management	Yes: participants were given a step counter and were encouraged to increase their daily steps. Sessions covered physical activity and various aspects of diet such as reading food labels and portion control. Reinforcement activities included cooking healthy meals during sessions, food bingo and a supermarket tour	Yes: family and friends could attend the group sessions	Yes: sessions covered topics such as communicating and keeping in touch with health care providers and what to ask them	No	Usual care	^93^
Ruggiero *et al*., 2014	Yes: through the education materials provided	Yes: coaching content included medication adherence	Yes: coaching content included blood glucose self-monitoring and foot care	Yes: coaching content included healthy coping	Yes: coaching content included healthy eating, smoking cessation and physical activity	No	No	The MAC also supported the patient in arranging appointments and made referrals	Usual care, including regular visits with a primary health care provider, referrals for specialty care such as foot and eye examinations and basic education delivered by their physician. All participants were given the ‘Diabetes: You’re in Control’ educational booklet at the baseline	
Schillinger *et al*., 2009	Yes: health education messages in the form of narratives	Yes: medication adherence	Yes: self-monitoring of blood glucose and symptom queries	Yes: queries about psychosocial issues (e.g. coping, depressive symptoms, etc.)	Yes: queries on diet and physical activity	No	No	Care manager also facilitated referrals for preventive services (e.g. ophthalmologist, etc.)	Usual care	^94^ ^,^ ^95^
	NP	NP	NP	NP	NP	Yes: group visits included social breaks	NP	During visits patients with unmet medical needs also received brief, individualized care		
Schoenberg *et al*., 2017	Yes: class one gives an over of diabetes and its effect on the body	Yes: class covers two medications taking, to avoid diabetes complications	Yes: class two cover blood-glucose self-monitoring. Class 5 covers avoiding feet, teeth, eyes, sick days, kidneys and blood pressure complications	Yes: class four covers stress management	Yes: class three covers healthy eating and class four covers physical activity	Yes: program covers working with family (class number not specified)	Yes: program covers working with health care providers (class number not specified)	Regarding medical appointments, CHW assisted in rescheduling, arranging transportation, finding dependent care options and motivating on follow-through	Usual care	
Seligman *et al*., 2018	Yes: education materials were given with the food packages. Class topics included a disease overview	Yes: class topics included a diabetes medications overview, with medical professionals such as a registered nurse or physician as guest speakers	Yes: class topics included blood sugar monitoring	Yes: class topics included stress management and depression and healthy coping. Social workers and therapists were guest speakers for these topics	Yes: many classes covered aspects of healthy eating such as carbohydrate counting and reading food labels. Food packages contained diabetes-appropriate food and were accompanied with written healthy recipes. Classes also covered physical activity including exercise instructors as guest speakers	Yes: the class curriculum involved prompts to ask about questions family members had	No	Participants also received onsite HbA1c testing at months 3 and 6 and referrals to a primary care provider if they did not already have one	Wait list control	
Shea *et al*., 2006	Yes: via the HTU patients had access to web-based diabetes educational materials	No—not explicitly	Yes: patients could upload their blood pressure and blood glucose measurements onto the HTU, where it could then be reviewed by their case manager. Patients also set HbA1c, cholesterol and blood pressure goals with their case manager during tele-health visits	No	Yes: nurse case managers supervised patients in setting behavioural goals such as smaller food portions. At each visit, the goal from the previous was reviewed and relevant praise and/or problem solving to barriers were discussed	Yes: patients and nurse case managers discussed strategies to overcome social barriers such as asking their partner to also cut out unhealthy foods such as ‘ice cream’	No	No	Usual care by their primary care provider	^96^ ^,^ ^97^
Sixta and Ostwald, 2008	Yes: patients were taught about the disease and related complications	No	Yes: patients were taught about blood glucose management	Yes: Patients were taught about disease ‘related emotions’	Yes: patients were taught about healthy behaviours such as the effect of exercise and nutrition. Promotores assisted patients in setting/revising behaviour goals and assisted in follow-up and problem solving	No	Possibly—patients were taught about ‘multidisciplinary team management’	No	Wait list and usual care	
Skelly *et al*., 2009	Yes: patients were taught about disease symptoms and how they relate to diabetes	Yes: patients were taught about insulin/oral medication	Yes: patients were taught symptom management strategies and were given materials on the prevention of symptoms. Patients chose which strategies they wanted to use. Self-care practices taught include home glucose monitoring, foot care and checking urine for ketones if blood sugar was >240	Yes: patients were taught several psychological strategies such as positive self-talk, positive coping strategies and stress reduction—abdominal breathing, visual imagery	Yes: physical activity and diet were addressed for example nurses went with patients to their kitchen to teach them how to read nutrition labels. Patients were given ‘homework’ and set goals at the end of each session	Yes: family members, if present, were invited to sit in during the home visits	Yes: patients were taught when to contact their healthcare provider, for example, to contact their healthcare provider if their readings were frequently >140 before meals	No	A weight and diet program consisting of four modules that addressed Weight Maintenance (two modules), Modifying Fat, and Modifying Sodium in the diet. The modules did not address symptoms directly	^98^
Spencer *et al*., 2018	Yes: participants were taught information about diabetes	No	No	Yes: when patients set goals and identified problems, they were able to discuss the emotional impact of that problem with the CHWs. The curriculum also taught stress-lowering activities	Yes: the curriculum involved culturally appropriate diet and physical activity advice, including exercise videos. CHWs also help participants set goals using the 5-step goal-setting process ad developing and executing an action plan for that goal	Yes: the curriculum emphasises that healthy eating is beneficial for the whole family. A group session provided social support and role-playing support exercises to improve social support and communication with family members about diabetes self-management	Yes: CHWs helped participants improve communication skills with their providers and facilitated necessary referrals to other services. CHWs accompanied participants to one clinic visit with their primary health care provider	No	Enhanced usual care, including a 2 h class conducted by a research assistant covering how to interpret their clinical and anthropometric results	^99^
	Yes: PLs addressed questions about diabetes and its care	No	No	Yes: PLs discussed psychosocial concerns with participants	Yes: using the same 5-step goal process as the CHWs. Group sessions were an opportunity to discuss challenges and problem solve	Yes: PLs helped participants take inventory of support sources	Yes: Pls helped participants in developing strategies to navigate the health care system	No	After the initial 6 months of the main intervention, participants randomized to CHW worker only group received monthly telephone calls from a CHW who had led their DSME group to check in and assess their progress	
Talavera *et al*., 2021	Yes: during medical visits, the medical provider reviewed patients laboratory results with them and their medical history. During the DSME classes, groups discussed diabetes pathophysiology in relation to cultural beliefs	Yes: medical providers and patients collaboratively discussed barriers to medication adherence during the medical visit. Medication adherence was also discussed during DSME classes	Yes: medical providers and patients collaboratively discussed home glucose self-monitoring during the medical visit	Yes: during the behaviour visit, patients collaboratively assessed emotional factors affecting diabetes self-management. The behaviour providers also provided psychoeducation. DSME classes involved discussions on psychosocial well-being (prevention and coping with depression, anxiety, diabetes distress, stress management and problem solving)	Yes: during the behaviour visits, patients created SMART goals and personal action plans. During DSME classes, groups discussed nutrition in the context of the traditional Latin diet and how to incorporate physical activity into everyday life	Yes: during the behaviour visit, patients discussed family barriers. Also, patients were refereed to social work/family services when needed. The DSME curriculum emphasized involving family in self-management and lifestyle activities	Yes: indirectly	Yes: medical visits also involved the development of a treatment plan. Care-coordination involved referrals to other health departments and community resources when needed	Control patients received primary care provider led usual care, with referrals to health education and behavioural health as needed	
Thom *et al.*, 2013	Yes: peer coaches and patients discussed current and target clinical values for HbA1c	Yes: peer coaches facilitated medication understanding and adherence	Yes: peer coaches discussed self-management skills such as using a glucometer and appropriate strategies for hypoglycaemia	Yes: peer coaches provided social and emotional support and helped patients with stress management	Yes: peer coaches assisted with lifestyle changes such as healthy eating and physical activity	Yes: peer coaches provided social support and shared stories about their own lives and families	Yes: peer coaches helped patient to navigate the clinic and could accompany the patient during a clinic visit	Yes: peer coaches provided information on community resources	Usual care, including referrals to a nutritionist and diabetes educator if needed	^100^
Wang *et al.*, 2018	No	Yes: covered in the usual care diabetes education	Yes: the usual care diabetes education covered risk and management of hyperglycaemic and hypoglycaemic situation and blood glucose self-monitoring. Participants also received a Bluetooth enabled glucometer linked to the Diabetes Connect app to track blood glucose	Yes: the lifestyle intervention sessions cover stress management and balancing thoughts	Yes: the lifestyle intervention sessions cover various aspects of exercise and healthy eating and includes a grocery shopping tour. Participants were provided with the LoseIt! App, a pedometer, a food scale and a weight scale to track calories, physical activity and weight	No	No	No	Control group received usual care and diabetes education from their primary care physicians and diabetes educators	
	No	Yes: covered in the usual care diabetes education	Yes: the usual care diabetes education covered risk and management of hyperglycaemic and hypoglycaemic situation and blood glucose self-monitoring. Participants also received a regular glucometer to track blood glucose in a paper diary	Yes: the lifestyle intervention sessions cover stress management and balancing thoughts	Yes: the lifestyle intervention sessions cover various aspects of exercise and healthy eating and includes a grocery shopping tour. Participants were provided with the CalorieKing paper diary, a pedometer, a food scale and a weight scale to track calories, physical activity and weight	No	No	No		
Wayne *et al.*, 2015	No—not explicitly	Yes: medication adherence was a goal emphasized by the health coaches	Yes: participants logged and monitored their blood glucose levels via the app	Yes: coaches emphasized stress management as goal for participants. Participants could also log and track their mood on the app	Yes: coaches guided participants in setting goals and making plans regarding diet (reducing carbohydrate intake) and increasing exercise frequency. Participants could also access free group exercise classes available at the local community health centre. Participants could log food intake and exercise frequency onto the app for the coaches to monitor and provide guidance when they diverged from their goals	No	Yes: a goal emphasized by health coaches was participant communication with primary care physicians and, generally, within the health system	No	Control participants received in-person health coaching only, with no additional mobile support	
Whittemore-2020	Yes: session 1 focused on understanding diabetes	Yes: session 1 highlighted the need to take medication and session 5 focused on going diabetes medications	Yes: participants were given glucometers, test strips and lancets and were taught the need to self-monitor blood glucose and how this relates to carbohydrate intake	Yes: 4 of the sessions included stress management activities	Yes: throughout the sessions and texts/pictures participants were taught strategies to improve their diet such as how to read nutrition labels, menu planning with limited resources, food portion measurement and the ‘plate method’. Also, the benefits and precautions of physical activity were highlighted, and goals were encouraged through texts	Yes: family could be invited to sessions	Yes: session 5 covered how to talk to health care professionals	No		^100^
Aikens *et al*., 2022	Yes: pre-recorded messages contained information on depression symptoms	Yes: pre-recorded messages highlighted the importance of adhering to their anti-depressant regime and advice on how to do so and get refills	Yes: at the beginning of each pre-recorded call, patients answered PHQ-9 items to track their depression symptom severity, which then tailored the advice they were given	Yes	Yes: pre-recorded messages covered lifestyle advice such as physical activity and sleep	Yes: patients had an ‘in-home’ supporter and CP who lived outside the home. They provided social support. At the end of each IVR call, CPs were sent a structured report along with advice on how to support the patient with their depression	Yes: pre-recorded messages included advice on when and how to reach out to their physician	If patients reported suicidal feelings, the system alerted their primary care team	Control patients received enhanced usual care. While both they and their nominated CP received printed materials on depression self-management, they did not receive additional self-management support via the IVR	^101^
Apter *et al*., 2019	Yes: CHWs taught patients how to search for health information online and access educational materials on the portal	Yes: CHWs explained the difference between controller and rescue medications, and proper inhaler use	Yes: CHWs drafted individualized asthma management plans with each patient and taught patients how to mitigate asthma triggers	No	Yes: signposting to relevant community resources such as smoking cessation and housing programs	Yes: CHWs also established relationships with patients’ families	Yes: CHWs taught patients how to chat with doctors via the portal, especially regarding exposure to key allergens and booking appointments	CHWs were involved in care navigation and referrals	Portal training only + usual care	^102^
Krieger *et al*., 2015	Yes: visits covered asthma basics including asthma pathophysiology, when to seek emergency care and important vaccines	Yes: visits covered medication adherence including providing participants with a medication box; problem solving concerns about side effects, cost, access and getting to pharmacy; and CHWs assessing participants’ understanding of when to use controller medications	Yes: visits covered symptom management including peak flow monitoring—participants were given a peak flow monitor and diary and taught the correct technique; getting help during an asthma attack- CHWs demonstrated ‘Belly Breathing’ and other relaxation techniques	Yes: visits covered stress management	Yes: visits covered environmental control of the home and a cleaning checklist. Participants were given a vacuum, vacuum bag and cleaning kit	Yes: CHWs could engage family support during visits	Yes: visits covered working with the healthcare system including communication strategies, pointers and roleplay. If needed, CHWs could accompany participants to a medical appointment to act as a ‘cultural translator’	CHWS made referrals to community resources for childcare, food, employment and citizenship assistance. CHWs also faxed visit details to the clinics for feedback	Usual care, information on community resources for asthma self-management and educational pamphlets. At the end of the study, participants in the control group received a home visit by a CHW and the intervention group resources	
Martin *et al*., 2009	Yes: group session 1 covered asthma anatomy and physiology and understanding physiologic reactions to stressors. Home visit 3 covered asthma triggers	Yes: home visit 1 covered controller medications, spacers, inhalers	Yes: home visit 2 covered symptom recognition and management	Yes: group session 2 covered sociocultural definitions of stress; effects of stress on asthma management and an action plan to improvement ability to manage stress	Yes: groups session 3 and 4 and home visit 5 covered benefits of physical activity an action plan for physical activity. Home visit 4 covered smoking cessation and tobacco smoke avoidance	Yes: group session 4 covered discussions on current positive social support. All home visits also covered social support	Yes: home visit 2 covered communicating with providers. CHWs encouraged proactive communications between patients and their health care providers	No	2 mailings covering the asthma education information presented at the group sessions for the intervention and a US$30 cheque	
Young *et al*., 2012	No	Yes: pharmacists followed the communication guide to identity and address patient barriers to medication adherence. Pharmacists also used a series of questions to assess whether patients required additional education regarding inhaler technique	No	No	No	No	No	Pharmacists review their electronic health records. If they identified severe asthma related problems, they referred patients to their primary healthcare provider	Usual care, including mail receipt of a prescription refill with written instructions on medication use	
Evans-Hudnall *et al*., 2014	Yes: Patients were given a detailed workbook on the signs and symptoms of stroke and risk factors for primary and secondary stroke	No	Yes: patients were given a detailed workbook on the signs and symptoms of stroke	Yes: patients were taught cognitive reframing techniques to minimize negative thoughts concerning their ability to change lifestyle habits. Also, they were taught deep breathing and guided imagery skills to help identify and decrease their stress levels	Yes: patients were given a dietary and exercise tracking form. Patients were asked to identify lifestyle habits that increased their risk for secondary stroke and how to assess change in these habits, including potential barriers and problem solving. Patients were taught stimulus control—removing environmental factors associated with unhealthy habits. Diet and exercise advice was tailored to each patient	Yes: patients were encouraged to engage friends and family members as a source of support to achieve their goals. They were also encouraged to set goals that focused on changing family lifestyle behaviours rather than individual change	No	The health educator facilitated phone calls to aid the patient in getting access to community resources	Usual care	
Kronish *et al*., 2014	Yes: the workshop covered the biology of stroke and stroke treatments	Yes: the workshop stressed the importance of adherence to preventive medications to reduce stroke recurrence and provided suggestions for optimizing medication adherence	Yes: the workshop covered key symptom management related to blood pressure and cholesterol	No	No	Yes: participants could bring a family member, friend or home attendant to the workshops	Yes: the workshop covered working with your health care team, including communication	Participants were also given a list of local health providers, including those that accepted patients without health insurance	Usual care and wait list. Control participants received the workshop after a 1-year waiting period	^103^
Tiliakos *et al*., 2013	Yes: class content included an overview of arthritis pathophysiology	Yes: class content included an overview of arthritis medications	Yes: class content covered appropriate use of injured joints	Yes: class content involved individualized relaxation programs	Yes: class content involved the development of individualized exercise programs	No	Yes: class content covered aspects of patient–physician communication	No	Usual care	^104^
Eakin *et al*., 2007	No	No	No	No	Yes: patients set a self-management goal related to physical activity or healthy eating, and identified one or two types of social environmental resources they could use to help them reach their goal. Patients received a goal sheet that summarized their action plan. Phone calls addressed problem solving. Also, tailored newsletters reinforced these goals	Yes: family and friends were included as potential social-environmental resources	No	NA	Control patients were mailed a local area community resources guide and three newsletters on basic financial management	
Kangovi *et al*., 2017	Yes: if participants wanted further disease education, CHWs navigated them to the appropriate clinician	Yes: the tailored action plans could include strategies for medication adherence	Yes: the tailored action plans could include strategies for symptom management such as blood glucose and blood pressure monitoring	Yes: the support groups discussed psychosocial stressors	Yes: the tailored action plans could include strategies for lifestyle changes such as increased physical activity, healthy eating and quitting smoking. CHW provided support such as food pantry visits with participants	Yes: the taction plans could involve strategies for involving family in the participants’ goal. Also, the support discussed relationships with friends and family members	Yes: the action plans could involve discussion pointers to bring up with the participant’s primary care provider	CHW also navigated participants towards appropriate community resources and sent progress reports to the participants primary care team	One time collaborate goal setting, followed by usual care	^105^ ^,^ ^106^
Kennedy *et al*., 2013	NP	NP	NP	NP	NP	NP	NP	No	Wait list control	^107^
McKee *et al*., 2011	NP	NP	Yes: Patients were leased Cardiocom telemonitoring equipment to send their daily self-monitored blood pressure and glucose readings. Results were transmitted to the program manager and formatted as weekly reports. The reports were sent to the primary care provider via secure clinical email for review and treatment modification if necessary	NP	NP	NP	NP	Primary care providers used weekly report to modify treatment plans	Usual care	
Mercer *et al*., 2016	NP	NP	NP	Yes: mindfulness-based stress management CDs	NP	NP	NP	Practitioners were encouraged to link patients with relevant local resources and community services when appropriate	Usual care	^108^
Riley *et al*., 2001	Yes: CIRS covers whether or not the participant has access to information about the condition	Yes: CIRS cover medication taking as a behaviour	No—not explicitly	No	Yes: CIRS covers behavioural targets such as eating more fruits and vegetables, getting more physical activity and quitting smoking	Yes: CIRS involves questions arounds family and friend support, e.g. ‘Have your family or friends exercised with you?’	Yes: CIRS covers questions around support/communication with the participants’ healthcare team, e.g. ‘Has your doctor or other health care provider listened carefully to what you had to say about your illness?’	No	Wait list control: received the intervention 1 month after the intervention group	
Swerissen *et al*., 2006	No—not explicitly	No—not explicitly	Yes: the program manual covers symptom management	Yes: the program manual covers dealing with the emotions of chronic illness (e.g. anger and depression) and relaxation techniques	Yes: program covers exercise and healthy eating. There was weekly action planning and feedback on progress. Also, there was modelling of self-management behaviours and problem-solving strategies	Yes: program covers communication skills with friends and family	Yes: program covers communication skills with health care providers. The program emphasizes the critical role of the patient managing their own health in partnership with health professionals	No	Wait list control—participants received the intervention 6 months later	
Willard-grace-2015	Yes: the health coach assesses the patient’s knowledge about HbA1c, systolic blood pressure (SBP) or low-density lipoprotein (LDL). They discuss the patient’s most recent results for these measures, their goal for these numbers and how to reach the goal	Yes: during the pre-visit, health coaches go through ‘medication reconciliation’ with the patient, which includes reviewing the medications under prescription, assessing patients knowledge of the purpose of the medications and identifying barriers to adherence	No—not mentioned explicitly	Yes: during the ‘post visit’, health coaches negotiate an ‘action plan’ with the patient, which includes strategies to reduce stress	Yes: the ‘action plan’ also addresses diet, exercise and other relevant lifestyle factors. The telephone follow-ups address barriers and problems with meetings these goals	No	Yes: during the medical visit the health coach acts as an advocate for the patient, helping them to remember questions or concerns raised during the pre-visit and praising the patients, relaying to the clinician steps the patient has taken to care for their health	Health coaches were also responsible for further referrals to specialists and resource navigation	Usual care, including access to clinic resources that would normally be available such as visits with a clinician, diabetes educator, nutritionist, chronic care nurse and educational classes	^109^ ^,^ ^110^

### Risk of bias

**Fig. 2:**
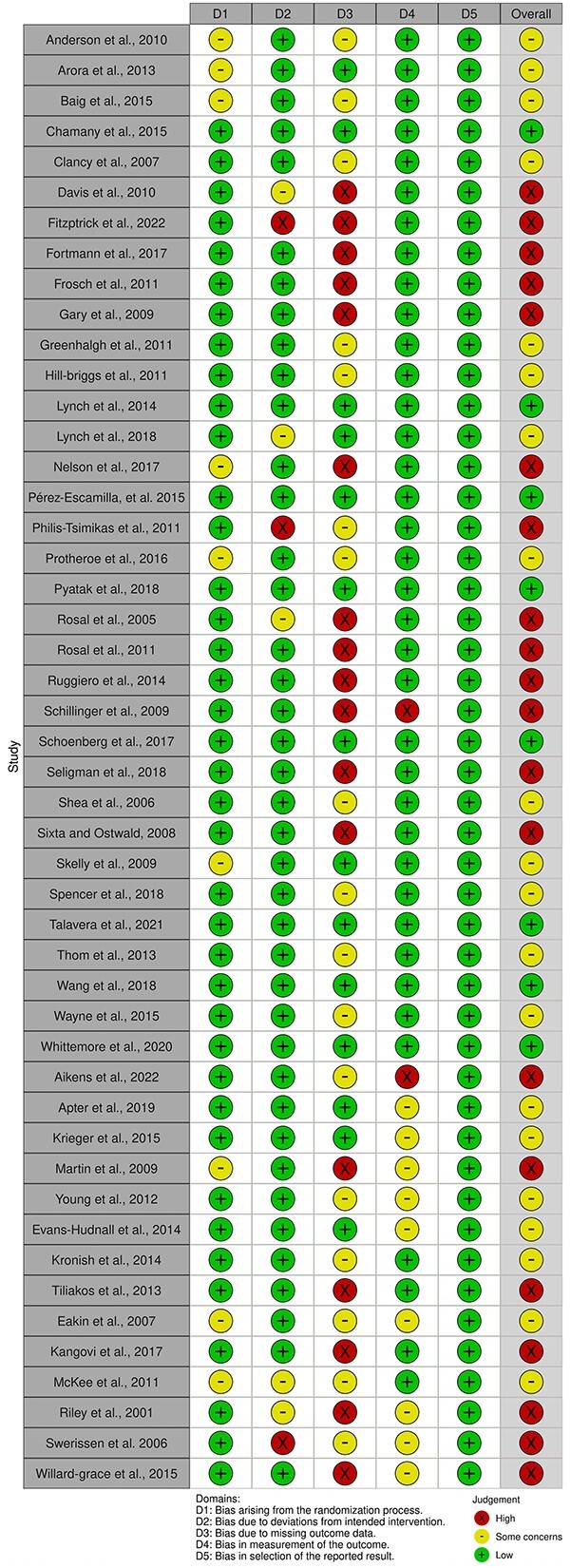
Risk of bias in randomized trials (RoB2).

**Fig. 3:**
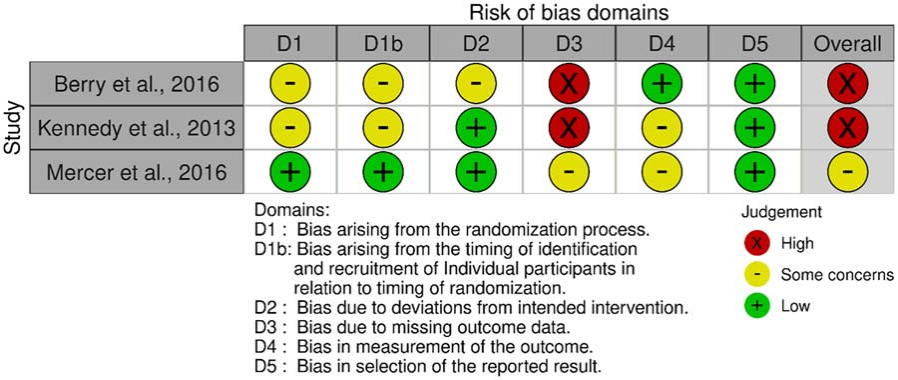
Risk of bias for cluster-RCTs (RoB2 CRT).

In 43 studies, there was a substantial risk of bias, mainly due to loss of follow-up (Figs. 2 and 3). For continuous outcomes, most studies had insufficient outcome data. Furthermore, there were sometimes large differences in the proportion of dropouts between the intervention and control groups. Following Cochrane’s RoB2 guidance, we did not assume that multiple imputation or ‘last observation carried forward’ corrected for bias due to missing outcome data, unless there was a sensitivity analysis showing that there was no relationship between missingness of data and its true value.

After randomization, some studies had significant differences in key outcome variables and prognostic factors between the intervention and control, which was concerning. In the Cluster-RCTs, one study randomized clusters before individual participant recruitment, which was also a cause for concern. Due to the nature of self-management interventions, blinding to intervention status for participants was not possible for any of the studies.

For the diabetes studies, the outcome assessors were usually blinded, reducing risk of bias. For the study outcomes that were self-reported, these were deemed to be at risk of social desirability bias.

### Synthesis

#### All studies

Overall, 22 intervention arms had a positive effect on their primary outcome compared to the control arms, while 27 did not. Three studies did not provide this information. Interventions delivered remotely and exclusively to individuals had a higher proportion of positive outcome results. There was no strong trend in the proportion of positive outcomes when comparing interventions according to specific SES tailoring, use of CHWs in intervention delivery, the number of self-management components used, use of a named theory, risk of bias or disease focus. Supplementary Material 2 enumerates the primary outcome results according to key study characteristics, as a means of exploring what the active intervention components for socio-economically deprived populations might look like.

### Diabetes

**Fig. 4:**
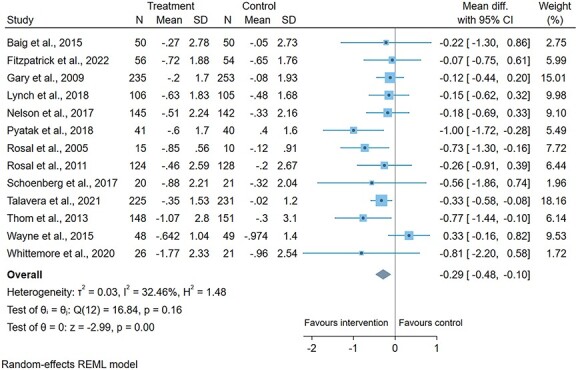
Forest plot for diabetes self-management RCTs.

The main outcome for diabetes studies was mean change in haemoglobin A1c (HbA1c). HbA1c is a biomarker which measures the level of glycated haemoglobin in the bloodstream. Achieving a lower level of HbA1c is associated with reductions in diabetes-related complications, reflecting improved self-management.^27^ For the 13 studies included in the random-effects meta-analysis using this outcome, we imputed the SD values for five. Figure 4 shows the forest plot. Improvements in diabetes control (i.e. reductions in HbA1c) favoured the intervention groups. The pooled mean difference shows that participants in the intervention groups had a 0.29% greater reduction in HbA1c than the control group participants. (95% CIs: −0.48 to −0.10). The Cochrane *Q* statistic (*Q* (12) = 16.84) indicated that the studies were homogenous (*P* = 0.16). This was reinforced by the *I*^2^ value of 32.46%, which suggests only moderate heterogeneity (25% > *I*^2^ > 50%).

### Publication bias

For the diabetes meta-analysis, there was not strong evidence of publication bias. Details on publication bias can be found in the Supplementary Material 3.

## GRADE

Our certainty in the body of evidence for diabetes is moderate (Supplementary Material 4). The level of certainty was downgraded mainly due to risk of methodological bias, specifically, insufficient outcome data.

## Discussion

### Main finding of this study

For the diabetes meta-analysis (*n* = 13), the intervention groups had a 0.29% greater reduction in HbA1c than the control groups. This is similar to the results of another meta-analysis of diabetes self-management education programs in American ethnic minority groups.^78^ Some suggest that a HbA1c reduction of at least 0.50% is needed for clinical significance.^79^ Therefore, while the meta-analysis indicates that diabetes self-management interventions were more successful than the control treatment, the clinical improvements were modest at best. Overall, the quality of the body of evidence for diabetes self-management programs, as qualified by GRADE, was moderate.

Narratively, findings were mixed for multi-morbidity and other individual conditions. Across all studies, we tabulated and compared the proportion of positive results according to key intervention characteristics. There was a slightly higher proportion of interventions with positive results that were delivered remotely and exclusively to individuals compared to other delivery modes. While this may be a chance finding, it is in line with the results of a previous review of self-management support interventions.^12^ Interventions with a reported theoretical basis had a similar proportion of positive results compared to interventions with no reported or explicit theoretical basis. Few studies indicated why their chosen theory was the most appropriate for the intervention or provided evidence of mapping out the theoretical constructs onto the intervention components.

There was no apparent trend in the proportion of positive results when stratified by the number of self-management components. This is in line with the results of another review.^12^ Less than half the interventions in this review had fidelity checks and often the intervention descriptions were brief. A review of fidelity and engagement in self-management interventions concluded the need for proper fidelity assessments to determine which components are contributing towards the intervention effect or lack of effect.^80^

### What is already known on this topic

Self-management interventions can improve clinical outcomes and reduce health service utilization in the general population. However, previous reviews and studies have shown that individuals from socioeconomically deprived backgrounds are less likely to reap the benefits of these interventions due to a number of factors such as lower intervention engagement.^5^^,^^6^^,^^8–12^

### What this study adds

This systematic review is the first to synthesize evidence on the effectiveness of self-management interventions for long-term conditions in people experiencing socio-economic deprivation and the specific tailoring and components of these interventions. The main strength of this review is the comprehensiveness of the search strategy, both in the number of databases searched and the breadth of the keyword search. We expanded on terms for socio-economic status and social determinants to include synonyms for proxy measurements unlike previous reviews on this topic.^8^ We included disease-specific searches rather than solely using general terms for ‘long term conditions’. We also described the interventions in accordance with TiDier guidelines to aid future intervention replication and testing.

In light of the results of this review, we recommend that future intervention development should clearly state their theoretical basis. It is increasingly being recognized that public health interventions based on behaviour change theory are more likely to be effective than those lacking a theoretical basis.^81^ In addition, future interventions could incorporate the common socioeconomic tailoring methods identified in this review. However, adaptations beyond addressing language and literacy barriers are needed. Evaluations should explore which self-management components are most effective (not just most common) in interventions targeted at people experiencing socio-economic deprivation, potentially using factorial design methods. More trials based outside the USA and addressing long-term conditions other than diabetes are needed. Whilst it is important to address diabetes, this population has a range of LTCs, and research should focus on managing these as well as the complex interplay of having two or more LTCs (multimorbidity). Socio-economic deprivation is associated with a higher incidence of multimorbidity over a 15-year period.^82^ In addition, deprivation is associated with a higher prevalence of common LTCs, including diabetes, but also anxiety, depression, dyspepsia and coronary heart disease.^83^

### Limitations of this study

The overall methodological quality of the studies included was poor. Although the Egger test for the meta-analysis indicated no publication bias, we limited our selection criteria to only include peer-reviewed articles available in English. There may have been relevant non-English or grey literature evidence not included in this review. Finally, meta-analysis was not possible for all the studies due to the large variation in outcomes. Therefore, we tabulated the results. However, this method does not account for the magnitude of the effect sizes or differences in sample size.

## Conclusion

Self-management interventions of diabetes tailored for people experiencing socio-economic deprivation produces clinically modest but statistically significant reductions in HbA1c, which is promising. Narratively, other studies on multi-morbidity and other individual LTCs had mixed findings, and more evidence is needed. Self-management interventions in the general population with LTCs have previously been found less effective for people experiencing socio-economic deprivation, and this review highlights the importance of tailored, inclusive interventions in this population. Tailoring included adaptions for low literacy (e.g. visual aids), the involvement of community health workers or peer leaders, providing helpful materials if needed (e.g. mobile phone) and financial incentives, but more strategies could be developed. In terms of the self-management components of the interventions, our evidence suggests the number included may not be important, and other factors may matter more, such as the quality of each component. Self-management strategies and interventions are becoming an increasingly popular approach to LTC management; to avoid exacerbating health inequalities, these interventions should include adaptions for people experiencing socio-economic deprivation.

## Conflict of interest

The authors have no conflict of interest to disclose.

## Funding

The National Institute for Health and Care Research (NIHR) School for Primary Care Research (project reference 539). The views expressed are those of the author(s) and not necessarily those of the NIHR or the Department of Health and Social Care.

## Data availability

Data is available on reasonable request from Megan Armstrong, the principal investigator.

## Supplementary Material

Supp_1_fdad145

Supp_2_fdad145

supp_3_fdad145

supp_4_fdad145

SM_review-appendix_fdad145

## Notes

**Tosan Okpako,** PhD Student

**Abi Woodward,** Research Fellow

**Kate Walters,** Professors

**Nathan Davies,** Associate Professor

**Fiona Stevenson,** Professors

**Danielle Nimmons,** GP and PHD Student

**Carolyn A. Chew-Graham,** Professors

**Joanne Protheroe,** Professors

**Megan Armstrong,** Lecturer

## References

[1] Department of Health . Long-Term Conditions Compendium of Information; 2012. https://assets.publishing.service.gov.uk/government/uploads/system/uploads/attachment_data/file/216528/dh_134486.pdf (28 February 2023, date last accessed).

[2] NHS Digital . Health Survey for England—NHS Digital. Published 2019. https://digital.nhs.uk/data-and-information/publications/statistical/health-survey-for-england&gt; (11 January 2021, date last accessed).

[3] Katikireddi SV, Skivington K, Leyland AH et al. The contribution of risk factors to socioeconomic inequalities in multimorbidity across the lifecourse: a longitudinal analysis of the Twenty-07 cohort. BMC Med 2017;15(1):152. 10.1186/s12916-017-0913-6. 28835246 PMC5569487

[4] NHS Choices . House of Care—A Framework for Long Term Condition Care. Published 2022. https://www.england.nhs.uk/ourwork/clinical-policy/ltc/house-of-care/ (14 November 2022, date last accessed).

[5] Allegrante JP, Wells MT, Peterson JC. Interventions to support behavioral self-management of chronic diseases. Annu Rev Public Health 2019;40:127–46. 10.1146/annurev-publhealth. 30601717 PMC6684026

[6] Panagioti M, Richardson G, Small N et al. Self-management support interventions to reduce health care utilisation without compromising outcomes: a systematic review and meta-analysis. BMC Health Serv Res 2014;14(1):1–4. 10.1186/1472-6963-14-356. PMC4177163 25164529

[7] Barlow J, Wright C, Sheasby J et al. Self-management approaches for people with chronic conditions: a review. Patient Educ Couns 2002;48:177–87. 12401421 10.1016/s0738-3991(02)00032-0

[8] Hardman R, Begg S, Spelten E. What impact do chronic disease self-management support interventions have on health inequity gaps related to socioeconomic status: a systematic review. BMC Health Serv Res 2020;20(1):1–15. 10.1186/s12913-020-5010-4. PMC7045733 32106889

[9] Maitra S . Can patient self-management explain the health gradient? Goldman and Smith’s “Can patient self-management help explain the SES health gradient?” (2002) revisited. Soc Sci Med 2010;70(6):802–12. 10.1016/j.socscimed.2009.08.043. 20117869

[10] Protheroe J, Brooks H, Chew-Graham C et al. ‘Permission to participate?’ A qualitative study of participation in patients from differing socio-economic backgrounds. J Health Psychol 2013;18(8):1046–55. 10.1177/1359105312459876. 23104997

[11] Schaffler J, Leung K, Tremblay S et al. The effectiveness of self-management interventions for individuals with low health literacy and/or low income: a descriptive systematic review. J Gen Intern Med 2018;33(4):510–23. 10.1007/s11606-017-4265-x. 29427178 PMC5880764

[12] Van Hecke A, Heinen M, Fernández-Ortega P et al. Systematic literature review on effectiveness of self-management support interventions in patients with chronic conditions and low socio-economic status. J Adv Nurs 2017;73(4):775–93. 10.1111/jan.13159. 27653960

[13] Galobardes B, Shaw M, Lawlor DA et al. Indicators of socioeconomic position (part 1). J Epidemiol Community Health (1978) 2006;60(1):7–12. 10.1136/jech.2004.023531. PMC2465546 16361448

[14] Woodward A, Davies N, Walters K et al. Self-management of multiple long-term conditions: a systematic review of the barriers and facilitators amongst people experiencing socioeconomic deprivation. PloS One 2023;18(2):e0282036. 10.1371/journal.pone.0282036. 36809286 PMC9942951

[15] Basto-Abreu A, Barrientos-Gutierrez T, Wade AN et al. Multimorbidity matters in low and middle-income countries. J Multimorb Comorb 2022;12:263355652211060. 10.1177/26335565221106074. PMC9208045 35734547

[16] Sterne JAC, Savović J, Page MJ et al. RoB 2: a revised tool for assessing risk of bias in randomised trials. BMJ Published online 28 August 2019;l4898. 31462531 10.1136/bmj.l4898

[17] Higgins JP, Altman DG, Gotzsche PC et al. The Cochrane Collaboration's tool for assessing risk of bias in randomised trials. BMJ 2011, 343. 10.1136/bmj.d5928 PMC3196245 22008217

[18] Hoffmann TC, Glasziou PP, Boutron I et al. Better reporting of interventions: template for intervention description and replication (TIDieR) checklist and guide. BMJ 2014;348(Mar07 3):g1687–7. 10.1136/bmj.g1687. 24609605

[19] Higgins J, Li T, Deeks J. Chapter 6: choosing effect measures and computing estimates of effect. In: Higgins J, Thomas J, Chandler J et al. (eds). Cochrane Handbook for Systematic Reviews of Interventions version 6.3. Wiley-Blackwell, New Jersey, USA, 2022.

[20] von Hippel PT . The heterogeneity statistic I2 can be biased in small meta-analyses. BMC Med Res Methodol 2015;15(1):35. 10.1186/s12874-015-0024-z. 25880989 PMC4410499

[21] Meader N, King K, Llewellyn A et al. A checklist designed to aid consistency and reproducibility of GRADE assessments: development and pilot validation. Syst Rev 2014;3(1)1–9. 10.1186/2046-4053-3-82. PMC4124503 25056145

[22] Guyatt GH, Oxman AD, Montori V et al. GRADE guidelines: 5. Rating the quality of evidence—publication bias. J Clin Epidemiol 2011;64(12):1277–82. 10.1016/j.jclinepi.2011.01.011. 21802904

[23] Guyatt GH, Oxman AD, Vist G et al. GRADE guidelines: 4. Rating the quality of evidence—study limitations (risk of bias). J Clin Epidemiol 2011;64(4):407–15. 10.1016/j.jclinepi.2010.07.017. 21247734

[24] Guyatt GH, Oxman AD, Kunz R et al. GRADE guidelines 6. Rating the quality of evidence—imprecision. J Clin Epidemiol 2011;64(12):1283–93. 10.1016/j.jclinepi.2011.01.012. 21839614

[25] Guyatt GH, Oxman AD, Kunz R et al. GRADE guidelines: 7. Rating the quality of evidence—inconsistency. J Clin Epidemiol 2011;64(12):1294–302. 10.1016/j.jclinepi.2011.03.017. 21803546

[26] Guyatt GH, Oxman AD, Kunz R et al. GRADE guidelines: 8. Rating the quality of evidence—indirectness. J Clin Epidemiol 2011;64(12):1303–10. 10.1016/j.jclinepi.2011.04.014. 21802903

[27] Anderson DR, Christison-Lagay J, Villagra V et al. Managing the space between visits: a randomized trial of disease management for diabetes in a community health center. J Gen Intern Med 2010;25(10):1116–22. 10.1007/s11606-010-1419-5. 20556536 PMC2955486

[28] Arora S, Peters AL, Burner E et al. Trial to examine text message-based mhealth in emergency department patients with diabetes (TExT-MED): a randomized controlled trial. Ann Emerg Med 2013;63(6):745–54. 10.1016/j.annemergmed.2013.10.012. 24225332

[29] Baig AA, Benitez A, Locklin CA et al. Picture good health: a church-based self-management intervention among Latino adults with diabetes. J Gen Intern Med 2015;30(10):1481–90. 10.1007/s11606-015-3339-x. 25920468 PMC4579235

[30] Berry DC, Williams W, Hall EG et al. Imbedding interdisciplinary diabetes group visits into a community-based medical setting. Diabetes Educ 2016;42(1):96–107. 10.1177/0145721715620022. 26647415

[31] Chamany S, Walker EA, Schechter CB et al. Telephone intervention to improve diabetes control: a randomized trial in the New York City A1c registry. Am J Prev Med 2015;49(6):832–41. 10.1016/j.amepre.2015.04.016. 26232903 PMC4656092

[32] Clancy DE, Huang P, Okonofua E et al. Group visits: promoting adherence to diabetes guidelines. J Gen Intern Med 2007;22(5):620–4. 10.1007/s11606-007-0150-3. 17443369 PMC1852919

[33] Davis RM, Hitch AD, Salaam MM et al. TeleHealth improves diabetes self-management in an underserved community: diabetes TeleCare. Diabetes Care 2010;33(8):1712–7. 10.2337/dc09-1919. 20484125 PMC2909047

[34] Fitzpatrick SL, Papajorgji-Taylor D, Schneider JL et al. Bridge to health/Puente a la Salud: a pilot randomized trial to address diabetes self-management and social needs among high-risk patients. Transl Behav Med 2022;12(7):783–92. 10.1093/tbm/ibac016. 35849138 PMC9291384

[35] Fortmann AL, Gallo LC, Garcia MI et al. Dulce digital: an mHealth SMS based intervention improves glycemic control in Hispanics with type 2 diabetes. Diabetes Care 2017;40(10):1349–55. 10.2337/dc17-0230. 28600309 PMC5606313

[36] Frosch DL, Uy V, Ochoa S et al. Evaluation of a behavior support intervention for patients with poorly controlled diabetes. Arch Intern Med 2011;171(22):2011–7. 10.1001/archinternmed.2011.497. 21986347

[37] Gary TL, Batts-Turner M, Yeh HC et al. The effects of a nurse case manager and a community health worker team on diabetic control, emergency department visits, and hospitalizations among urban African Americans with type 2 diabetes mellitus. Arch Intern Med 2009;169(19):1788–94. 10.1001/archinternmed.2009.338. 19858437 PMC5675128

[38] Greenhalgh T, Campbell-Richards D, Vijayaraghavan S et al. New models of self-management education for minority ethnic groups: pilot randomized trial of a story-sharing intervention. J Health Serv Res Policy 2011;16(1):28–36. 10.1258/jhsrp.2010.009159. 20739577

[39] Hill-Briggs F, Lazo M, Peyrot M et al. Effect of problem-solving-based diabetes self-management training on diabetes control in a low income patient sample. J Gen Intern Med 2011;26(9):972–8. 10.1007/s11606-011-1689-6. 21445680 PMC3157525

[40] Lynch EB, Liebman R, Ventrelle J et al. A self-management intervention for African Americans with comorbid diabetes and hypertension: a pilot randomized controlled trial. Prev Chronic Dis 2014;11:130349. 10.5888/pcd11.130349. PMC4040140 24874782

[41] Lynch EB, Mack L, Avery E et al. Randomized trial of a lifestyle intervention for urban low-income African Americans with type 2 diabetes. J Gen Intern Med 2018;34(7):1174–83. 10.1007/s11606-019-04894-y. PMC6614233 30963440

[42] Nelson K, Taylor L, Silverman J et al. Randomized controlled trial of a community health worker self-management support intervention among low-income adults with diabetes, Seattle, Washington, 2010-2014. Prev Chronic Dis 2017;14(2). 10.5888/pcd14.160344. PMC5303652 28182863

[43] Pérez-Escamilla R, Damio G, Chhabra J et al. Impact of a community health workers-led structured program on blood glucose control among Latinos with type 2 diabetes: the DIALBEST trial. Diabetes Care 2015;38(2):197–205. 10.2337/dc14-0327. 25125508 PMC4302259

[44] Philis-Tsimikas A, Fortmann A, Lleva-Ocana L et al. Peer-led diabetes education programs in high-risk Mexican Americans improve glycemic control compared with standard approaches: a Project Dulce promotora randomized trial. Diabetes Care 2011;34(9):1926–31. 10.2337/dc10-2081. 21775748 PMC3161298

[45] Protheroe J, Rathod T, Bartlam B et al. The feasibility of health trainer improved patient self-management in patients with low health literacy and poorly controlled diabetes: a pilot randomised controlled trial. J Diabetes Res 2016;2016:1–11. 10.1155/2016/6903245. PMC5090087 27833922

[46] Pyatak EA, Carandang K, Vigen CLP et al. Occupational therapy intervention improves glycemic control and quality of life among young adults with diabetes: the resilient, empowered, active living with diabetes (REAL diabetes) randomized controlled trial. Diabetes Care 2018;41(4):696–704. 10.2337/dc17-1634. 29351961 PMC5860833

[47] Rosal MC, Olendzki B, Reed GW et al. Diabetes self-management among low-income Spanish-speaking patients: a pilot study. Ann Behav Med 2005;29(3):225–35. 10.1207/s15324796abm2903_9 15946117

[48] Rosal MC, Ockene IS, Restrepo A et al. Randomized trial of a literacy-sensitive, culturally tailored diabetes self-management intervention for low-income Latinos: Latinos en control. Diabetes Care 2011;34(4):838–44. 10.2337/dc10-1981. 21378213 PMC3064037

[49] Ruggiero L, Riley BB, Hernandez R et al. Medical assistant coaching to support diabetes self-care among low-income racial/ethnic minority populations: randomized controlled trial. West J Nurs Res 2014;36(9):1052–73. 10.1177/0193945914522862. 24569698 PMC4215797

[50] Schillinger D, Handley M, Wang F et al. Effects of self-management support on structure, process, and outcomes among vulnerable patients with diabetes. Diabetes Care 2009;32(4):559–66. 10.2337/dc08-0787. 19131469 PMC2660485

[51] Schoenberg NE, Ciciurkaite G, Greenwood MK. Community to clinic navigation to improve diabetes outcomes. Prev Med Rep 2017;5:75–81. 10.1016/j.pmedr.2016.11.015. 27957410 PMC5149068

[52] Seligman HK, Smith M, Rosenmoss S et al. Comprehensive diabetes self-management support from food banks: a randomized controlled trial. Am J Public Health 2018;108(9):1227–34. 10.2105/AJPH.2018.304528. 30024798 PMC6085038

[53] Shea S, Weinstock RS, Starren J et al. A randomized trial comparing telemedicine case management with usual care in older, ethnically diverse, medically underserved patients with diabetes mellitus. J Am Med Inform Assoc 2006;13(1):40–51. 10.1197/jamia.M1917. 16221935 PMC1380195

[54] Sixta CS, Ostwald S. Texas-Mexico border intervention by promotores for patients with type 2 diabetes. Diabetes Educator 2008;34(2):299–309. 10.1177/0145721708314490. 18375779

[55] Skelly AH, Carlson J, Leeman J et al. Controlled trial of nursing interventions to improve health outcomes of older African American women with type 2 diabetes. Nurs Res 2009;58(6):410–8. 10.1097/NNR.0b013e3181bee597. 19851122 PMC2903837

[56] Spencer MS, Kieffer EC, Sinco B et al. Outcomes at 18 months from a community health worker and peer leader diabetes self-management program for Latino adults. Diabetes Care 2018;41 American Diabetes Association Inc:1414–22. 29703724 10.2337/dc17-0978 PMC6014532

[57] Talavera GA, Castañeda SF, Mendoza PM et al. Latinos understanding the need for adherence in diabetes (LUNA-D): a randomized controlled trial of an integrated team-based care intervention among Latinos with diabetes. Transl Behav Med 2021;11(9):1665–75. 10.1093/tbm/ibab052. 34057186 PMC8442567

[58] Thom DH, Ghorob A, Hessler D et al. Impact of peer health coaching on glycemic control in low-income patients with diabetes: a randomized controlled trial. Ann Fam Med 2013;11(2):137–44. 10.1370/afm.1443. 23508600 PMC3601392

[59] Wang J, Cai C, Padhye N et al. A behavioral lifestyle intervention enhanced with multiple-behavior self-monitoring using mobile and connected tools for underserved individuals with type 2 diabetes and comorbid overweight or obesity: pilot comparative effectiveness trial. JMIR Mhealth Uhealth 2018;6(4):e4478. 10.2196/mhealth.4478. PMC5915674 29636320

[60] Wayne N, Perez DF, Kaplan DM et al. Health coaching reduces hba1c in type 2 diabetic patients from a lower-socioeconomic status community: a randomized controlled trial. J Med Internet Res 2015;17(10):e224. 10.2196/jmir.4871. PMC4642794 26441467

[61] Whittemore R, Vilar-Compte M, Burrola-Méndez S et al. Development of a diabetes self-management + mHealth program: tailoring the intervention for a pilot study in a low-income setting in Mexico. Pilot Feasibility Stud 2020;6(1):25. 10.1186/s40814-020-0558-7. 32082611 PMC7023698

[62] Aikens JE, Valenstein M, Plegue MA et al. Technology-facilitated depression self-management linked with lay supporters and primary care clinics: randomized controlled trial in a low-income sample. Telemed E-Health 2022;28(3):399–406. 10.1089/tmj.2021.0042. PMC8968843 34086485

[63] Apter AJ, Localio AR, Morales KH et al. Home visits for uncontrolled asthma among low-income adults with patient portal access. J Allergy Clin Immunol 2019;144(3):846–853.e11. 10.1016/j.jaci.2019.05.030. 31181221 PMC6742549

[64] Krieger J, Song L, Philby M. Community health worker home visits for adults with uncontrolled asthma: the HomeBASE trial randomized clinical trial. JAMA Intern Med 2015;175(1):109–17. 10.1001/jamainternmed.2014.6353. 25419871

[65] Martin MA, Catrambone CD, Kee RA et al. Improving asthma self-efficacy: developing and testing a pilot community-based asthma intervention for African American adults. J Allergy Clin Immunol 2009;123(1):153–9. 10.1016/j.jaci.2008.10.057. PMC2675162 19130936

[66] Young HN, Havican SN, Griesbach S et al. Patient and phaRmacist telephonic encounters (PARTE) in an underserved rural patient population with asthma: results of a pilot study. Telemed E-Health 2012;18(6):427–33. 10.1089/tmj.2011.0194. PMC3399112 22656403

[67] Evans-Hudnall GL, Stanley MA, Clark AN et al. Improving secondary stroke self-care among underserved ethnic minority individuals: a randomized clinical trial of a pilot intervention. J Behav Med 2014;37(2):196–204. 10.1007/s10865-012-9469-2. 23225167

[68] Kronish IM, Goldfinger JZ, Negron R et al. Effect of peer education on stroke prevention: the prevent recurrence of all inner-city strokes through education randomized controlled trial. Stroke 2014;45(11):3330–6. 10.1161/STROKEAHA.114.006623. 25248910 PMC4213208

[69] Tiliakos A, Conn DL, Pan Y et al. The effect of the arthritis self-management program on outcome in African Americans with rheumatoid arthritis served by a public hospital. Clin Rheumatol 2013;32(1):49–59. 10.1007/s10067-012-2090-5. 23053684

[70] Eakin EG, Bull SS, Riley KM et al. Resources for health: a primary-care-based diet and physical activity intervention targeting urban Latinos with multiple chronic conditions. Health Psychol 2007;26(4):392–400. 10.1037/0278-6133.26.4.392. 17605558

[71] Kangovi S, Mitra N, Grande D et al. Community health worker support for disadvantaged patients with multiple chronic diseases: a randomized clinical trial. Am J Public Health 2017;107(10):1660–7. 10.2105/AJPH.2017.303985. 28817334 PMC5607679

[72] Kennedy A, Bower P, Reeves D et al. Implementation of self management support for long term conditions in routine primary care settings: cluster randomised controlled trial. BMJ (Online) 2013;346(7913). 10.1136/bmj.f2882. PMC3652644 23670660

[73] Mckee MD, Fletcher J, Sigal I et al. A collaborative approach to control hypertension in diabetes: outcomes of a pilot intervention. J Prim Care Community Health 2011;2(3):148–52. 10.1177/2150131911401028. 23804793 PMC4580326

[74] Mercer SW, Fitzpatrick B, Guthrie B et al. The CARE plus study—a whole-system intervention to improve quality of life of primary care patients with multimorbidity in areas of high socioeconomic deprivation: exploratory cluster randomised controlled trial and cost-utility analysis. BMC Med 2016;14(1):88. 10.1186/s12916-016-0634-2. 27328975 PMC4916534

[75] Riley KM, Glasgow RE, Eakin EG. Resources for health: a social-ecological intervention for supporting self-management of chronic conditions. J Health Psychol 2001;6(6):693–705. 22049471 10.1177/135910530100600607

[76] Swerissen H, Belfrage J, Weeks A et al. A randomised control trial of a self-management program for people with a chronic illness from Vietnamese, Chinese, Italian and Greek backgrounds. Patient Educ Couns 2006;64(1–3):360–8. 10.1016/j.pec.2006.04.003. 16859871

[77] Willard-Grace R, Chen EH, Hessler D et al. Health coaching by medical assistants to improve control of diabetes, hypertension, and hyperlipidemia in low-income patients: a randomized controlled trial. Ann Fam Med 2015;13(2):130–8. 10.1370/afm.1768. 25755034 PMC4369595

[78] Ricci-Cabello I, Ruiz-Pérez I, Rojas-García A et al. Characteristics and effectiveness of diabetes self-management educational programs targeted to racial/ethnic minority groups: a systematic review, meta-analysis and meta-regression. BMC Endocr Disord 2014;14(1):60. 10.1186/1472-6823-14-60. 25037577 PMC4107728

[79] Kaiafa G, Veneti S, Polychronopoulos G et al. Is HbA1c an ideal biomarker of well-controlled diabetes? Postgrad Med J 2021;97(1148):380–3. 10.1136/postgradmedj-2020-138756. 32913038 PMC10016911

[80] Rookes TA, Schrag A, Walters K et al. Measures of fidelity of delivery and engagement in self-management interventions: a systematic review of measures. Clin Trials 2022;19(6):665–72. 10.1177/17407745221118555. 36017707 PMC9679554

[81] Glanz K, Bishop DB. The role of behavioral science theory in development and implementation of public health interventions. Annu Rev Public Health 2010;31(1):399–418. 10.1146/annurev.publhealth.012809.103604. 20070207

[82] Head A, Fleming K, Kypridemos C et al. Inequalities in incident and prevalent multimorbidity in England, 2004–19: a population-based, descriptive study. Lancet Healthy Longev 2021;2(8):e489–97. 10.1016/S2666-7568(21)00146-X. 36097998

[83] McLean G, Gunn J, Wyke S et al. The influence of socioeconomic deprivation on multimorbidity at different ages: a cross-sectional study. Br J Gen Pract 2014;64(624):e440–7. 10.3399/bjgp14X680545. PMC4073730 24982497

[84] Baig AA, Locklin CA, Wilkes AE et al. Integrating diabetes self-management interventions for Mexican-Americans into the Catholic Church setting. J Relig Health 2014;53(1):105–18. 10.1007/s10943-012-9601-1. 22528288 PMC3430816

[85] Walker EA, Silver LD, Chamany S et al. Baseline characteristics and Latino versus non-Latino contrasts among *Bronx A1C* study participants. West J Nurs Res 2014;36(9):1030–51. 10.1177/0193945913517947. 24407771 PMC4576996

[86] Papajorgji-Taylor D, Francisco M, Schneider JL et al. Bridge to health/Puente a la Salud: rationale and design of a pilot feasibility randomized trial to address diabetes self-management and unmet basic needs among racial/ethnic minority and low-income patients. Contemp Clin Trials Commun 2021;22:100779. 10.1016/j.conctc.2021.100779. 34013093 PMC8114052

[87] Gary TL, Batts-Turner M, Bone LR et al. A randomized controlled trial of the effects of nurse case manager and community health worker team interventions in urban African-Americans with type 2 diabetes. Control Clin Trials 2004;25(1):53–66. 10.1016/j.cct.2003.10.010. 14980748

[88] Greenhalgh T, Collard A, Campbell-Richards D et al. Storylines of self-management: narratives of people with diabetes from a multiethnic inner city population. J Health Serv Res Policy 2011;16(1):37–43. 10.1258/jhsrp.2010.009160. 20819914

[89] Lynch EB, Liebman R, Ventrelle J et al. Design of the Lifestyle Improvement through Food and Exercise (LIFE) study: a randomized controlled trial of self-management of type 2 diabetes among African American patients from safety net health centers. Contemp Clin Trials 2014;39(2):246–55. 10.1016/j.cct.2014.09.003. 25245954 PMC4297207

[90] Nelson K, Drain N, Robinson J et al. Peer support for achieving independence in diabetes (peer-AID): design, methods and baseline characteristics of a randomized controlled trial of community health worker assisted diabetes self-management support. Contemp Clin Trials 2014;38(2):361–9. 10.1016/j.cct.2014.06.011. 24956324 PMC4141876

[91] Pyatak EA, Carandang K, Davis S. Developing a manualized occupational therapy diabetes management intervention. OTJR (Thorofare N J) 2015;35(3):187–94. 10.1177/1539449215584310. 26594741 PMC4801110

[92] Pyatak EA, Carandang K, Vigen C et al. Resilient, empowered, active living with diabetes (REAL diabetes) study: methodology and baseline characteristics of a randomized controlled trial evaluating an occupation-based diabetes management intervention for young adults. Contemp Clin Trials 2017;54:8–17. 10.1016/j.cct.2016.12.025. 28064028 PMC5312749

[93] Rosal MC, White MJ, Restrepo A et al. Design and methods for a randomized clinical trial of a diabetes self-management intervention for low-income Latinos: Latinos en control. BMC Med Res Methodol 2009;9(1):81. 10.1186/1471-2288-9-81. 20003208 PMC2800841

[94] Schillinger D, Hammer H, Wang F et al. Seeing in 3-D: examining the reach of diabetes self-management support strategies in a public health care system. Health Educ Behav 2008;35(5):664–82. 10.1177/1090198106296772. 17513690

[95] Handley MA, Hammer H, Schillinger D. Navigating the terrain between research and practice: a collaborative research network (CRN) case study in diabetes research. J Am Board Fam Med 2006;19(1):85–92. 10.3122/jabfm.19.1.85. 16492010

[96] Shea S, Starren J, Weinstock RS et al. Columbia University’s informatics for diabetes education and telemedicine (IDEATel) project: rationale and design. J Am Med Inform Assoc 2002;9(1):49–62. 10.1136/jamia.2002.0090049. 11751803 PMC349387

[97] Starren J, Hripcsak G, Sengupta S et al. Columbia University’s informatics for diabetes education and telemedicine (IDEATel) project: technical implementation. J Am Med Inform Assoc 2002;9(1):25–36. 10.1136/jamia.2002.0090025. 11751801 PMC349385

[98] Skelly AH, Leeman J, Carlson J et al. Conceptual model of symptom-focused diabetes care for African Americans. J Nurs Scholarsh 2008;40(3):261–7. 10.1111/j.1547-5069.2008.00236.x. 18840210 PMC2567121

[99] Feathers JT, Kieffer EC, Palmisano G et al. The development, implementation, and process evaluation of the REACH Detroit Partnership’s diabetes lifestyle intervention. Diabetes Educ 2007;33(3):509–20. 10.1177/0145721707301371. 17570882

[100] Ghorob A, Vivas MM, De Vore D et al. The effectiveness of peer health coaching in improving glycemic control among low-income patients with diabetes: protocol for a randomized controlled trial. BMC Public Health 2011;11(1):208. 10.1186/1471-2458-11-208. 21457567 PMC3082244

[101] Piette J, Valenstein M, Eisenberg D et al. Rationale and methods of a trial to evaluate a depression telemonitoring program that includes a patient-selected support person. J Clin Trials 2014;05(01). 10.4172/2167-0870.1000205. PMC6388705 30815325

[102] Apter AJ, Bryant-Stephens T, Morales KH et al. Using IT to improve access, communication, and asthma in African American and Hispanic/Latino adults: rationale, design, and methods of a randomized controlled trial. Contemp Clin Trials 2015;44:119–28. 10.1016/j.cct.2015.08.001. 26264737

[103] Goldfinger JZ, Kronish IM, Fei K et al. Peer education for secondary stroke prevention in inner-city minorities: design and methods of the prevent recurrence of all inner-city strokes through education randomized controlled trial. Contemp Clin Trials 2012;33(5):1065–73. 10.1016/j.cct.2012.06.003. 22710563 PMC3408803

[104] Lorig KR, Mazonson PD, Holman HR. Evidence suggesting that health education for self-management in patients with chronic arthritis has sustained health benefits while reducing health care costs. Arthritis Rheum 1993;36(4):439–46. 10.1002/art.1780360403 8457219

[105] Kangovi S, Mitra N, Smith RA et al. Decision-making and goal-setting in chronic disease management: baseline findings of a randomized controlled trial. Patient Educ Couns 2017;100(3):449–55. 10.1016/j.pec.2016.09.019. 27717532 PMC5437864

[106] Kangovi S, Mitra N, Turr L et al. A randomized controlled trial of a community health worker intervention in a population of patients with multiple chronic diseases: study design and protocol. Contemp Clin Trials 2017;53:115–21. 10.1016/j.cct.2016.12.009. 27965180 PMC5455773

[107] Bower P, Kennedy A, Reeves D et al. A cluster randomised controlled trial of the clinical and cost-effectiveness of a “whole systems” model of self-management support for the management of long-term conditions in primary care: trial protocol. Implement Sci 2012;7(1):7. 10.1186/1748-5908-7-7. 22280501 PMC3274470

[108] Bikker A, Mercer S, Cotton P. Connecting, assessing, responding and empowering (CARE): a universal approach to person-centred, empathic healthcare encounters. Educ Prim Care 2012;23(6):454. 23232141

[109] Bodenheimer T, Laing BY. The teamlet model of primary care. Ann Fam Med 2007;5(5):457–61. 10.1370/afm.731. 17893389 PMC2000308

[110] Willard-Grace R, Devore D, Chen EH et al. The effectiveness of medical assistant health coaching for low-income patients with uncontrolled diabetes, hypertension, and hyperlipidemia: protocol for a randomized controlled trial and baseline characteristics of the study population. BMC Fam Pract 2013;1:1–10. 10.1186/1471-2296-14-27. PMC3616979 23433349

