# Supplementary material for: Effectiveness of self-management interventions for long-term conditions in people experiencing socio-economic deprivation in high-income countries: a systematic review and meta-analysis
Source: J Public Health (Oxf). 2023 Aug 8;45(4):970–1041. doi: 10.1093/pubmed/fdad145 (PMC10687879; doi:10.1093/pubmed/fdad145)
Supplement: Supp_2_fdad145 [file supp_2_fdad145.docx]

*Supplementary material 2: GRADE Assessment*

|  | | | | | | **Summary of Findings** | | | |
| --- | --- | --- | --- | --- | --- | --- | --- | --- | --- |
| **Quality Assessment** | | | | | | **Number of participants** | | **Mean change (95% confidence interval)** | **Certainty of evidence** |
| **Number of studies (design)** | **Risk of Bias** | **Inconsistency** | **Indirectness** | **Imprecision** | **Publication Bias** | **Self-management intervention** | **Control** |  |  |
| **Mean change in HbA1c%** | | | | | | | |  |  |
| Thirteen (RCT and pilot studies) | Serious ^a^ | Not serious ^b^ | Not serious ^c^ | Not serious ^d^ | Undetected ^e^ | 1239 | 1255 | -0.29 (-0.48 to -0.10) | Mode rate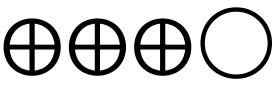 |

^a^ 9 out of 13 studies had moderate or high risk of methodological bias. However, this was mainly due to insufficient outcome data.

^b^ I^2^ = 32.46%, which suggests low heterogeneity. Cochrane Q- P=0.16, which provide strong evidence for homogeneity. Majority of confidence intervals overlap. Consistent direction of effect, which favours the intervention groups.

^c^ Similar population across all studies. While HbA1c could be considered a surrogate outcome, it is well established as a marker of improvement in diabetes control.

^d^ 13 studies (>10) included in the meta-analysis. The magnitude of the median sample size (110) is intermediate. The confidence interval of the pooled difference does not cross the line of null effect.

^e^ Funnel plot is mostly symmetrical and the Egger test for funnel plot asymmetry suggest that there is no evidence of small study effects (P-0.2355). This review used a comprehensive search strategy. However, we excluded non-English texts and did not include grey literature.
