# Supplementary figures and images for: Effectiveness of self-management interventions for long-term conditions in people experiencing socio-economic deprivation in high-income countries: a systematic review and meta-analysis
Source: J Public Health (Oxf). 2023 Aug 8;45(4):970–1041. doi: 10.1093/pubmed/fdad145 (PMC10687879; doi:10.1093/pubmed/fdad145)

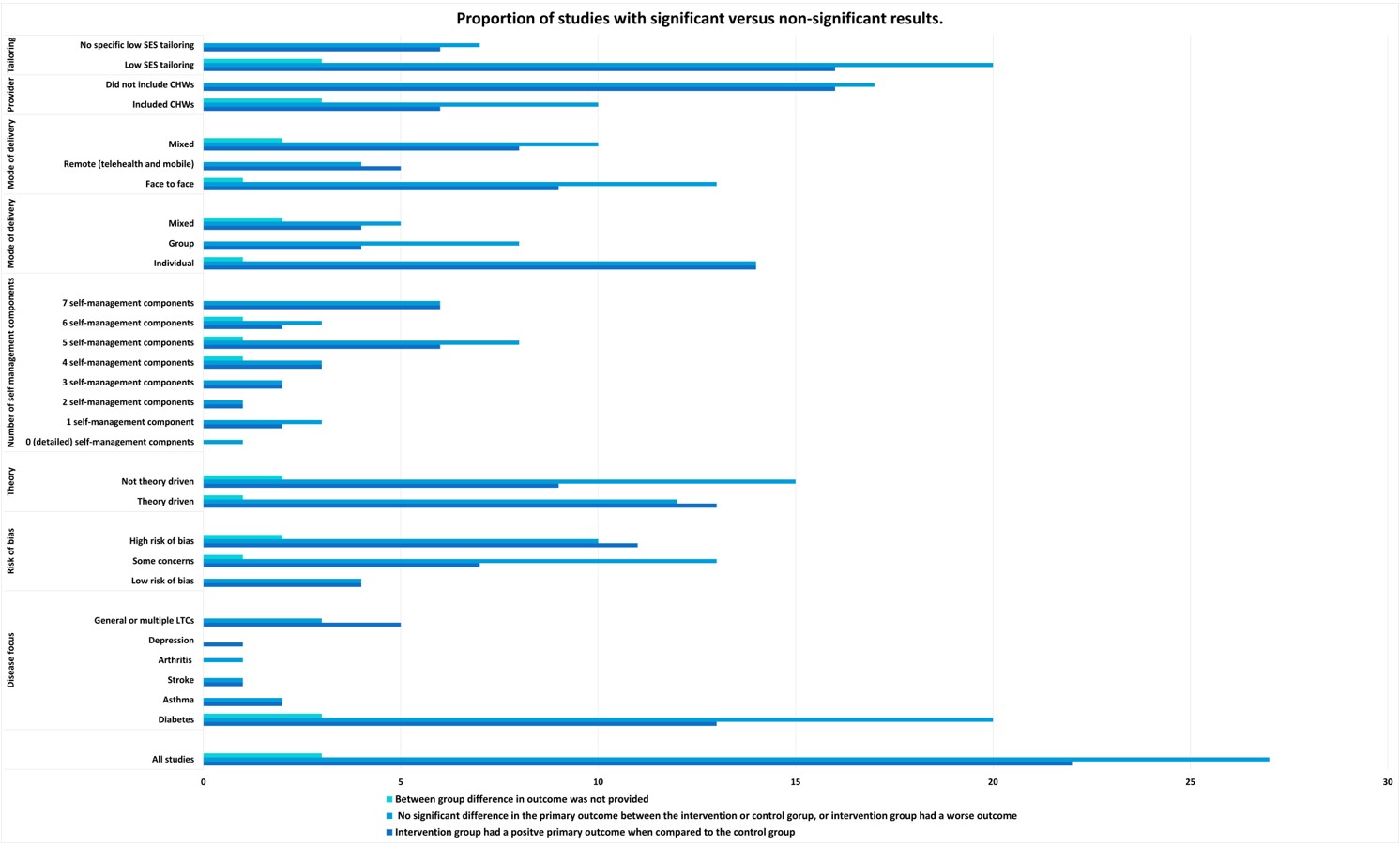

Supplement: supp_3_fdad145 [file supp_3_fdad145.jpeg]

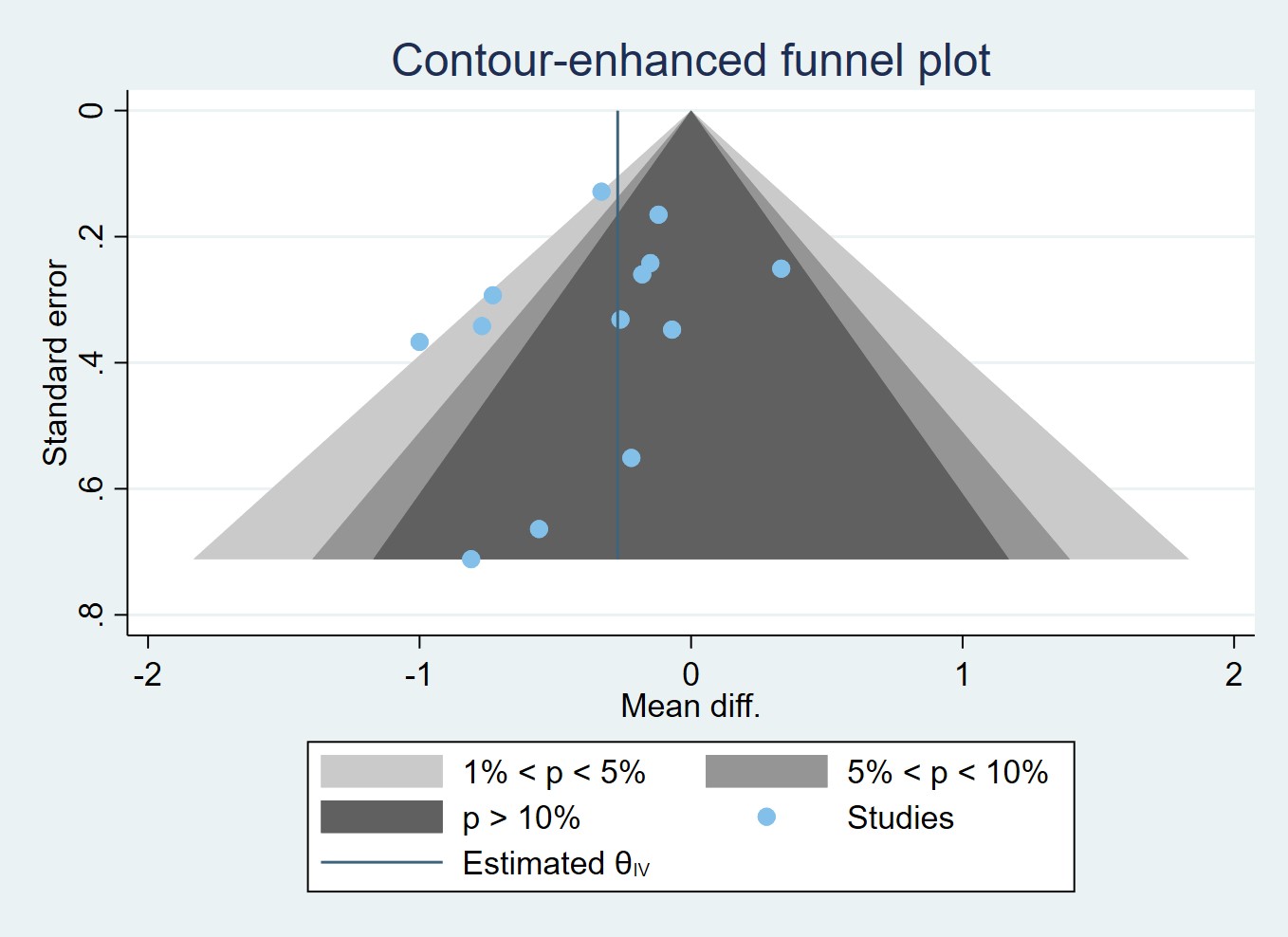

Supplement: supp_4_fdad145 [file supp_4_fdad145.jpeg]
