# Supplementary material for: Effectiveness of self-management interventions for long-term conditions in people experiencing socio-economic deprivation in high-income countries: a systematic review and meta-analysis
Source: J Public Health (Oxf). 2023 Aug 8;45(4):970–1041. doi: 10.1093/pubmed/fdad145 (PMC10687879; doi:10.1093/pubmed/fdad145)
Supplement: SM_review-appendix_fdad145 [file sm_review-appendix_fdad145.docx]

**Supplementary appendix**

Supplementary Table 1: Example search strategy

| **#** | **CONCEPT** | **Sub-group** | **Query** | **AMED** | **EMBASE** | **MEDLINE** | **PsycINFO** |
| --- | --- | --- | --- | --- | --- | --- | --- |
| 1 | **Self-management** | N/A | "self-manag*".ab,ti. | 863 | 32,961 | 23,066 | 10,931 |
| 2 |  |  | "self-monitor*".ab,ti. | 153 | 12,370 | 8,885 | 6,382 |
| 3 |  |  | "self-care".ab,ti. | 1,195 | 28,859 | 20,494 | 10,519 |
| 4 |  |  | "self-help".ab,ti. | 369 | 8,752 | 6,926 | 8,609 |
| 5 |  |  | "self-maintenance".ab,ti. | 15 | 835 | 700 | 378 |
| 6 |  |  | self-maintain.ab,ti. | 0 | 68 | 38 | 10 |
| 7 |  |  | 1 or 2 or 3 or 4 or 5 or 6 | 2,500 | 79,116 | 56,689 | 35,274 |
| 8 | **Long-term conditions** | Multi-morbidity | "multiple chronic conditions".ab,ti. | 24 | 1,631 | 1,331 | 359 |
| 9 |  |  | co-morbid*.ab,ti. | 316 | 68,933 | 29,769 | 7,164 |
| 10 |  |  | co?morbid*.ab,ti. | 1,878 | 322,602 | 187,462 | 55,401 |
| 11 |  |  | multi?morbid*.ab,ti. | 61 | 8,069 | 6,198 | 1,089 |
| 12 |  |  | multi-morbid*.ab,ti. | 7 | 1,567 | 955 | 194 |
| 13 |  |  | polychronic*.ab,ti. | 0 | 22 | 24 | 165 |
| 14 |  |  | poly?morbid*.ab,ti. | 2 | 625 | 397 | 16 |
| 15 |  |  | poly-morbid*.ab,ti. | 0 | 22 | 18 | 3 |
| 16 |  |  | "co-occurring condition*".ab,ti. | 5 | 553 | 403 | 364 |
| 17 |  |  | "concurrent condition*".ab,ti. | 2 | 413 | 290 | 83 |
| 18 |  |  | 8 or 9 or 10 or 11 or 12 or 13 or 14 or 15 or 16 or 17 | 2,259 | 392,271 | 222,354 | 63,478 |
| 19 |  | Chronic illness | "chronic illness*".ab,ti. | 730 | 23,118 | 16,927 | 9,779 |
| 20 |  |  | "chronic disease*".ab,ti. | 1,231 | 100,197 | 71,903 | 11,865 |
| 21 |  |  | "chronic condition*".ab,ti. | 550 | 28,757 | 21,120 | 5,973 |
| 22 |  |  | NCD*.ab,ti. | 20 | 12,780 | 8,027 | 662 |
| 23 |  |  | "noncommunicable disease*".ab,ti. | 28 | 3,942 | 3,429 | 340 |
| 24 |  |  | "non-communicable disease*".ab,ti. | 39 | 11,004 | 8,785 | 790 |
| 25 |  |  | "noncommunicable illness*".ab,ti. | 0 | 9 | 7 | 1 |
| 26 |  |  | "non-communicable illness*".ab,ti. | 0 | 12 | 11 | 1 |
| 27 |  |  | "long-term disease*".ab,ti. | 22 | 5,762 | 3,588 | 144 |
| 28 |  |  | "long-term illness*".ab,ti. | 32 | 879 | 779 | 359 |
| 29 |  |  | "long-term condition*".ab,ti. | 102 | 2,816 | 2,113 | 700 |
| 30 |  |  | 19 or 20 or 21 or 22 or 23 or 24 or 25 or 26 or 27 or 28 or 29 | 2,614 | 176,214 | 126,382 | 28,182 |
| 31 |  | Specific conditions-NCDs | diabetes.ab,ti. | 4,365 | 872,646 | 573,632 | 31,243 |
| 32 |  |  | "cardiovascular disease*".ab,ti. | 1,256 | 275,588 | 191,031 | 11,920 |
| 33 |  |  | CVD.ab,ti. | 181 | 65,321 | 41,605 | 3,115 |
| 34 |  |  | "coronary artery disease".ab,ti. | 349 | 142,176 | 89,762 | 2,028 |
| 35 |  |  | "chronic respiratory disease".ab,ti. | 77 | 3,295 | 2,325 | 104 |
| 36 |  |  | "chronic obstructive pulmonary disease".ab,ti. | 1,292 | 77,229 | 53,293 | 2,347 |
| 37 |  |  | COPD.ab,ti. | 1,404 | 96,680 | 50,971 | 1,714 |
| 38 |  |  | asthma.ab,ti. | 1,759 | 226,965 | 155,603 | 7,615 |
| 39 |  |  | cancer.ab,ti. | 14,348 | 2,655,179 | 1,885,284 | 64,642 |
| 40 |  |  | cancers.ab,ti. | 794 | 424,662 | 294,492 | 4,648 |
| 41 |  |  | dementia.ab,ti. | 2,602 | 172,890 | 119,556 | 67,345 |
| 42 |  |  | epilepsy.ab,ti. | 663 | 159,653 | 110,905 | 33,557 |
| 43 |  |  | stroke.ab,ti. | 9,383 | 428,109 | 267,852 | 34,528 |
| 44 |  |  | multiple sclerosis.ab,ti. | 2,108 | 126,551 | 81,318 | 16,182 |
| 45 |  |  | Parkinson's.ab,ti. | 1,720 | 142,023 | 97,471 | 27,944 |
| 46 |  |  | "motor neuron disease".ab,ti. | 39 | 6,712 | 4,603 | 949 |
| 47 |  |  | neurodegenerative.ab,ti. | 401 | 131,565 | 98,026 | 20,987 |
| 48 |  |  | arthritis.ab,ti. | 3,840 | 274,309 | 185,921 | 5,460 |
| 49 |  |  | "inflammatory arthropathies".ab,ti. | 4 | 613 | 376 | 2 |
| 50 |  |  | osteoporosis.ab,ti. | 1,170 | 111,471 | 71,586 | 1,920 |
| 51 |  |  | "liver disease".ab,ti. | 300 | 146,688 | 92,148 | 1,627 |
| 52 |  |  | cirrhosis.ab,ti. | 150 | 147,772 | 96,545 | 1,160 |
| 53 |  |  | 31 or 32 or 33 or 34 or 35 or 36 or 37 or 38 or 39 or 40 or 41 or 42 or 43 or 44 or 45 or 46 or 47 or 48 or 49 or 50 or 51 or 52 | 43,710 | 5,665,918 | 3,924,749 | 294,133 |
| 54 |  | Specific conditions-Long term infectious | HIV.ab,ti. | 1,867 | 423,586 | 330,382 | 54,651 |
| 55 |  |  | "human immunodeficiency virus".ab,ti. | 329 | 98,785 | 90,387 | 5,999 |
| 56 |  |  | AIDS.ab,ti. | 2,235 | 180,893 | 154,951 | 36,405 |
| 57 |  |  | "acquired immunodeficiency syndrome".ab,ti. | 93 | 17,008 | 16,302 | 749 |
| 58 |  |  | 54 or 55 or 56 or 57 | 3,481 | 542,095 | 430,993 | 71,928 |
| 59 |  | Specific conditions- mental health | depression.ab,ti. | 6,891 | 505,662 | 368,524 | 260,011 |
| 60 |  |  | anxiety.ab,ti. | 4,787 | 310,956 | 220,001 | 206,821 |
| 61 |  |  | "anxiety disorder".ab,ti. | 213 | 24,236 | 17,678 | 17,482 |
| 62 |  |  | schizophrenia.ab,ti. | 1,013 | 154,859 | 114,261 | 105,369 |
| 63 |  |  | "schizophrenic disorder*".ab,ti. | 10 | 1,505 | 1,083 | 1,309 |
| 64 |  |  | bipolar.ab,ti. | 345 | 97,489 | 67,414 | 41,308 |
| 65 |  |  | "bipolar disorder".ab,ti. | 134 | 44,120 | 28,745 | 26,201 |
| 66 |  |  | "bipolar affective disorder".ab,ti. | 16 | 3,066 | 2,136 | 2,001 |
| 67 |  |  | "panic disorder".ab,ti. | 89 | 12,100 | 9,382 | 10,484 |
| 68 |  |  | 59 or 60 or 61 or 62 or 63 or 64 or 65 or 66 or 67 | 10,571 | 868,326 | 637,895 | 503,207 |
| 69 |  | ***Subtotal*** | 18 or 30 or 53 or 58 or 68 | 57,655 | 7,119,971 | 5,035,541 | 855,488 |
| 70 | **SES** | Social determinant concept | "social inequalities".ab,ti. | 7 | 2,842 | 2,700 | 1,437 |
| 71 |  |  | "health inequalities".ab,ti. | 26 | 5,659 | 5,189 | 1,732 |
| 72 |  |  | "health inequit*".ab,ti. | 15 | 3,045 | 2,867 | 877 |
| 73 |  |  | "social gradient".ab,ti. | 3 | 847 | 745 | 265 |
| 74 |  |  | "social determinants".ab,ti. | 33 | 10,402 | 8,811 | 3,330 |
| 75 |  |  | 70 or 71 or 72 or 73 or 74 | 82 | 21,177 | 18,741 | 7,098 |
| 76 |  | Socio-economic deprivation terms | socio-economic.ab,ti. | 269 | 45,735 | 34,970 | 14,404 |
| 77 |  |  | socioeconomic.ab,ti. | 658 | 124,187 | 101,323 | 48,869 |
| 78 |  |  | social-economic.ab,ti. | 151 | 6,068 | 4,970 | 3,591 |
| 79 |  |  | SES.ab,ti. | 126 | 25,729 | 18,329 | 18,241 |
| 80 |  |  | "social class".ab,ti. | 79 | 9,471 | 8,772 | 10,098 |
| 81 |  |  | "working class".ab,ti. | 15 | 1,355 | 1,284 | 4,233 |
| 82 |  |  | "social status".ab,ti. | 63 | 7,691 | 6,384 | 7,638 |
| 83 |  |  | "social position".ab,ti. | 7 | 1,213 | 1,090 | 1,438 |
| 84 |  |  | "low-status".ab,ti. | 12 | 930 | 884 | 1,821 |
| 85 |  |  | "social* depriv*".ab,ti. | 21 | 3,097 | 2,317 | 1,051 |
| 86 |  |  | "material* depriv*".ab,ti. | 5 | 746 | 628 | 298 |
| 87 |  |  | "socially excluded".ab,ti. | 10 | 302 | 240 | 413 |
| 88 |  |  | "social* exclusion".ab,ti. | 37 | 2,445 | 2,021 | 3,325 |
| 89 |  |  | 76 or 77 or 78 or 79 or 80 or 81 or 82 or 83 or 84 or 85 or 86 or 87 or 88 | 1,365 | 204,491 | 164,135 | 99,333 |
| 90 |  | Vulnerable group terms | vulnerable*.ab,ti. | 823 | 125,116 | 97,953 | 40,247 |
| 91 |  |  | disadvantaged.ab,ti. | 175 | 17,241 | 15,189 | 14,964 |
| 92 |  |  | underserved.ab,ti. | 180 | 15,911 | 11,549 | 5,525 |
| 93 |  |  | marginali#ed.ab,ti. | 129 | 7,341 | 6,500 | 10,232 |
| 94 |  |  | marginali#ation.ab,ti. | 56 | 2,902 | 2,615 | 4,843 |
| 95 |  |  | "less affluent".ab,ti. | 4 | 515 | 427 | 215 |
| 96 |  |  | under-represent*.ab,ti. | 8,210 | 6,522 | 4,686 | 1,910 |
| 97 |  |  | "hard-to-reach".ab,ti. | 11 | 2,893 | 2,337 | 1,076 |
| 98 |  |  | 90 or 91 or 92 or 93 or 94 or 95 or 96 or 97 | 9,455 | 173,435 | 137,125 | 75,703 |
| 99 |  | Subgroup: education | "low* educat*".ab,ti. | 117 | 22,120 | 17,267 | 7,059 |
| 100 |  |  | "less educat*".ab,ti. | 80 | 7,905 | 6,518 | 3,550 |
| 101 |  |  | "education* level".ab,ti. | 411 | 48,565 | 34,985 | 16,657 |
| 102 |  |  | "education* status".ab,ti. | 52 | 6,712 | 4,907 | 1,602 |
| 103 |  |  | "education* attainment".ab,ti. | 83 | 11,426 | 9,778 | 7,607 |
| 104 |  |  | "higher educat*".ab,ti. | 418 | 23,989 | 19,128 | 31,447 |
| 105 |  |  | 99 or 100 or 101 or 102 or 103 or 104 | 1,038 | 98,308 | 75,145 | 61,041 |
| 106 |  | Subgroup -occupation (inc. unemployment) | unemploy*.ab,ti. | 536 | 25,994 | 20,267 | 15,830 |
| 107 |  |  | occupation.ab,ti. | 1,716 | 41,194 | 33,853 | 14,866 |
| 108 |  |  | "insecure employment".ab,ti. | 1 | 35 | 36 | 33 |
| 109 |  |  | 106 or 107 or 108 | 2,232 | 66,181 | 53,406 | 30,424 |
| 110 |  | Subgroup- housing (inc. homelessness | "housing".ab,ti. | 607 | 37,666 | 32,393 | 18,394 |
| 111 |  |  | "permanent supportive housing".ab,ti. | 5 | 150 | 151 | 128 |
| 112 |  |  | "social housing".ab,ti. | 3 | 628 | 497 | 373 |
| 113 |  |  | "council-hous*".ab,ti. | 2 | 54 | 46 | 23 |
| 114 |  |  | homeless*.ab,ti. | 339 | 14,753 | 12,067 | 11,685 |
| 115 |  |  | 110 or 111 or 112 or 113 or 114 | 856 | 49,562 | 42,129 | 27,447 |
| 116 |  | Proxy: area of deprivation | deprivation.ab,ti. | 315 | 93,254 | 72,056 | 24,187 |
| 117 |  |  | "disadvantaged area".ab,ti. | 3 | 160 | 137 | 94 |
| 118 |  |  | "low-income neighbo?rhood".ab,ti. | 1 | 180 | 156 | 134 |
| 119 |  |  | 116 or 117 or 118 | 319 | 93,579 | 72,339 | 24,408 |
| 120 |  | Proxy: Income | low-income.ab,ti. | 236 | 46,511 | 39,945 | 22,626 |
| 121 |  |  | low-resource.ab,ti. | 33 | 9,986 | 7,706 | 1,084 |
| 122 |  |  | limited-resource.ab,ti. | 5 | 2,335 | 1,614 | 468 |
| 123 |  |  | impoverished.ab,ti. | 38 | 4,318 | 3,865 | 3,672 |
| 124 |  |  | indigent.ab,ti. | 14 | 2,496 | 2,203 | 504 |
| 125 |  |  | uninsured.ab,ti. | 29 | 12,501 | 8,794 | 1,837 |
| 126 |  |  | underinsured.ab,ti. | 8 | 1,141 | 774 | 174 |
| 127 |  |  | under-insured.ab,ti. | 90 | 121 | 56 | 19 |
| 128 |  |  | "social assistance".ab,ti. | 18 | 1,029 | 835 | 493 |
| 129 |  |  | "welfare-assist*".ab,ti. | 1 | 101 | 97 | 77 |
| 130 |  |  | "state-support*".ab,ti. | 13 | 700 | 599 | 488 |
| 131 |  |  | "state-benefits".ab,ti. | 3 | 87 | 75 | 60 |
| 132 |  |  | "income support".ab,ti. | 8 | 354 | 311 | 245 |
| 133 |  |  | subsidi#ed.ab,ti. | 27 | 3,944 | 3,101 | 1,125 |
| 134 |  |  | "financially disadvantaged".ab,ti. | 1 | 109 | 82 | 54 |
| 135 |  |  | 120 or 121 or 122 or 123 or 124 or 125 or 126 or 127 or 128 or 129 or 130 or 131 or 132 or 133 or 134 | 498 | 81,323 | 66,693 | 31,826 |
| 136 |  | ***Subtotal*** | 75 or 89 or 98 or 105 or 109 or 115 or 119 or 135 | 14,925 | 689,722 | 550,817 | 315,210 |
| 137 | **TOTAL** | | 7 and 69 and 136 | 85 | 4,394 | 2,907 | 1,208 |

Supplementary Table 2: Exclusion table from full text screening (December 2021 search)

| **Citation (author, date)** | **Reason for exclusion** |
| --- | --- |
| Abbot, 2021 | Remove as secondary analysis - intervention not specifically for low SES |
| Aguilera et al., 2020 | protocol only/ trial in progress |
| Aikens et al, 2021 | Remove? |
| Altaf et al., 2020 | conferance abstract/ no full text |
| Amoako, 2004 | dissertation |
| Anderson et al., 2019 | conferance abstract/ no full text |
| Anderson-Loftin et al., 2005 | Ethnic minority only |
| Bailey et al., 2020 | protocol only/ trial in progress |
| Baviskar et al., 2021 | Slum area-India |
| Bodenhamer et al., 2010 | Conference |
| Brown et al., 2013 | Remove? |
| Callender et al. 2012 | Hypertension only |
| Carpenter et al., 2017 | survey study |
| Castillo-Hernandez et al., 2021 | Remove? Is comparing self-management + peer support vs self-management only |
| Chakkalakal et al., 2017 | secondary analysis |
| Chan et al., 2015 | Remove - includes no LTCs as well, and no results are reported (protocol?) |
| Chan, 2013 | dissertation |
| Chao et al., 2015 | Sample and intervention not SES specific- just older adults |
| Charlson et al., 2021 | protocol only/ trial in progress |
| Cheng et al., 2018 | set in LMIC (China) |
| Chouinard et al., 2013 | protocol only/ trial in progress |
| Conneely, et al. 2017. | conferance abstract/ no full text |
| Contant et al., 2019 | Studied effects of SES on the outcomes by adjusting for income and education. Interention not targeted to low SES groups |
| Creason et al., 2018 | Conference |
| Crowley et al., 2013 | Ethnic minority only |
| Cruz et al., 2009 | Conference |
| Cummings et al., 2013 | protocol only/ trial in progress/ incomplete |
| Dash et al., 2015 | Sample age group begins at 15+ (includes teens) + set in LMIC (India) |
| Davis et al., 2009 | Conference |
| De Vries et al., 2014 | conference |
| Debussche et al., 2012 | Reunion Island |
| Debussche et al., 2018 | Mali |
| DeWalt et al., 2004 | Sample and intervention not SES specific |
| Dowe, 2013 | dissertation |
| Dwyer et al., 2020 | protocol only/ trial in progress/ incomplete |
| Ehrlich-Jones, 2001 | full text unavailable |
| Ell et al., 2009 | protocol only/ trial in progress/ incomplete |
| Ell et al., 2010 | depression treatment not self-management |
| Fanning, 2002 | dissertation |
| Farmer et al, 2021 | set in two LMICs (South Africa + Malawi) |
| Fischer et al., 2008 | protocol only/ trial in progress/ incomplete |
| Fischer et al., 2011 |  |
| Foley et al., 2016 | primarily weight loss only intervention |
| Fortmann et al., 2015 | Conference |
| Fries et al. 2005 | Intervention is preventative |
| Garciaet al 2020 | Conference |
| Goeppinger et al., 2009 | Ethnic minority only |
| Gold et al., 2008 | Ethnic minority only |
| Gregg et al., 2007 | Remove |
| Gregg, 2004 | dissertation |
| Griva, 2019 | sample is Not low SES/ Low-income |
| Gross et al., 2011 | full text unavailable/conference abstract |
| Hafdi et al., 2021 | Intervention is preventative |
| Heisler et al., 2013 | full text unavailable/conference abstract |
| Hnasen, 2000 | dissertation |
| Janevic et al., 2012 | protocol only/ trial in progress/ incomplete |
| Jones et al., 2019 | full text unavailable/conference abstract |
| Kandula et al., 2015 | Exclude - at risk pop |
| Kim et al., 2014 | full text unavailable/conference abstract |
| Kim et al., 2015 | Ethnic minority only |
| King et al., 2019 | intervention is smoking cessation not SM |
| Kumar et al., 2019 | Remove - comparison of white vs black americans in larger trial |
| Lachance et al., 2018 | Intervention is preventative |
| Landa-Gonzalez and Molnar, 2012 | intervention is not SM (occupational therapy) |
| Lange et al., 2010 | English text unavailable /conference abstract |
| Lara et al., 2003 | Remove - not randomised |
| Lee et al., 2016 | qualitative desgin + diabetes only |
| lerman et al., 2009 | English text unavailable /conference abstract |
| Levine et al., 2016 | Remove |
| Li et al., 2018 | Set in LMIC china |
| Lujan 2006 | dissertation |
| Lynch et al., 2016 | Protocol only, no results |
| Mabweazara et al., 2018 | Set in LMIC south africa |
| Mammen et al., 2018 | full text unavailable/conference abstract |
| Marcello et al., 2020 | full text unavailable/conference abstract |
| Mash et al., 2014 | Set in LMIC south africa |
| May et al., 2013 | full text unavaible/conference abstract |
| Mayberry et al., 2016 | qualitative desgin on intervention feasabilty |
| McCall, et al. 2021 | app usabilty study only |
| Miles et al., 2003 | Exclude - not self-management components more self-care |
| Nelson et al., 2021 | Remove - aim was not for this population but to compare in between group effects |
| Olry-de-labry-lima et al., 2017 | Remove - one off sessions on benefits of self-management |
| Osborn et al., 2013 | qualitative desgin on intervention feasabilty |
| Owolabi et al., 2019 | Set in LMIC south africa |
| Page-Reeves et al., 2017 | Remove protocol |
| Pekmezaris et al., 2019 | Remove - self-monitoring only |
| Philis-Tsimikas et al., 2016 | repeat publication of the dulce digital (fortmann et al) |
| Piatt et al., 2015 | full text unavailable/conference abstract |
| Piatt et al., 2021 | full text unavailable /conference abstract |
| Powell et al., 2010 | sample not SES specifc |
| Quach et al., 2014 | full text unavailable/conference abstract |
| Ramirez et al., 2017 | Only self-management behaviour addressed was physical activity (step count) |
| Robertson et al., 2013 | Seconday logistic regression analysis of trial |
| Ruggiero et al., 2011 | Non- randomised third arm, not RCT |
| Ryabov, 2011 | not RCT, subjects were matched not randomised into their group assignment. |
| Shahid et al., 2015 | set in LMIC (Pakistan) |
| Situ et al., 2019 | full text unavailable/conference abstract |
| Thanh et al., 2021 | Set in LMIC vietnam |
| Tu et al., 2011 | full text unavailable/conference abstract |
| Turner et al., 2018 | focus is on chronic pain |
| Van et al., 2017 | set in LMICs (Cambodia, Phillipines and DRC) |
| Van Rompay et al., 2008 | set in LMIC (India) |
| Walker et al., 2009 | full text unavailable/conference abstract |
| Wang et al., 2012 | Population is diabetes OR hypertention. Intervention focus is weight loss |
| Yang et al., 2016 | full text unavailable/conference abstract |

Supplementary table 3: Study characteristics

| Ref | Author, date | Country | Follow up time | Long term condition | Population socioeconomic status | Sample size (intervention/ control) | Mean age (SD) | n Female (%) | Primary Ethnicity (%) | Outcome | Results summary | P-value |
| --- | --- | --- | --- | --- | --- | --- | --- | --- | --- | --- | --- | --- |
| ^26^ | Anderson et al., 2010 | USA | 12 months | Type 2 Diabetes | Area of deprivation: Patients are at or below 200% of the federal poverty level. | 295 (146/149) | NP | 171 (58) | African American and Hispanic/ Latino | Mean HbA1c value | The difference in HbA1c between the intervention and control group was not significant. | P=0.63 |
| ^27^ | Arora et al., 2013 | USA | 6 months | Type 2 Diabetes | Area of deprivation: Low income patients of a safety-net hospital. | 128 (64/64) | 50.7 (10.2) | 82 (64) | Hispanic/ Latino (87) | Mean change in HbA1c (%) | The intervention group had a -0.45 (95% CI: –0.27 to 1.17) greater decrease in HbA1c levels compared to control. | P=0.230 |
| ^28^ | Baig et al., 2015 | USA | 6 months | Type 2 Diabetes | Area of deprivation: low income neighbourhood | 100 (50/50) | 53.7 (11.6) | 81 (81) | Hispanic/ Latino (97.9) | Mean change in HbA1c (%) | The intervention group had a -0.21 (CI: -0.98 to 0.55) greater decrease in HbA1c levels compared to control. | P>0.05 |
| ^29^ | Berry et al., 2016 | USA | 15 months | Type 2 Diabetes | Low income: Annual household income <200% of federal poverty guidelines. | 80 (40/40) | 51.4 (8.5) | 72 (89.3) | African American (77.4) | Mean change in HbA1c (%) | Patients in the experimental group decreased their HbA1C significantly more than the control group. | P=0.001 |
| ^30^ | Chamany et al., 2015 | USA | 12 months | Type 1 and 2 Diabetes | Low income | 941 (443/ 498) | 56.3 (11.7) | 599 (63.7) | Hispanic/ Latino (67.7) | Mean change in HbA1c (%) | The intervention group had a 0.4% greater mean decrease in HbA1c compared to the control group. | P=0.01 |
| ^31^ | Clancy et al., 2007 | USA | 12 months | Type 2 Diabetes | Inadequately insured patients | 186 (96/90) | 56.1 | 134 (72%) | African American (82.8) | Mean change in HbA1c (%) | There was no difference in HbA1c change between the groups over 12 months. | NP |
| ^32^ | Davis et al., 2010 | USA | 12 months | Type 2 Diabetes | Area of deprivation: rural, medically underserved, and low income | 165 (85/80) | 59.9 (9.4) / 59.2 (9.3)** | 123 (75%) | African American (75.3/72.5) | Mean change in HbA1c (%) | The improvement in HbA1c was greater in the intervention group compared with usual care. | P=0.004 |
| ^33^ | Fitzpatrick et al., 2022 | USA | 6 months | Type 2 Diabetes | At least one of the four social risks (food insecurity, unstable housing, difficulty paying for medical care, and lack of transportation). | 110 (56/54) | 53.3 (12) | 77 (70) | Multi-ethnic | Mean change in HbA1c (%) | Within each group there was a clinically significant reduction in HbA1c. -0.72% in the intervention group and -0.54% in the control. | Between group difference not reported. |
| ^34^ | Fortmann al., 2017 | USA | 6 months | Type 2 Diabetes | Low income, uninsured and low educational attainment | 126 (63/63) | 48.43 (9.80) | 94 (75%) | Hispanic/ Latino (100) | Mean change in HbA1c (%) | There was a significant time-by-group interaction effect for HbA1c, indicating that over time, the intervention group had greater glycaemic control, compared to the control group. | P=0.03 |
| ^35^ | Frosch et al., 2011 | USA | 6 months | Type 2 Diabetes | Low income and underinsured | 201 (100/101) | 56.7 (8.3)/ 54.3 (8.9) | 97 (48.3%) | Hispanic/ Latino (55.80) | Mean change in HbA1c (%) | There was an overall decrease in HbA1c values for both groups. However, there was no significant interaction effect of group by time. | P=0.49 |
| ^36^ | Gary et al., 2009 | USA | 24 months | Type 2 Diabetes | "Socioeconomically disadvantaged" | 488 (235/ 253) | 58 (11) | 358 (73) | African American (100) | Mean change in HbA1c (%) | There were no within group or between group differences in HbA1c change. | P=0.44 |
| ^37^ | Greenhalgh et al., 2011 | UK | 6 months | Type 2 Diabetes | socioeconomically deprived area | 157 (79/78) | 58 (12) | 110 (70) | Multi-ethnic | Mean change in HbA1c (%)* | There was no significant difference in the within group change in HbA1c between the intervention and control. | P=0.364 |
| ^38^ | Hill-briggs et al., 2011 | USA | 9 months | Type 2 Diabetes | Low-income | 56 (29/27) | 61.3 (10.9) | 33 (58.9) | African American (100) | Mean change in HbA1c (%) | The intervention group had a larger reduction in HbA1C change. | P=0.02 |
| ^39^ | Lynch et al., 2014 | USA | 6 months | Type 2 Diabetes | low-income | 61 (30/31) | 54.1 (10.0) | 41 (67.2) | African American (100) | Mean change in HbA1c (%) * | There was no significant difference in HbA1c reduction between the groups. | P = 0.10 |
| ^40^ | Lynch et al., 2018 | USA | 12 months | Type 2 Diabetes | low-income | 211 (106/105) | 55.0 (10.3) | 148 (70.1) | African American (100) | Mean change in HbA1c (%) | While the HbA1c change was greater in the intervention group than the comparison group, the difference was not statistically significant. | P=0.52 |
| ^41^ | Nelson et al., 2017 | USA | 12 months | Type 2 Diabetes | Low-income: household income of less than 250% of the federal poverty level. | 287 (145/142) | 52.5 (9.3) | 140 (48.8) | Multi-ethnic | Mean change in HbA1c (%) | There was no significant difference in the mean HbA1c change in the intervention group compared to the control group. | P=0.54 |
| ^42^ | Pérez-Escamilla, et al. 2015 | USA | 12 months | Type 2 Diabetes | Low-income | 211 (105/ 106) | 56.3 (11.8) | 155 (73.5) | Latino/ Hispanic (100) | Mean change in HbA1c (%) | The intervention led to a greater reduction in HbA1c, compared to the control. | P=0.021 |
| ^43^ | Philis-Tsimikas et al., 2011 | USA | 4 months | Type 2 Diabetes | Underinsured, low-income | 207(104/ 103) | 52.2 (9.6) / 49.2 (11.8) | 146 (70.5) | Mexican American (100) | Mean change in HbA1c (%) | The intervention group had a significant decrease in HbA1c, from baseline to month 4 (-1.7%, P = 0.001). The control group had a non-significant reduction of -1.1% (P=0.14) | Between group difference not reported. |
| ^44^ | Protheroe et al., 2016 | UK | 7 months | Type 2 Diabetes | Residents from an area of deprivation | 76 (39/37) | 64.7 (11.2) / 61.5 (10.1) | 38 (50) | NP | Mean change in HbA1c values | No difference in HbA1c change between the groups. | P=0.183 |
| ^45^ | Pyatak et al., 2018 | USA | 6 months | Type 1 and 2 Diabetes | Low income/ education -self-reported household income was below 250% of the federal poverty level or neither parent had a bachelor’s degree. | 81 (41/40) | 22.6 (3.5) | 51 (63) | Hispanic/Latino (78) | Mean change in HbA1c (%) | The intervention group had greater improvement in HbA1c compared to the control group | P=0.01 |
| ^46^ | Rosal et al., 2005 | USA | 6 months | Type 2 Diabetes | Low-income | 25 (15/10) | 62.6 (8.6) | 20 (80) | Hispanic/ Latino (100) | Mean change in HbA1c (%) | The HbA1c decrease was larger in the intervention group compared to control. | P=0.005 |
| ^47^ | Rosal et al., 2011 | USA | 12 months | Type 2 Diabetes | Low-income | 252 (124/128) | NP | 93 (76.6) | Hispanic/ Latino- Puerto Rico (87.7) | Mean change in HbA1c (%) | The intervention effect was not significant. | P>0.293 |
| ^48^ | Ruggiero et al., 2014 | USA | 12 months | Type 2 Diabetes | Low-income | 266 (134/132) | 53.15 (12.36) | 183 (68.8) | African American (52.6) and Hispanic/ Latino (47.4) | Mean change in HbA1c (%) | No intervention effect was found, and no differences were found for A1C. | NP |
| ^49^ | Schillinger et al., 2009 | USA | 12 months | Type 2 Diabetes | Low-income and underinsured | 339 (113/112/114) | 56.1 (12.0) | 200 (59.0) | Multi-ethnic | Patient assessment of chronic illness care (PACIC) | Both the intervention groups showed a greater improvement in PACIC compared to control. | For ATSM: P<0.0001 For GMV: P=0.04 |
| ^50^ | Schoenberg et al., 2017 | USA | 7 months | Type 2 Diabetes | Area of poverty | 41 (20/21) | 58.24 (10.77) | 30 (65.85) | Anglo-white (100) | Mean change in HbA1c (%) | There was no overall difference in HbA1c change over time. | P=0.22 |
| ^51^ | Seligman et al., 2018 | USA | 6 months | Type 2 Diabetes | Food insecure- food bank recipients | 568 (285/283) | 54.8 (11.4) | 384 (68.3) | Hispanic/ Latino (52.1) | Risk difference in mean HbA1c (%) at follow up | No evidence of a difference in HbA1c at follow up. | P=0.16 |
| ^52^ | Shea et al., 2006 | USA | 12 months | Type 2 Diabetes | Medicare beneficiaries | 1665 (844/821) | 70.82 (6.63) | 1040 (62.82) | Multi-ethnic | Mean change in HbA1c (%) | The intervention group had a greater reduction in mean HbA1c level compared to control. | P=0.006 |
| ^53^ | Sixta and Ostwald, 2008 | USA | 6 months | Type 2 Diabetes | Low-income | 131 (63/68) | 56.3 | 93 (71) | Mexican American | Mean change in HbA1c (%)* | There was no difference in the HbA1c level over the study period, within both the intervention and control group. | NP |
| ^54^ | Skelly et al., 2009 | USA | 9 months | Type 2 Diabetes | Rural, low-income | 180 (60/60/60) | 67 | 180 (100) | African American (100) | Mean change in HbA1c (%) | There were no differences in the amount of decline between the 3 study arms. | NP |
| ^55^ | Spencer et al., 2018 | USA | 18 months | Type 2 Diabetes | Area of deprivation residents | 222(60/89/73) | 48.9 (10.6) | 135 (60.8) | Latino | Mean change in HbA1c (%) | From 6 to 12 months, improvements in HbA1c were sustained for participants randomized to the enhanced intervention group (n=60) (-0.63% [95% CIs : -1.06 to -0.19]; P<0.01) but not the regular intervention or the control groups. | NP |
| ^56^ | Talavera et al., 2021 | USA | 6 months | Type 2 Diabetes | Low-education and low-income | 456 (225/231) | 55.72 (9.82) | 290 (63.7) | Hispanic/Latino (96.5) | Mean change in HbA1c (%) | The group by time interaction effect (-0.32, 95% CI: -0.49 to -0.15), indicated greater improvement in HbA1c level over 6 months in the intervention group compared to control. | P<0 .01 |
| ^57^ | Thom et al., 2013 | USA | 6 months | Type 2 Diabetes | Low-income | 299 (148/151) | 55 | 156 (52.2) | multi-ethnic | Mean change in HbA1c (%) | Patients in the intervention group had a 0.77% greater decrease in HbA1c levels at 6 months compared to control | P=0 .01 |
| ^58^ | Wang et al., 2018 | USA | 6 months | Type 2 Diabetes | Low-income, underinsured, and uninsured | 26 (11/9/6) | 56.4 | 16 (62) | African American/ Black (65.38) | Mean change in HbA1c (%) | At 6 months, there were no statistically significant group differences in HbA1c level change. | P=0.44 |
| ^59^ | Wayne et al., 2015 | Canada | 6 months | Type 2 Diabetes | Low-income | 97 (48/49) | 53.2 (11.3) | 70 (72) | Black-Caribbean (40) | Mean change in HbA1c (%) | There was no between group differences in mean HbA1c change from baseline to 6 months | P= 0.48 |
| ^60^ | Whittemore-2020 | Mexico/ USA | 6 months | Type 2 Diabetes | Low-income | 47 (26/21) | 55.35 (8.75) | 31 (68) | Hispanic/ Latino | Mean change in HbA1c (%) | There was little difference of changes between the groups. | P= 0.11 |
| ^61^ | Aikens et al., 2022 | USA | 12 months | Depression | Low-income | 204 (108/96) | 48.6 (12.2) | 165 (80.8) | Caucasian (74.1) | Depressive symptom severity (Patient Health Questionnaire 9) | The intervention group's mean PHQ-9 total had a greater reduction compared to the control. | P=0.004 |
| ^62^ | Apter et al., 2019 | USA | 12 months | Asthma | Area of deprivation: Residents of a neighbourhood in which 20% of households had incomes of less than the federal poverty level. | 301(151/150) | 49 ( 13) | 270 (89.7) | African American (75.4) | mean difference in Asthma Control Questionnaire score | The intervention had greater reduction in ACQ score, but the difference was not statistically significant. | NP |
| ^63^ | Krieger et al., 2015 | USA | 12 months | Asthma | Low-income: household income of less than 250% of the federal poverty level (2007). | 366 (177/ 189) | 41.3 | 268 (73.2) | Multi-ethnic | "symptom-free days" over two weeks. | The intervention group had significantly greater and clinically meaningful increases in symptom-free days compared to control. | P<0.001 |
| ^64^ | Martin et al., 2009 | USA | 3 months | Asthma | low-income | 42 (20/22) | 33 (9) vs 37 (8) | 29 (69.05) | African American (92.86) | Asthma self-efficacy score | Self-efficacy increased in the intervention group and either remained the same or decreased in the control group, controlling for baseline variables. | P< 0.001 |
| ^65^ | Young et al., 2012 | USA | 6 months | Asthma | Income less than or equal to 200% of the federal poverty level. | 98 (49/49) | 44.6 (15.8) | 75 (76.5) | White (92.9%) | Patients' asthma control (Asthma Control Test (ACT)) | Results did not indicate a significant difference between the control and intervention groups. | NP |
| ^66^ | Evans-Hudnall et al., 2014 | USA | 4 weeks | Stroke | Low income and education and underinsured | 52 (27/25) | 56.03 (9.9)/ 46.95 (10.74) | 20 (38.5%) | African American (57) | Tobacco use (Behavioral Surveillance Survey [BRFSS]) | There was a greater proportion of patients with treatment compliant tobacco use in the intervention group compared to control. | P= 0.01 |
| ^67^ | Kronish et al., 2014 | USA | 6 months | Stroke | low-income | 600 (301/299) | 63 (11) | 354 (59) | Multi-ethnic: Hispanic/Latino and African American (86) | Proportion of sample who achieved a composite outcome of control of blood pressure lipids and regular use of antithrombotic medication. | There was no difference in the proportion of intervention and control participants who at 6 months had attained their composite control measure. | P=0.98 |
| ^68^ | Tiliakos et al., 2013 | USA | 6 months | Rheumatoid Arthritis | Low-income | 104 (52/52) | 53.55 | 82 (79) | African American (90) | Proportion of patients who achieved 20 % improvement from baseline according to the American College of Rheumatology (ACR20). | The test for interaction between intervention group and time was not statistically significant. | P=0.7 |
| ^69^ | Eakin et al., 2007 | USA | 6 months | Two or more chronic conditions | Low income | 200 (101/99) | 50 (13)/ 49 (13) | 157 (78.5%) | Hispanic/ Latino (80.2/1.1) | Dietary behaviour (Kristal Fat and Fibre Behavior Questionnaire [FFB]). | The intervention group showed a significantly greater improvement in dietary behaviour compared to the control group. | Very P=0.003 |
| ^70^ | Kangovi et al., 2017 | USA | 6 months | Two or more chronic conditions | Area with high poverty rate, underinsured/ publicly insured | 302 (150/152) | 56.3 (13.1) | 228 (75.5) | African American (94.7) | Mean change in score of participants chosen parameter (HbA1c, BMI, SBP, and number of cigarettes per day) | There were positive differences in the 6-month change in chronic disease parameters, favouring the intervention arm. | P=0.08 |
| ^71^ | Kennedy et al., 2013 | UK | 12 months | Irritable bowel syndrome, Chronic obstructive pulmonary disease, or Type 2 diabetes. | Area of high deprivation | 5599 (2295/ 3304) | NP | 2990 (53.5) | White (96.7) | Change in shared decision making (health care climate questionnaire) | There was no difference between the groups . | P=0.66 |
| ^72^ | McKee et al., 2011 | USA | 6 months | Hypertension and Type 2 Diabetes | low-income | 55 (31/ 24) | 61.2 (11.2)/ 58.6 (7.9) | 36 (65.45) | Hispanic/ Latino (72.73) | Change in proportion at goal for HbA1c (≤ 7%)* | A significantly larger proportion of the intervention group was at goal for HbA1c compared to control. | P=0.049 |
| ^73^ | Mercer et al., 2016 | UK | 12 months | Two or more chronic conditions | socioeconomically deprived area (Scottish Index of Multiple Deprivation) | 152 (76/76) | 52 | 85 (55.92) | NP | Mean change in patient-reported health-related quality of life (EQ-5D-5L). | Positive improvements in quality of life favoured the intervention group at 12 months. However, the overall effect size was not significant. | P=0.15 |
| ^74^ | Riley et al., 2001 | USA | 1 month | 1 or more Chronic diseases | low-income | 28 (15/13) | 58 (9.5) | 23 (82) | Anglo-white (55) | Use of social-environmental resources (Chronic Illness Resources Survey [CIRS]) | The intervention group had increased their use of social-environmental resources significantly more than those in the control group. | P<0.03 |
| ^75^ | Swerissen et al. 2006 | Australia | 6 months | Chronic diseases (general) | Low-income | 474( 320//154) | 66 (9.52) | 355 (74.9) | Multi-ethnic (Greek, Vietnamese, Chinese, Italian) | Health status (self-rated health) | At 6 months, the intervention group had a better mean self-rated health score compared to control | P=0.000 |
| ^76^ | Willard-grace-2015 | USA | 12 months | Hypertension and/or Hyperlipidaemia and/or Diabetes | low income, uninsured or publicly insured | 441 (224/217) | 52.7 (11.1) | 244 (55.3) | Latino/ Hispanic (70.1) | Composite clinical outcome measure- proportion of treatment group with improvement in either HbA1C, SBP, or LDL according to predefined thresholds. | Participants in the intervention arm were more likely than those in the control group to achieve the primary composite measure. | P=0.02 |

Supplementary table 4: Intervention details

| ***Author, date*** | ***Theory*** | ***Materials and Procedures*** | ***Intervention provider(s)*** | ***Mode(s) of delivery*** | ***Setting(s)*** | ***Frequency*** | ***low SES tailoring*** | ***Planned Fidelity assessment*** | ***Actual fidelity*** | ***Financial incentives*** |
| --- | --- | --- | --- | --- | --- | --- | --- | --- | --- | --- |
| Anderson et al., 2010 | No | Patients received unscripted phone calls on disease management followed by mailed educational materials. | Nurses were trained by a "master trainer" who was an expert in commercial disease self-management | Telephone: individual | Centralised call centre | If HbA1c >9, weekly calls, 7< HbA1c <9 or HbA1c < 7 with HTN/ depression/ retinopathy/ neuropathy bi-weekly calls and HbA1c < 7 monthly calls. | Educational materials were available in English and Spanish and at fourth grade reading level | Nurses documented phone encounters on patients’ electronic heath record. Intervention fidelity monitored through chart review by project co-ordinator | NP | $25 gift card to a local store after completing their 6-and 12-month assessments. |
|  |  |  |  |  |  |  |  |  |  |  |
|  |  |  |  |  |  |  |  |  |  |  |
| Arora et al., 2013 | No | Participants received unidirectional, SMS text messages sent. Text messages were based on content from the National Diabetes Education Program. | Automated | Text messages: individual | Remote | Two daily text messages (9am and 5pm) over 6 months. Each text is a 160 character phrase. | Texts were available in English and Spanish at fifth grade reading level. If needed patients were financially compensated ($20 per month) to upgrade to an unlimited messaging plan on the phones. | No | NP | $175 during 6 months for time and travel costs associated with study follow-up visits. |
| Baig et al., 2015 | Self-determination theory, Social cognitive theory and the Transtheoretical model (stages of change) | Patients received a faith based diabetes self-management education program (DSME). | Trained, lay leaders, who either had diabetes themselves or knew a friend or family member with diabetes. Lay leaders underwent three 3-hour training sessions on coaching skills through modelling, program content, feedback, and role play. | Face to face: group | Churches | Eight sessions weekly, 90 minutes each. | The DSME was faith based and culturally tailored. Lay leaders were bilingual in English and Spanish. | Members of the academic team observed the class leaders during the first eight-week class and then periodically to ensure intervention fidelity using standard processes including checklists and direct observation. | NP | No |
| Berry et al., 2016 | no | Patients received group diabetes self-management education. | Health nurse practitioner, a physician, a postdoctoral fellow, and a trained interventionist | Face to face: group | Community health centre | Five sessions- one session every three months. | np | np | np | No |
| Chamany et al., 2015 | Self-efficacy theory and the Transtheoretical model (stages of change). | Patients were mailed a “welcome” packet that included print materials on diabetes self-management and healthy retention incentives such as pedometers. Also they received self-management support via telephone. | Health educators who received 20 hours of training in delivering behavioural counselling by phone. Health educators also attended a 10-hour American Diabetes Association–recognized diabetes self-management program. They were supervised by a team consisting of a nurse-certified diabetes educator, internal medicine physician and clinical health psychologist thoroughly weekly case meetings. | Telephone: individual | Remote | Four calls (one every three months) over 12 months if baseline HbA1c was in the >7.0% and <9.0%, or eight calls over 12 months if HbA1c was >9.0%. | The health educators were bilingual in English and Spanish, and the print materials were adapted for low literacy. | Fidelity to the protocol was enhanced by the use of telephone log sheets for documenting details of every call. Also, every study participant had a protocol flow sheet with exact dates by which protocol activities had to occur | np | No |
| Clancy et al., 2007 | No | Patients received group medical visits. | The groups were co-led by an internal medicine physician and a registered nurse, modelling the format of Cooperative Health Care Clinics (CHCC). They were trained by a senior internist who had previous experience in group visits. Also, the previous trainer for CHCC providers, gave a 3 hour educational training session to clinic staff. | Face to face: group | Clinic | 2 hour group sessions, delivered monthly over 12 months. | No | No | NP | A visit deposit fee per visit of $15 for intervention patients and $45 for control group patients. |
| Davis et al., 2010 | Health Belief Model and Transtheoretical Model (stages of change) | Patients received remote DSME, with content based off the "Pounds Off With Empowerment" materials, the ADA (American Diabetes Association guidelines) and the Michigan Diabetes Research and Training Centre’s Life with Diabetes Curriculum. | A self-management education team, consisting of a nurse/ certified diabetes educator and a dietitian. | Face to Face: group and video conferencing: group and individual | Academic health centre (for the providers) and primary care clinic (for the patients) | Over 12 months there were 13 session in total, with two being held in the first month (one group and one individually). Sessions were monthly thereafter. 10 sessions were group based, and the remaining three were individual. | Modifications included considerations for a low-literacy and a rural population. | No | NP | Participants were given a gift card for each of the three completed visits. |
| Fitzpatrick et al., 2022 | No | Patients received resource navigation in addition to a problem-solving based, ADA recognised DSME programme. | Community health workers (CHWs) who were already embedded in culturally specific community-based organisations. CHWs received 20 hours of training in diabetes, delivering the DSME programme and addressing social needs. | Face to Face and telephone: individual | Patients' homes or community settings (churches, cafes, libraries) | 9 modules were delivered over the 6 months on a weekly and bi-weekly basis. Resource navigation support was provided as needed. | The DSME curriculum was adapted for low literacy. All materials were available in English and Spanish and the CHWs were ethnically diverse. | A random selection of CHW visits were audio recorded and reviewed as a check for fidelity. | NP | Participants were given a $50 gift card for completing the 6-month follow-up. |
| Fortmann al., 2017 | No | Patients received an m-health SMS text based self-management intervention. Text message content was based on the Project Dulce DSME curriculum. The text bank included 119 different messages, less than 160 characters in length. | Bilingual study co-ordinator | Text messages: individual | Remote- texts were sent out via a contracted patient health management technology platform. | At the start of the intervention period, texts were 2-3 times pay day at standardised hours. Frequency tapered off as the study progressed. | Patients who did not have a mobile-phone with texting capability were provided free of charge. Those with their own phones had the costs of the additional texts covered by the study ($12/month). Texts were in English and Spanish. | No | NP | Participants received incentives at baseline, 3-months and 6-months assessment |
| Frosch et al., 2011 | No | Patients received one 24-minute-long DVD program with an accompanying booklet called "Living with Diabetes: Making Lifestyle Changes to Last a Lifetime", which was developed by the Foundation for Informed Medical Decision making. Patients could also receive additional telephone support. | A nurse educator trained in patient-centred approaches to diabetes management and motivational enhancement. | Mail and telephone: individual. | Patients receive the calls and material remotely, from their homes. | Five phone calls in total. Call one being up to 60 minutes. Calls two and three up to 30 minutes. Calls four and five up to 15 minutes. Patients could receive no more than 150 minutes (2.5 hours) worth of telephone support. The time interval between calls was at the discretion of the patients and nurse educator. | The nurse educator was bilingual in English and Spanish. | To improve fidelity, patients received a call one week after enrolment in the study to remind them to review the intervention materials provided and again to schedule a telephone session. Fidelity was assessed as the number of phone sessions each patient underwent. | 73.0% completed five sessions of telephone coaching. The mean (SD) number of sessions completed was 4.0 (1.9). | No |
| Gary et al., 2009 | PRECEED-PROCEED Framework | Patients received individualised care and self-management support, in the form of intervention action plans (IAPs) based on a clinical algorithm. | Both nurse case managers (NCMs) ad community health workers (CHWs) received 6 weeks’ worth of training. CHWs continued to have weekly meetings with the project co-ordinator to reinforce initial training and go over any problems. | Face to face and telephone: individual. | Clinic, patients' homes, community settings or remote. | NCMs conduct a minimum of one face to face clinic visit per patient per year. CHWs conducted home visits at least 3 times a year. However, the frequency and intensity of the intervention for each patient is guided by the algorithm which triages them according the diabetes control level. For example, those with "poor control" will receive weekly contact versus every other week for those with optimal control. | CHWs are also African American. | No | NP | No |
| Greenhalgh et al., 2011 | No | Participants took part in a semi-structured, informal group story-telling intervention, each session based around themes. Participants shared the experiences self-managing diabetes as it related to the group-selected theme. | The story telling facilitator (bilingual health advocate [BHA]) was a non-clinical professional or volunteer trained in the sharing stories model. Medical professionals such as a dietician, exercise specialist or diabetes nurse were invited to one-off sessions on a case by case basis. | Face to face: group | Informal community settings | Each session lasted 2 hours and was held every 2 weeks for 6 months. | BHAs were bilingual, groups were offered in Bengali, Tamil, Punjabi, Urdu, Gujaratis and English. Those with mobility needs were offered minicab transport, allowing ‘housebound’ patients the opportunity to join the study. | A researcher attended all but 8 of the 72 story-sharing sessions and checked that they followed the established protocol and format. | NP | No |
| Hill-briggs et al., 2011 | D’Zurilla and Nezu problem-solving therapy | Patients received a diabetes and CVD education session and problem-solving training sessions. Patients also received two workbooks: "Diabetes and Your Heart Facts & Information Workbook" and "Hitting the Targets for Diabetes and Your Heart: Your Problem-Solving Workbook". | The interventionists underwent training and followed prepared manuals. | Face to face: group | NP | One education session and 8 problem-solving training sessions, each lasting 90 minutes, delivered biweekly. | The sessions and materials (workbooks) were adapted for accessibility and usability in low literacy and functionally impaired populations. The workbooks made use of colours (red vs green) and symbols to simplify concepts. | All sessions were audiotaped, and randomly selected audiotapes were reviewed | NP | No |
| Lynch et al., 2014 | Information processing model. | Participants received a community-based, group intervention that focused on diet and physical activity, and follow up peer support. | Classes were facilitated by a registered dietitian, who was assisted by two peer supporters. Peer supporters trained weekly for 8 weeks (2 hours per week) with a psychologist, dietitian, and health educator. Training sessions familiarised the peer supporters with goal setting and the nutrition education materials. | Face to face: group and telephone: individual. | Local city park building near the recruitment clinic. | 18, 2-hour LIFE classes, which were weekly for the first 3 months and every other week for the second 3 months. Telephone support was weekly. | Peer supporter were also African American and had diabetes or hypertension. They came from the same community as the participants. | No | NP | No |
| lynch et al., 2018 | Cognitive behavioural models of behaviour change and Information processing model | Participants received group based DSME and individualised peer support. The bulk of the LIFE intervention curriculum focused on diet change and goals. It was based on a modified plate method referred to as, "the Plate of LIFE." Participants also received educational materials and workbooks. | The intervention team for each group session consisted of a registered dietitian, a group facilitator, and 1-2 peer supporters. A clinical psychologist supervised peer supporters. Peer supporters completed 8 hours of training. They were trained to reinforce progress on goals with verbal praise and apply simplified motivational interviewing and problem solving techniques. Peer supporters provided telephone support. | Face to face: group and telephone: individual. | Community settings near the main clinic. | 28, 2-hour group sessions over 12 months: weekly for the first 4 months, biweekly for the second 4 months, and monthly for the third 4 months. Two additional maintenance sessions were held at months 15 and 18. Telephone support was delivered at the same frequency. | Peer supporters were also African American and the curriculum was culturally tailored. To address literacy barriers the sessions and materials made use of graphics, simplified food lists, and physical demonstrations and hands-on activities to reinforce more abstract concepts. Numeracy barriers were addressed by repeated visual and tangible exercises counting out carb portions (using real food). | Yes: Fidelity was monitored using checklists developed for each session to assess content delivery. Group sessions were recorded using a digital voice recorder. The project director reviewed fidelity data and provided feedback. | NP | US$100 for each of the three full assessment and $25 for brief assessments. |
| Nelson et al., 2017 | Self-efficacy theory | Patients received home visits where their current diabetes self-management was assessed using a structured interview. Community health workers (CHWs) worked with participants to set health goals and develop an action plan. Participants also received complimentary educational materials. | Community health workers (CHWs) who received 60 hours of mandatory training in health coaching and motivational interviewing by a professional health coach and training in how to use a blood pressure monitor. Each CHW passed a competency test prior to intervention delivery. | Face to face: individual | Participants' homes | 4 mandatory home visits that took place 0.5, 1.5, 3.5, and 7 months after enrolment. There was a fifth optional visit at month 10. | The CHWs were bilingual in English and Spanish and educational materials were available in both languages. Materials were also adapted for those with low literacy. | Yes: CHWs completed an encounter form after each visit. The forms were reviewed monthly by a certified diabetes educator to ensure that each participant receives the required components of the intervention. | NP | US$25 at both baseline and 12 month assessment |
| Pérez-Escamilla, et al. 2015 | Transtheoretical model (stages of change) and Problem solving theory | Participants received home visits were they were taught the DIALBEST curriculum in modules and received a tailored self-management plan. | Community healthcare workers (CHWs) (nurse and medical assistant) who were employed by a community based non-profit organisation. They received 65 hours of core training and 25 hours of supplementary training by an interdisciplinary team of academics and practitioners in topics such as diabetes pathology, lifestyle strategies for glycaemic control, motivational interviewing, communication skills and social determinants of health. | Face to face: individual | Participants' homes | 17 visits over a 12 month period. Visits were weekly during the first month, biweekly during months 2 and 3, and monthly until month 12. | CHWs were bicultural/ bilingual in English and Spanish. The curriculum was designed to be both culturally and health literacy appropriate. The self-management plans were individually tailored to meet the participants' socioeconomic circumstances. | Yes: An ancillary study was conducted to audit the CHW progress notes and phone records to document intervention fidelity. | Over half of the participants (51%) received the scheduled 17 visits, with an average duration of 87.8 (18.2) minutes per home visit. | no |
| Philis-Tsimikas et al., 2011 | No | Participants received diabetes self-management education based on the Project Dulce "Diabetes among friends" curriculum. | Peer educators (PE) known as "promotoras". They were individuals with diabetes, identified as "natural leaders" from a patient population. Over a 3-month period they received 40 hours of training in the curriculum, group instruction, mediation, and behaviour change techniques. | Face to face: group and telephone: individual | NP | 8, 2-hour group classes, delivered weekly and then monthly support groups. Telephone contact was made before each class to increase attendance. | PEs were bilingual in English and Spanish | Yes: To ensure the fidelity of intervention delivery, all classes were audio recorded and reviewed using checklists to monitor the delivery or omission of curriculum component. | NP | Yes: participants were given small gift cards at each of the three assessment point (amount not disclosed). |
| Protheroe et al., 2016 | No | Patients received an individualised self-management plan, following an interview with a lay health trainer, along with follow up telephone support and a printed pamphlet. The interview identified areas for improvement in their health. | Lay health trainers (LHT) received training from the research team on evidence based diabetes care and appropriate lifestyle advice. | Face to face and telephone: individual. | NP and remote | 1 interview took place at the start of the intervention period, followed by up to 3 2-monthly support phone calls. | Pamphlets were adapted for low health literacy | No | NP | No |
| Pyatak et al., 2018 | Social-ecological model of health behaviour and Complexity Theory | Participants received an adaptation of the Lifestyle Redesign OT intervention framework, which involves a manualised, individually tailored intervention, composed of 7 flexible modules. | Two licensed occupational therapists (OTs) who received 20 hours of training in the intervention manual, 12 hours of training in motivational interviewing and 20 hours training in diabetes self-management education. An endocrinologist and a licensed clinical social worker were available on an as-needed basis for issues identified which were outside the main scope of the intervention. | Face to face: individual | Participants' homes and community settings. | 12 biweekly sessions averaging one hour each, over 6 months. Timings were flexible; however, the aim was to deliver an intervention dose of between 10-16 hours per participant. | NP | Intervention fidelity was maintained through three strategies. First, therapists documented intervention dose, timing, and treatment activities in notes completed after each session. Second, approximately 10% of sessions were observed by a second therapist trained in the intervention protocol, who completed a fidelity checklist and shared feedback with the treating therapist. Third, all team members trained in the intervention met weekly to facilitate problem-solving and prevent intervention drift. | Fidelity monitoring showed that the therapists had 96% adherence to the intervention’s key components. | Yes received US$25 at baseline and US$50 at follow-up. |
| Rosal et al., 2005 | Social cognitive theory | Participants received an interactive self-management education program. The program involved: direct instruction and modelling through a soap opera, skill-building activities, personalised goal setting and skill reinforcement activities. | A nutritionist, nurse, and intervention assistant were trained in the intervention’s theoretical and delivery models, intervention goals, counselling skills, and use of materials. | Face to face: individual and group | A community room 3 block from the health centre and two blocks away from the elder service. | One initial 1-hour individual session, followed by 10 weekly 2.5 to 3-hour group sessions and two 15-minute individual sessions that occurred during the 10-week period immediately prior to the group session | The intervention team was bilingual and the interventions used the traffic light concept and visual aids as a means of simplifying educational messages. | No | NP | Participants were offered incentives equivalent to US$90 for completing the assessment spread out over the 3 assessment points. |
| Rosal et al., 2011 | Social cognitive theory | Participants received an interactive self-management education program. The program involved: direct instruction and modelling through a soap opera, skill-building activities, personalised goal-setting and skill reinforcement activities. | The intervention delivery team consisted of two leaders and an assistant (either a nutritionist or health educator and trained lay individuals or three lay individuals). The intervention staff received approximately 40 hours of extensive training in accordance with a protocol that covered diabetes self-management, the theoretical foundation of the intervention and group management skills. | Face to face: individual and group | Participants home (first individual session only) and community settings such as senior centres and local YMCAs. | one 1-hour individual session followed by 11 weekly 2.5-hour group sessions and 8 monthly group sessions. Each group session included a 10-minute one-to-one session for each participant with one of the intervention team. | The intervention was culturally, and language tailored (English and Spanish). The content was adapted for low literacy by simplifying concepts, minimising didactic instruction and using picture and colour-coded based guides. | Yes: Fidelity checklists monitored delivery or omissions of intervention components. Supervision of interventionists included a review of completed checklists following the sessions. | NP | No |
| Ruggiero et al., 2014 | Transtheoretical model (stages of change) and empowerment theory | Patients received a self-care coaching intervention. The aim was to help the patients learn the necessary information and skills to make informed self-care goals and changes, using the 5As framework and motivational interviewing as the primary coaching methods. They were also provided with written materials matched to their stage of change in the framework. | Medical assistants served as medical assistant coaches (MACs). In addition to the standard medical assistant training, they received more than 40 hours of initial project training and ongoing boosters. They were trained by the multidisciplinary team in diabetes self-management, behavioral counselling strategies guided by the theory, motivational interviewing and the 5As framework. | Face to face and telephone: individual. | The clinic and Remote | Face to face clinic visits were delivered quarterly during routine diabetes visits at the clinic and were less than 30 minutes in length. Telephone follow-ups were monthly and less than 15 minutes in length. | MACs were of the same ethnicity (African American or Hispanic/ Latino) as the patients at their clinic, educational materials were culturally tailored, written at a fifth grade or below reading level and were available in both English and Spanish. | Yes: The PI and project coordinator reviewed and tracked intervention reports, notes, and charts. There was occasional direct observation by a trained research assistant and periodic PI observation of the ongoing training. | The majority of patients did not receive the intended dose of the intervention, | US$20 cash for baseline assessment and US$25 cash for the two follow-up assessments |
| Schillinger et al., 2009 | Self-efficacy theory | Patients received individual action plans and took part in collaborative goal setting. This was achieved through an Automated Telephone Self-Management Support System (ATSM), where patients responded to automated queries regarding their self-care. Action plans and interactions are linked to the patients' clinic record. | IDEALL clinical staff, including the nurse diabetes care managers for the ATSM, were trained in model protocols, motivational interviewing, and communication techniques for patients with limited literacy. | telephone: individual | Remote- patients received calls in their homes | Weekly calls over 39 weeks (9 months). Each call takes between 6-10 minutes to complete. | Intervention was delivered in English, Spanish, or Cantonese. | No | NP | US$15 AND US$25 at baseline and 1-year follow up respectively. No additional incentive to answer calls. |
|  |  | Patients received individual action plans and took part in collaborative goal setting. This was achieved through Group Medical Visits (GMV). The visits involved discussing concerns, problems or progress with the plans and modelling of self-management behaviours. | IDEALL clinical staff for GMV included a physician, pharmacist, and health educator. In addition to the training described above they received training in group facilitation techniques. | Face to face: group | NP | Monthly visits of 9 months. Each lasting approximately 90 minutes. | Intervention was delivered in English, Spanish, or Cantonese. Participants were given bus tokens to assist with transportation costs. | No | NP | US$15 AND US$25 at baseline and 1-year follow up respectively. |
| Schoenberg et al., 2017 | No | Patients received a hybrid model of diabetes self-management classes (with goal setting) combined with care navigation based on the chronic care model. | Trained Community Health Workers (CHWs). | Face to face: group | Field office | The 6 classes took place every 2 or 3 weeks. | The classes were culturally appropriate and material was delivered at a fifth grade or below reading level. | No | NP | US$25 at both baseline and follow up assessment. |
| Seligman et al., 2018 | No | Patients received food packages and DSME modelled on the American Association of Diabetes Educators AADE7 Self-Care Behaviours and adapted from components of the Type 2 Diabetes BASICS curriculum. | Food bank staff, volunteers, and health educators. Educators were food bank staff trained in curriculum delivery by a registered nurse and diabetes educator. Staff also received training in the following subjects: diabetes pathophysiology, screening, evaluation, and treatment, client privacy and HIPAA regulations, universal precautions and sharps safety, medical waste handling and use of specific study equipment. | Face to face: group | Food bank | 2 mandatory group classes in the first 2 months of the intervention period, each lasting between 2 to 2.5 hours. Optional "drop-in" monthly sessions were available for the next 4 months and were 60 to 90 minutes long. Participants could receive 11 food packages picked up twice monthly over the 6 month intervention period. | Classes and material were available in English and Spanish. The DSME curriculum and intervention was tailored to address literacy, numeracy, transportation barriers and costs, food-access barriers, and food insecurity. | No | No | US$15 gift cards at each assessment |
| Shea et al., 20006 | Social cognitive theory | Patients received telehealth case management via a home telemedicine unit (HTU), which consisted of a web-enabled computer with video conferencing capabilities. | Nurse case managers and dieticians conducted the tele-health visits | HTU video: individual | Remote | Every 4-6 weeks across a 5 year period. | The intervention providers for ethnic minority patients were bilingual in English and Spanish and were Hispanic/Latino or African American so advice could be tailored to the patients cultural background. | No | NP | No |
| Sixta and Ostwald, 2008 | No | Patients received a diabetes self-management course, according to a scripted course curriculum | Promotores, employed by the clinic, led the course sessions in pairs, supervised by nurses. The nursing director oversaw quality control and promotores' education and training. | Face to Face: group | Community clinic | 10, 1.5 hour sessions held weekly. | The course curriculum was presented in Spanish, was culturally sensitive and used pictures to aid understanding. | No | NP | No |
| Skelly et al., 2009 | No | Patients received a symptom focused diabetes intervention (teaching and counselling) based on the University of California, San-Francisco symptom management model . Half of patients also received a telephone booster, reinforcing the content and strategies of the home visit. | Nurses | Face to face and telephone: individual | Patient homes and remote | The 4 home visits were 60 minutes and took place bimonthly. The 4 telephone boosters took place 3 months after the last visit and occurred every 2-3 weeks. Each call averaged 15 minutes in length. | The teaching was individualised and made specific to each patient home and community. Also, the visits took place at the patients' homes to avoid transport related barriers. | No | NP | No |
| Spencer et al., 2018 | Social cognitive theory | Participants received an empowerment based group diabetes self-management education (DSME) classes, based on the Racial and Ethnic Approaches to Community Health (REACH) curriculum for Latinos. In addition, they received home visits and accompanied clinic visits. | Community health workers (CHW), who underwent more than 160 hours of CHW training, more than 80 hours of diabetes education, including home visit experiences, training in human subjects protocols, behaviour modification strategies, cultural competency, and community-based participatory research. | Face to Face: group and individual | Community locations, participants homes and clinics and remote | 11 2-hour group DSME sessions held every 2 weeks, 2 60 minute home visits each month and 1 accompanied clinic visit over a 6 month period. | The CHWs were Latinas, bilingual in English and Spanish and were from the same community/ area as the participants. | No | NP | No |
|  |  | After the initial 6 month CHW intervention patients could receive ongoing emotional and behavioral support. | Peer leaders (PLs) recruited by the CHWs. They received 46 hours of training over 12-weeks and monthly booster sessions over the 12 month intervention period | Face to face: group and telephone: individual. | Community locations and remote. | Group drop-in sessions held weekly over a 12 month period. PLs made calls to any participants who had not attended three sessions in a row. | Peer leaders were from the same community as participants but had already done the DSME curriculum previously. | No | No | No |
| Talavera et al., 2021 | No | Patients received a team based integrated care and behavioural intervention based on the 5As framework. It consisted of a medical visit, behaviour visit, group DSME classes and care co-ordination. The DSME class curriculum materials were developed as an adaptation of the Pasos Adelante/Steps Forward intervention. | The team consisted of a physician/ medical provider, a specialty behaviour health provider and a peer health educators. | Face to face: individual (medical and behaviour visits) and group (self-management classes) | The partnership clinic | 4 medical and behaviour visits over a 6 month period and 6 2 hour DSME self-management classes. | All intervention providers were bilingual in English and Spanish and Latino. DSME classes emphasised visuals and minimal text to accommodate varied  levels of literacy. | Yes: The number of medical and behavioural visits and DSME classes were tracked. DSME classes were audiotape recorded and reviewed by a trained research assistant who used a checklist to evaluate coverage of key content and ensure delivery as intended. The behavioural health providers completed a checklist based on the 5-As framework after each visit. | No major deviations. Fidelity by the 5 As framework showed the following. Assess (99%), Advise (96%), Agree (78%) , Assist (75%-97%, depending on the topic), , and Arrange (79%). 47 participants received no intervention contact. | No |
| 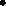 Thom et al., 2013 | No | Patients received peer health coaching. | Peer health coaches who were patients at the clinic, who had an HbA1c level of less than 8.5% within the past 6 months. They received 36 hours of training over 8 weeks, conducted by two of the research investigators. Trainees who passed both a written and an oral examination became peer coaches. Trainees received US $150 for completing the training, regardless of if they passed. | Face to face and telephone: individual. | The public health clinic and remote. | Telephone contact must be at least twice a month and in-person contact at least 2 times during the 6 month intervention period. | Peer coaches were from the same clinic/ community and spoke either English or Spanish. | No | NP | Patients received US $10 after baseline data collection. |
| Wang et al., 2018 | Social learning theory and Self-regulation theory. | In addition to usual diabetes care and education, participants received lifestyle-based intervention sessions and tracked progress using a series of mobile apps (Diabetes Connect app and LoseIt!) | Lifestyle counsellors were trained using publicly available materials and a digital optical disc and printed training materials from the Group Lifestyle Balance (GLB) program and the Look AHEAD intervention | Face to face and mobile: group and individual. | Community centre | 11 group sessions: weekly for month 1, biweekly for months 2 and 3, and monthly for months 4 to 6—and an individual session after month 3. Each session was 1 to 2 hours. | All intervention materials were modified to be at ninth grade reading level. Also, participants without a smartphone were lent one for the study duration. | A checklist was used for each group and individual session to track the content delivered. The principal investigator (PI) attended at least 80% of the group sessions for both paper and mobile groups to ensure treatment fidelity. | NP | No |
|  |  | In addition to usual diabetes care and education, participants received lifestyle-based intervention sessions and tracked progress using CalorieKing food and exercise journals |  |  |  |  | All intervention materials were modified to be at ninth grade reading level. |  |  |  |
| Wayne et al., 2015 | Intervention described as theory driven but details not provided | Participants received health coaching with additional mobile phone support, based on a behaviour change curriculum co-designed by the study authors. The app used was the Connected Wellness Platform (CWP) provided by NexJ Systems, Inc. | Health coaches who were behaviour change counselling specialists, with expertise in chronic disease management. They were either certified exercise physiologists or personal trainers. All coaches attended weekly seminars and meeting prior to and throughout the trial for training in the curriculum. | Mobile app and face to face: individual. | Remote | App communication was 24 hours a day/ 7 days a week basis. | Participants were provided with a Samsung Galaxy Ace II mobile phone during the intervention period. Also the health coaching curriculum was adapted for the socioeconomic context and ethnocultural backgrounds. | No | Mean contact time between participants and health coaches was 38 minutes/week (SD 25) | No |
| Whittemore-2020 | Social cognitive theory, Empowerment theory and Health Action Process Approach Model (HAPA) | Participants received education sessions supplemented by text messages. | The group session coordinators were a registered nurse and social worker who received one week's worth of training. The training program consisted of content on the program and its theoretical underpinnings, the pathophysiology and treatment of type 2 diabetes, and the social determinants of health in Mexico City. | Face to Face: group, text messages and telephone calls: individual | In 5 Seguro Popular clinics | Seven group sessions, which were followed up by a phone call every two weeks and daily text messages. | Texts were written at a third to fourth grade reading level, with simple pictures to enhance understanding. For those unable to receive texts, the text content and pictures were printed on card. The group sessions were made to be culturally relevant and appropriate for adults with low health literacy. Group activities were tailed to the cultural and socioeconomic context of participants. | A 5-item fidelity checklist and attendance were completed by the group session leaders. Also, approximately 35% of sessions were observed by a trained research assistant to ensure protocol fidelity. | Average group session attendance was 89%. 100% of participants received texts at 6 months (96% at 3 months). 88% received picture messages at 6 months (83% at 3 months). | Participants received department store gift cards after each data collection point- $200 Mexican pesos (∼$10USD) at baseline, $300 Mexican pesos (∼$15 USD) at 3 months, and $400 Mexican pesos (∼$20 USD) at 6 months |
| Aikens et al., 2022 | No | Patients and their nomination "care partner" (CP) received depression self-management advice via an automated interactive voice response (IVR) telephone system. | Automated: The structured algorithm determines which pre-recorded queries patients hear. | Telephone: individual | Remote | Over a 12 month period, patients received calls weekly, with each call lasting 5 to 10 minutes. | NP | No | No | After each of the three planned assessment, patients, their CPs and in-home supporters were offered a $50 cash card for attendance. Patients could receive up to $150 during the study. |
| Apter et al., 2019 | No | Patients received one-off training in patient portal use on how to: locate a laboratory test result, check an upcoming doctor’s appointment, schedule an appointment, locate medication lists, find their immunization record, request a prescription refill, and send a secure message. They also received home visits for care-coordination and to promote their online patient portal use and familiarity with health information technology. | Community health workers (CHWs) who were trained as lay health educators. | Face to face: individual | Patients' homes | Four visits over six months at weeks 2-4, 4-7, 6-11 and 23-27. | CHW were from the same community as the patients. | No | NP | No |
|  |  |  |  |  |  |  |  |  |  |  |
|  |  |  |  |  |  |  |  |  |  |  |
|  |  |  |  |  |  |  |  |  |  |  |
| Krieger et al., 2015 | Social-cognitive theory and Self-regulation theory. | Participants received home-based asthma education, support, and service coordination. Through motivational interviewing they developed a tailored asthma self-management plan. | Community health workers (CHWs) with personal experience of asthma. They received 80 hours of classroom training followed by biweekly training sessions. A health educator and nurse provided clinical support and a manager provided oversight. | Face to face, telephone, and email: individual | Patient homes | 1 initial visit/ assessment at baseline, followed by 4 follow-up visits 0.5, 1.5, 3.5, and 7months later. Additional telephone and email support was on an as-needed basis. | CHWs could speak Spanish and English and were from the same community as participants. | The project nurse conducted monthly audits of home visit records. The project manager or nurse observed at least 1 home visit per month per CHW and rated it with a structured tool. | 90% of identified problems on each participant’s asthma problem list were addressed with the correct protocol, 86% of mandatory protocols were discussed, and 83% of active problems were addressed at each visit. | US$35 and US$50 for completing baseline and exit data collection. |
| Martin et al., 2009 | Self-efficacy theory and social learning theory | Patients received group education sessions and home visits and co-developed an asthma self-management plan. Sessions and home visits involved environmental restructuring, problem solving, and asthma related goal setting as mechanisms for improving self-management skills. | A social worker led the group sessions with the support from community health workers (CHWs). CHWs delivered the home visits. CHWs were trained to establish relationships with participants, successfully implement home visits, and teach basic asthma facts, skills, and self-management techniques. The social worker was trained to effectively lead self-management group sessions and to supervise the CHWs. Altogether both the CHWs and social worker received 113 hours of training. CHWs were evaluated by study investigators using a standardized role-play scenario to determine their readiness. | Face to face: individual and group | Primary care clinic (group sessions) and patients' homes. | 4 group sessions (2 hours each) and 4 to 6 CHW home visits over a 12-week period. | NP | CHWs, and investigators met weekly throughout the study implementation phase to review documentation. | CHWs reported covering all the required areas of asthma education, with the most emphasis on controller medications and taking medications correctly. | Participants received US$25 after attending each group session and were mailed US$10 after each home visit. |
| Young et al., 2012 | Self-efficacy theory | Patients receive counselling based off materials from the Indian Health Services’ patient-counselling model and the Pharmacist-patient consultation program, which formed the communication guide. | Trained pharmacists who were certified in the National Asthma Educator Certification Board Exam. Pharmacists were trained by a patient–provider communication expert. | Telephone: individual | Remote- patients received calls in their homes | three phone calls over a three month period | NP | During the intervention, pharmacists were evaluated during the intervention by a health communication scientist to examine their fidelity. Using the standardized counselling framework as a guide, the scientist reviewed and commented on the pharmacists’ adherence to the protocol. | NP | Participants were reimbursed $75 for study participation: $50 at the beginning and $25 at study completion |
| Evans-Hudnall et al., 2014 | No | Patients received self-care sessions, with content based on the AHA (American Heart Association’s) guidelines and the 5As framework and cognitive behavioural therapy (CBT). | A health educator with a bachelor’s degree in health education and several years’ experience conducting chronic illness self-care education sessions. They also received training in stroke-specific health problems and CBT techniques | Face to face and telephone: individual. | Intensive care unit and remotely in patient's homes. | One face-to-face session at the start of the program and two phone calls, one every two weeks, for four weeks. Each session was 30-45 minutes. | The health educator made sure to recommend free and easily accessible resources to aid in the adoption and maintenance of the specified goals. Materials were culturally tailored based on the religiosity and collectivism constructs prevalent amongst African Americas and Hispanics. | No | NP | No |
| Kronish et al., 2014 | No | Participants received a peer-led stroke prevention group–based workshop adapted from the Chronic Disease Self-Management Program. After each session, participants were required to make an action plan with a goal. | Peer leaders who received 5 days of training in the Stanford Program’s philosophy and methods. The trainers observed the first course and 20% of subsequent courses taught by new peer leaders. | Face to face: group | Community settings | 6 weekly workshops, each 90 minutes in length. | Peer leader were from similar socioeconomic backgrounds as the participants. Workshops and materials were available in both English and Spanish. Concepts were taught in terms lay people could understand. | No | NP | No |
| Tiliakos et al., 2013 | No | Patients received an arthritis self-management program (ASMP). The ASMP was supplemented by a printed educational manual. | An instructor. | Face to face: group | The hospital | weekly 2-hour sessions over six weeks. | The education manual was at an eighth grade reading level. | No | NP | No |
| Eakin et al., 2007 | No | Patients received a lifestyle intervention based off of the 5As framework advocated in multiple behavioral risk factor interventions (Ask, Assess, Advise, Agree, Arrange) | An experienced health educator. | Face to Face, telephone, and newsletters: individual | Clinic or patients home for face-to-face visits, depending on patient preference. | Two face-to-face visits lasting 60-90 minutes, three months apart. Three phone calls, two after the first visit (two and six weeks after) and one after the second visit (two weeks after). Three newsletters were also sent. | Tailoring included the use of visual aids for low literacy, cultural adaptations and materials translated into Spanish. Health educators were also bilingual. | Fidelity was assessed by tracking the delivery of the intervention protocol, including the number of intervention sessions delivered, and the percentage of patients who set goals on physical activity and dietary behaviour change. | Of the 101 intervention participants, 48 (47.5%) received two visits, 39 (38.6%) received one, and 14 (13.9%) could not be contacted for visits or calls. 46 (45.5%) received the three follow-up phone calls, 29 (28.7%) received two calls, 9 (8.9%) received one call, and three (3.0%) were never reached for follow up-calls. | No |
| Kangovi et al., 2017 | Goal setting theory | Participants underwent a collaborative goal-setting session, followed by ongoing support. The follow up support included tailored coaching, social support, advocacy, and navigation to community resources. Participants co-developed tailored action plans for their chosen goals with Community health workers. They could also attend group support sessions. | Research assistants and primary care providers provided the collaborative goal-setting and were offered a 60-minutes training session. Community health workers (CHWs) delivered the follow-up support. They underwent a month-long college accredited training course covering topics such as action planning and motivational interviewing. CHWs were supervised by a manager, who was typically a master’s level social worker. | Face to face: group and individual, text and telephone: individual | Primary care clinic (weekly support group), participants homes and community settings. | 1 collaborative goal setting session at baseline. Follow-up support from CHWs was delivered at least once weekly through various forms of communication. The support groups were weekly, but optional over the 6 month period. | Collaborative goal setting made use of visual aids. Each individual plan was developed with the social determinants of health in mind. | Research assistants were observed during an initial training period to asses fidelity to the collaborative goal-setting scripts. Managers assessed fidelity of the CHW support component through a recurring series of weekly assessments such as, chart review, quarterly day-long observation, calls to patients to hear about their experience and a performance dashboard. | Patients and CHWs created an average of 4.6 action plans over the course of their 6-month relationship. These action plans most commonly related to health behaviour changes (58.9%) and psychosocial issues (23.5%) | US$10 pre-paid gift card upon completion of the baseline survey, US$20 upon completion of baseline laboratory testing and US$30 upon completion of the 6-month follow-up assessment |
| Kennedy et al., 2013 | Normalisation Process Theory | Patients received a whole systems model of self-management support compared within routine primary care. The patient-centred approach to the routine management of long-term conditions focuses on providing skills, resources, and motivation to patients. | Primary care providers (doctors, nurses, technicians) received two days of training by two facilitators. The training teaches three core skills: 1. Assessment of the individual patient’s self-management support needs, in terms of their current capabilities and current illness trajectory. 2, Shared decision making using the PRISMS (Patient Report Informing Self-Management Engagement) tool. 3. Facilitating patient access to support. | Face to face: individual | General practice | NP | NP | Yes: fidelity checks took place after training. | There were varying levels of implementation in routine practice: information guidebooks were readily used (88% of clinicians reporting use, 51% “regularly”) whereas the PRISMS tool was least used (42% reporting no use). | No |
| McKee et al., 2011 | No | Patients received home-based self-management support focused on goal setting for behaviour change and targeted health/risk communications related to improving the “ABCs” (A1c, BP, and cholesterol). They also received enhanced care navigation via a tele-monitoring system, whereby home readings of blood glucose and blood pressure were sent to their primary care provider. | Home health nurse (HHNs) took part in workshops to enhance skills in promoting self-management, covering selected health behaviour counselling techniques (motivational interviewing) to control primarily blood pressure, as well as glucose and lipids. The program manager was a nurse certified diabetes educator. Also, clinicians were educated to meet the clinical guidelines for HbA1c and blood pressure. | Face to Face and telemonitoring equipment- individual | Patient homes | NP | Intervention delivered in English or Spanish. | No | 25 out of 31 patients received the entire protocol. There were 10.4 home nursing visits over an average of 75.2 days (SD = 35.6), or 10.7 weeks. | Participants received modest incentives for completing the research interviews, but not for home visits or telemonitoring (amount not provided) |
| Mercer et al., 2016 | No | Patients received a whole-systems care intervention which included longer consultations and additional patient self-management support packs containing materials such as a cognitive behavioural therapy-derived self-help booklet. | General practitioners who received training over 3 half days on how to use the longer CARE Plus structured consultations to carry out a holistic assessment. This included identification of patient concerns and priorities, a focus on self-management, and agreeing on a care plan. They also 20–30 minutes of mindfulness-based stress reduction. | Face to face: individual | General practitioner | 12 months period. | NP | Yes: intervention fidelity was estimated from the details recorded on the CARE Plus care plan by practitioners and the patient-reported questionnaire data. | Intervention patients received longer initial consultations, with a mean length of 36.9 minutes (SD 9.8), according to the care plan, and a mean of 34.1 minutes (12.7) according to the patient report. Mean time per patient in the CARE Plus consultation was 69.2 minutes (SD 30.18). Practitioners reported giving the self-management pack to 97 % of patients | £5 gift voucher on completion of each questionnaire. |
| Riley et al., 2001 | Social cognitive theory, Social-ecological theory, and Self-efficacy theory | Participants met with a health educator to set self-management goals, identify barriers, and supports to self-management and problem-solve. Health educators provided feedback on the CIRS score and helped to identify social-environmental resources relevant to that goal. After setting up the self-management plan, participants received follow up support. | Health educator | Face to face, telephone, and newsletters: individual | Participants homes and remote | One visit at the start of the intervention, followed by the first newsletter immediately after. One, follow-up, 5-minute, phone call took place 1 week after the visit. The second tailored newsletter was sent 5-weeks after the visit. | NP | Yes: using RE-AIM Framework | All intervention components were implemented 100% as intended, with the exception that one participant did not receive the follow-up phone call. | No |
| Swerissen et al. 2006 | Social learning theory | Participants received the Chronic Disease Self-Management Program developed at Stanford university. | Peer-leaders who received 20 hours of training from two master trainers prior to leading the program. | Face to face: group | Community settings such as, senior citizens clubs, churches, and community health centres. | 6 weekly sessions, each session being 2.5 hours long. | The peer-leaders were bilingual. All programs were delivered in participants’ first language (Chinese, Italian, Greek or Vietnamese). | No | NP | No |
| Willard-grace-2015 | No | Patients receive health coaching during a three stage medical visit which consisted of a pre-visit, a collaborative medical check-up with a clinician and a post visit session. | Medical assistants retrained as health coaches. They received 40 hours of training in collaborative communication, disease-specific knowedge, medication adherence, developing actions plans and knowledge of community and clinic resources. | Face to face and telephone: individual. | Community clinic for face to face visits and remotely from patients' homes. | The clinic visits were at least once every 3 months and telephone follow-ups were at least monthly over a 12 month period. | The health coaches were bilingual in English and Spanish, self-identified as Latina, and had not received a four-year college education. | Number of health coach interactions per patient. | mean= 12.4, (SD=7.4) | Participants received $10 for taking part in a 45 minute pre-randomisation interview. |

*Supplementary table 5: Self-management components*

| ***Author, year*** | ***Self-management components based on Barlow et al., 2002*** | | | | | | | ***Other details including medical treatment*** | ***Control description*** | ***References for additional material*** |
| --- | --- | --- | --- | --- | --- | --- | --- | --- | --- | --- |
|  | ***Information*** | ***Drug management*** | ***Symptom management*** | ***Management of psychological consequences*** | ***Lifestyle changes*** | ***Social support*** | ***Communication*** |  |  |  |
| Anderson et al., 2010 | Yes: During the brief clinical assessment | Yes: Problem solving discussions around medication adherence. | Yes: Glucose monitoring and reviewing home results. | Yes: Stress management | Yes: Diet, exercise, smoking cessation and developing specific goals. | No | No | No | Usual care |  |
|  |  |  |  |  |  |  |  |  |  |  |
|  |  |  |  |  |  |  |  |  |  |  |
| Arora et al., 2013 | Yes: Educational texts and trivia questions on diabetes myths | Yes: Medication reminder texts. | Yes: Texts containing glucose monitoring, blood pressure monitoring, foot care information | No | Yes: Texts containing healthy living challenges, which were specific daily challenges related to diet and food choices such as not drinking juice. | Yes | No | No | Usual care |  |
| Baig et al., 2015 | Yes: Participants were given information about diabetes. | No | No | No | Yes: Participants were taught healthy Mexican recipes and at-home exercises not requiring special equipment. They were informed of church sponsored exercise programs. Also taught a cognitive approach to behavioural problem solving including goal setting, anticipating obstacles, stimulus control and behavioural alternatives. | Yes: Social support gained from the group setting | No | No | Enhanced usual care including, one 90-minute lecture on diabetes self-management. | ^84^ |
| Berry et al., 2016 | Yes: Teaching patients to understand the complications of diabetes | No | Yes: teaching patients to understand blood pressure, cholesterol, and blood glucose–monitoring goals before meals, after meals, and long-term (A1C). Also, understanding the importance of proper foot self-examination and foot care | No | Yes: teaching patients to understand the importance of nutrition and exercise goals. | No | No | Patients could also have their medications reviewed and an individual medical examination during the group sessions. | Individual appointment with a nurse or physician once every 3 months for 15 months. |  |
| Chamany et al., 2015 | No | Yes: Calls addressed problem solving and self-efficacy regarding medication adherence and patients were sent supportive retention aids such as a seven-day pill box, which they were encouraged to use over the phone. | No | Yes: Calls could address stress management as an optional topic, depending on the participants' preferences. | Yes: Calls addressed self-efficacy and problem solving regarding physical activity and exercise. Patient were also sent items such as pedometers and were encouraged to use them over the phone | No | No | No | Control patients were given the same print materials and retention items. | ^85^ |
| Clancy et al., 2007 | Yes: visits discussed the complications of diabetes | No | Yes: visits discussed foot care | Yes: visits discussed the emotional aspect of diabetes | Yes: visits discussed healthy eating strategies, nutrition, and exercise. | No | No | Vaccinations, foot exams, medication adjustments. Laboratory orders and referral for retinal exams also took place during the group visits. | Usual care in the tradition patient-physician dyad. |  |
| Davis et al., 2010 | No | Yes: The video conferencing included a session titled "Know Your Medicines" | Yes: Session titled "Foot Care Basics & Know Your Numbers". Also, participants were given logs to self-monitor their blood glucose. | Yes - Sessions titled "Stick With It: Positive Thinking" and "Stress Management | Yes: The program includes goal setting and sessions titled "Welcome and Health Eating", "Keeping well and healthy", "Be a Food Detective", "Healthy Eating Out" and "Shop Smart". The last session was in-person at a local grocery shop. | Yes: session titled "Community Resources, Social Support" | No | Optional retinal examinations. | Control patients were given one 20 minute diabetes education session, using ADA materials, conducted individually. They were also given access to usual care and community resources, including care managers for goal setting and education. |  |
| Fitzpatrick et al., 2022 | Yes: modules outlined information on diabetes. | Yes: modules covered medication adherence | Yes: modules outlined clinical targets for blood glucose, HDLs, LDLs and blood pressure. | No | Yes: modules covered lifestyle changes such as healthy eating and physical activity. | No | No | No | Control patients were emailed diabetes materials monthly and received navigation support for medical and social resources. The received 2 follow-ups over the 6 month period. | ^86^ |
| Fortmann al., 2017 | NP | Yes: example text, "Tick, tock. Take your medication at the same time every day!”" | Yes: Participants received a blood glucose meter, test strips and instructions on use. Example texts for monitoring prompts include "Time to check your blood sugar. Please text back your results." ; 1 value >250 or <70 mg/dL or 3 values between 181 and 250 mg/dL prompted a study coordinator to call the participant to assess possible reasons for hyperglycaemia/hypoglycaemia. | NP | Yes: example text, "Use small plates! Portions will look larger, and you may feel more satisfied after eating." | Yes: example text, "Get the support you need family, friends and support groups can help you to succeed.” | NP | no | Control patients also received the 15-min diabetes educational video developed by Scripps, a blood glucose meter and testing strips, with instructions. Afterwards they continued with usual care which included visits with a primary care physician, certified diabetes educator, and group DSME, although the use of the services was dependent on physician and patient initiative. | OTHER PROJECT DULCE INTERVENTIONS INCLUDED IN THIS STUDY. |
| Frosch et al., 2011 | NP* | NP | NP | NP | Yes: During the coaching intervention the nurse educator collaborated with participants to identify desired and attainable behavioral goals that could have a positive impact on their diabetes management. Together a behavioural plan was developed and monitored. | NP | NP | NP | Control patients received a 20-page brochure entitled “4 Steps to Control Your Diabetes for Life,” that was developed by the National Diabetes Education Program of the National Institutes of Health. |  |
| Gary et al., 2009 | Yes: CHWs gave patients feedback on their blood pressure and blood glucose results during home visits and provided health education that would be followed up in the IAPs. | Yes: IAPs could address medication adherence such as problems understanding the prescribed regime or obtaining the drugs. Follow up actions by the CHW involved home visits to organise and monitor pill-taking behaviour. | Yes: There were IAPs on foot care and home visits involved blood glucose monitoring. | No | Yes: There were nutrition and physical activity IAPs. Also, during home visits CHWs could review patients’ fridges/ cabinets and take them on grocery field trips to educate them on health food choices. CHWs could also facilitate group walking exercises. | Yes: During home visits CHWs could involve family members and teach supportive behaviours such as, how to perform glucose monitoring for a patient with poor eyesight. | No | NCMs oversaw any aspect of the intervention requiring nursing expertise such as participating in the upward titration of insulin dose and prompting the physician regarding sub-optimal care patterns. | The minimal intervention involved telephone calls every 6 months to remind patients of preventive screenings and a written summary of healthcare utilisation was sent to their primary care provider. They also received diabetes-related educational material in the mail. | ^87^ |
| Greenhalgh et al., 2011 | Yes: exchanging diabetes knowedge during sessions. | Yes: themes such as "medication" | Yes: stories around foot care and symptom management | Yes: discussions around the emotional impact of diagnosis and the affect it has on identity. | Yes: discussions/ stories around diet and exercise. | Yes: themes such as "feeding the family" and discussions around "mobilising a care network" . The group setting also provided social opportunities. | Yes: themes such as "dealing with doctors" | No | Participants received a nurse-led group diabetes education sessions held in the hospital or community settings. | ^88^ |
| Hill-briggs et al., 2011 | No | Yes: the education session covered the self-management behaviours of taking medications. | Yes: the education session covered control of blood sugar, blood pressure, and cholesterol and self-monitoring. | Yes: one of the problems solving sessions covers how to take control of stress and emotion through adaptive thinking techniques. | Yes: the education session covered eating healthy, and getting physical activity | No | No | No | Control patients received a condensed version of the intervention. 1 education session and 1 problem solving session. |  |
| Lynch et al., 2014 | No | No | Yes: LIFE classes covered blood glucose self-monitoring. | No | Yes: LIFE classes focused on helping participants adapt a low-sodium, moderate-carbohydrate DASH (Dietary Approaches to Stop Hypertension) diet. Participants received a nutrition education workbook and a daily food log. They also received a pedometer and were told to set a step goal. LIFE classes included a peer supporter led moderate aerobic activity along with music. | Yes: LIFE classes and telephone calls also provided emotional and social support. | No | No | Two 3-hour self-management training classes taught by an African American community health worker. One class focused on diabetes self-management and the other on nutrition. |  |
| lynch et al., 2018 | No | Yes: materials covered medication adherence. | Yes: participants were given glucometers and glucose test strips and a daily log to monitor results. The sessions covered information on hyperglycaemia and hypoglycaemia. | Yes: materials covered healthy coping. | Yes: the core of the sessions DSME curriculum was focused on healthy eating such as : carbohydrate counting, reading food labels, a grocery shop tour and eating more vegetables and wholegrains etc. They used a modified version of the plate method. They were also given food logs. Participants were given resistance bands and a 10-minute resistance band workout was included in every group session. They were also given an accelerometer and were encouraged to track steps and meet the 10,000 steps per day goal. Peer supporters provided encouragement through telephone follow-ups. | Yes: Peer supporters provided social support. The group sessions had a dedicated "listening session" where participants could share their struggles as well as their communal wisdom and expertise. | No | No | 2 DSME sessions, delivered in the clinic, by a registered dietician in the first 6 months of the study period. Control participants also received glucometers. | ^89^ |
| Nelson et al., 2017 | Yes: two of the mandatory education topics were "what is diabetes?" and "treating diabetes". | Yes: one of the mandatory education topics was "diabetes medications" | Yes: two of the mandatory education topics were "signs and symptoms of low and high blood sugar" and "blood glucose monitoring" | NP | Yes: two of the mandatory topics were "food and diabetes" and "diabetes and physical activity". Optional topics/ activities included attending a community kitchen or a CHW led grocery shopping tour to demonstrate how to make economical yet healthy food choices. | Yes: CHWs mobilised social support for participants by encouraging family and other members of participants’ support networks to help participants by encouraging lifestyle changes and medication adherence, attending clinic visits, and providing emotional support. | NP | CHWs facilitated coordination with primary care and case managers and encouraged participants to visit their provider for regular check-ups. | Usual care, including, medical care, community resources and one CHW visit after 12 months. | ^90^ |
| Pérez-Escamilla, et al. 2015 | Yes: Visit 2 was an "intro to diabetes". 3 subsequent visits addressed complications of diabetes. | Yes: visits discussed medication adherence, especially visit 7 "medications" | Yes: visits discussed diabetes complications and home glucose monitoring. They were also given a glucometer and glucose test strips. | Yes: visit 11 focused on mental health | Yes: visit 10 focused on physical activity. Visits 3, 4, 5, 6, 9 and 15 focused on nutrition and related topics such as portion size and food labels. Visit 9 involved an onsite grocery shopping activity. | Yes: Family members, if present, were allowed to sit in during the home visits | No | CHWs had weekly meetings with the health management coordination team at the clinic, to update them on self-management barriers faced by the participants. The medical providers were able to provide feedback and suggestions | Usual care- physicians were expected to check HbA1c levels every 3 months and to conduct yearly foot, urine, and eye examinations. Control participants received glucometers and glucose test strips with instructions on use. They were able to purchase medications at a discounted cost. Also, referrals to the clinic dietician were provided when needed. |  |
| Philis-Tsimikas et al., 2011 | Yes: the curriculum covered diabetes and its complications. | Yes: The curriculum medication adherence and cultural myths/ beliefs interfering with management such as, fear of using insulin and nopales, such as Mexican prickly pear cactus, as cures. | Yes: Participants were given glucometers and test strips. The curriculum covered blood glucose monitoring and cultural myths/ beliefs interfering with monitoring such as relying on urine. | Yes: the curriculum covered emotional experiences. | Yes: the curriculum covered diet and exercise. | Yes: during classes participants could share their experience and receive advice and social support from each other. | No | PE's had access to lab results and if they noticed that a participant was not meeting treatment guidelines, they encouraged them to seek further help from their primary care provider but did not offer medical advice themselves. | Usual care and free glucometer and test strips. |  |
| Protheroe et al., 2016 | Yes: discussed perceptions of risk from diabetes | NP | NP | NP | Yes: discussed advantages and disadvantages of behaviour change | NP | NP | LHTs advised participants about essential health care tests and checks they should receive regularly as advised by Diabetes UK. | Usual medical care, including a review by their family doctor at least once every 12 months. |  |
| Pyatak et al., 2018 | Yes: Modules 2 and 7 deal with what diabetes is, its treatment and long term complications. | Yes: Module 4 "Activity and health" (flexible based on participants' needs) | Yes: Module 4 "Activity and health" (flexible based on participants' needs) | Yes: Module 6, "Emotions and Wellbeing" deals with: emotions such anxiety, depression, anger, guilt, denial, fear; coping with diabetes burnout and self-destructive behaviours; promoting well-being and developing positive coping strategies. | Yes: Module 4 "Activity and health" (flexible based on participants' needs) deals with establishing and maintaining health-promoting habits and routines such as carbohydrate counting skills. | Yes: module 5 "Social Support" deals with: managing diabetes in social situations, dealing with “diabetes police”, family-household life, peer relationships, and intimate relationships. In some sessions OTs engaged with family members to resolve social support problems identified by the participant. | Yes: Module 3 "Access and Advocacy" deals with accessing health care and self-advocacy and communication in health care and community settings. | No | Attention control- included an initial home visit and 11 follow-up phone calls, delivered biweekly. Phone calls followed a script and a staff member delivered a standardised set of educational materials published by the National Diabetes Education Program and MyPlate.gov. | ^91,92^ |
| Rosal et al., 2005 | Yes: session topics included enhancing understanding of basic facts about the disease | Yes: session topics included the role of medications and adherence. | Yes: session topics included adherence to daily blood glucose self-monitoring and understanding of values. | Yes: session topics included stress management. | Yes: session topics included dietary guideline education, menu planning and a supermarket tour. Topics also included physical activity with an emphasis on walking. | Yes: session topics included family support. Family members could attend sessions as a way to elicit home-based support/approval for the participant. | No | No | Control participants were given a simple booklet describing the importance of lifestyle factors in diabetes management and providing recommendations for diet, PA, and blood glucose monitoring |  |
| Rosal et al., 2011 | Yes: session 2 covers "what is diabetes", other session also touched on diabetes complications. | Yes: sessions cover medication adherence. | Yes: participants were given glucometers and a tracking log. Sessions cover self-monitoring of blood glucose and management of hypoglycaemia. | Yes: sessions covered stress management | Yes: Participants were given a step counter and were encouraged to increase their daily steps. Sessions covered physical activity and various aspects of diet such as reading food labels and portion control. Reinforcement activities included cooking healthy meals during sessions, food bingo and a supermarket tour. | Yes: family and friends could attend the group sessions. | Yes: sessions covered topics such as communicating and keeping in touch with health care providers and what to ask them. | No | usual care | ^93^ |
| Ruggiero et al., 2014 | Yes: through the education materials provided. | Yes: coaching content included medication adherence. | Yes: coaching content included blood glucose self-monitoring and foot care. | Yes: coaching content included healthy coping. | Yes: coaching content included healthy eating, smoking cessation and physical activity | No | No | The MAC also supported the patient in arranging appointments and made referrals. | Usual care, including regular visits with a primary health care provider, referrals for specialty care such as foot and eye exams and basic education delivered by their physician. All participants were given the “Diabetes: You’re in Control” educational booklet at the baseline |  |
| Schillinger et al., 2009 | Yes: health education messages in the form of narratives. | Yes: medication adherence | Yes: self-monitoring of blood glucose and symptom queries | Yes: queries about psychosocial issues (e.g. coping, depressive symptoms, etc.) | Yes: queries on diet and physical activity | No | No | Care manager also facilitated referrals for preventive services (e.g., ophthalmologist, etc.). | Usual care | ^94,95^ |
|  | NP | NP | NP | NP | NP | Yes: group visits included social breaks. | NP | During visits patients with unmet medical needs also received brief, individualized care. |  |  |
| Schoenberg et al., 2017 | Yes: class one gives an over of diabetes and its effect on the body. | Yes: class covers two medications taking, to avoid diabetes complications. | Yes: class two cover blood-glucose self-monitoring. Class 5 covers avoiding feet, teeth, eyes, sick days, kidneys, and blood pressure complications. | Yes: Class fours covers stress management. | Yes: class three covers health eating and class four covers physical activity. | Yes: program covers working with family (class number not specified) | Yes: program covers working with health care providers (class number not specified) | Regarding medical appointments, CHW assisted in rescheduling, arranging transportation, finding dependent care options and motivating on follow through. | Usual care |  |
| Seligman et al., 2018 | Yes: Education materials were given with the food packages. Class topics included a disease overview. | Yes: class topics included a diabetes medications overview, with medical professionals such as a registered nurse or physician as guest speakers. | Yes: class topics included blood sugar monitoring. | Yes: class topics included stress management and depression and healthy coping. Social workers and therapists were guest speakers for these topics. | Yes: Many classes covered aspects of health eating such as carbohydrate counting and reading food labels. Food packages contained diabetes-appropriate food and were accompanied with written healthy recipes. Classes also covered physical activity including exercise instructors as guest speakers. | Yes: the class curriculum involved prompts to ask about questions family members had. | No | Participants also received onsite HbA1c testing at months 3 and 6 and referrals to a primary care provider if they did not already have one. | Wait list control |  |
| Shea et al., 20006 | Yes: via the HTU patients had access to web-based diabetes educational materials | No- not explicitly | Yes: patients could upload their blood pressure and blood glucose measurements on to the HTU, where it could then be reviewed by their case manager. Patients also set HbA1c, cholesterol and blood pressure goals with their case manager during tele-health visits. | No | Yes: nurse case managers supervised patients in setting behavioural goals such as smaller food portions. At each visit, the goal from the previous was reviewed and relevant praise and/or problem solving to barriers were discussed. | Yes: patients and nurse case managers discussed strategies to overcome social barriers such as asking their partner to also cut out unhealthy foods such as "ice cream". | No | no | Usual care by their primary care provider. | ^96,97^ |
| Sixta and Ostwald, 2008 | Yes: patients were taught about the disease and related complications. | No | Yes: patients were taught about blood glucose management. | Yes: Patients were taught about disease "related emotions". | Yes: Patients were taught about healthy behaviours such as the effect of exercise and nutrition. Promotores assisted patients in setting/ revising behaviour goals and assisted in follow-up and problem solving. | No | Possibly- Patients were taught about "multidisciplinary team management". | no | Wait list and usual care. |  |
| Skelly et al., 2009 | Yes: Patients were taught about disease symptoms and how they relate to diabetes. | Yes: Patients were taught about insulin/ oral medication. | Yes: patients were taught symptom management strategies and were given materials on the prevention of symptoms. Patients chose which strategies they wanted to use. Self-care practices taught include home glucose monitoring, foot care and checking urine for ketones if blood sugar was >240. | Yes: Patients were taught several psychological strategies such as positive self-talk, positive coping strategies and stress reduction–abdominal breathing, visual imagery. | Yes: physical activity and diet were addressed for example nurses went with patients to their kitchen to teach them how to read nutrition labels. Patients were given "homework" and set goals at the end of each session. | Yes: Family members, if present, were invited to sit in during the home visits | Yes: Patients were taught when to contact their healthcare provider, for example, to contact their healthcare provider if their readings were frequently>140 before meals. | No | A weight and diet program consisting of four modules that addressed Weight Maintenance (two modules), Modifying Fat, and Modifying Sodium in the diet. The modules did not address symptoms directly. | ^98^ |
| Spencer et al., 2018 | Yes: Participants were taught information about diabetes. | No | No | Yes: When patients set goals and identified problems, they were able to discuss the emotional impact of that problem with the CHWs. The curriculum also taught stress-lowering activities. | Yes: The curriculum involved culturally appropriate diet and physical activity advice, including exercise videos. CHWs also help participants set goals using the 5-step goal setting process ad developing and executing an action plan for that goal. | Yes: The curriculum emphasises that healthy eating is beneficial for the whole family. A group sessions provided social support and role-playing support exercises to improve social support and communication with family members about diabetes self-management | Yes: CHWs helped participants improve communication skills with their providers and facilitated necessary referrals to other services. CHWs accompanied participants to one clinic visit with their primary health care provider. | No | Enhanced usual care, including a 2-h class conducted by a research assistant covering how to interpret their clinical and anthropometric results. | ^99^ |
|  | Yes: PLs addressed questions about diabetes and its care | No | No | Yes: PLs discussed psychosocial concerns with participants. | Yes: Using the same 5-step goal process as the CHWs. Group sessions were an opportunity to discuss challenges and problem solve. | Yes: PLs helped participants take inventory of support sources | Yes: Pls helped participants in developing strategies to navigate the health care system | No | After the initial 6-months of the main intervention, participants randomized to CHW worker only group received monthly telephone calls from a CHW who had led their DSME group to check in and assess their progress. |  |
| Talavera et al., 2021 | Yes: During medical visits the medical provider reviewed patients lab results with them and their medical history. During the DSME classes, groups discussed diabetes pathophysiology in relation to cultural beliefs. | Yes: Medical providers and patients collaboratively discussed barriers to medication adherence during the medical visit. Medication adherence was also discussed during DSME classes | Yes: Medical providers and patients collaboratively discussed home glucose self-monitoring during the medical visit. | Yes: during the behaviour visit patients collaboratively assessed emotional factors affecting diabetes self-management. The behaviour providers also provided psychoeducation. DSME classes involved discussions on psychosocial well-being (prevention and coping with depression, anxiety, diabetes distress, stress management and problem solving). | Yes: During the behaviour visits, patients created SMART goals and personal action plans. During DSME classes, groups discussed nutrition in the context of the traditional Latin diet and how to incorporate physical activity into everyday life. | Yes: during the behaviour visit patents discussed family barriers. Also, patients were refereed to social work/ family services when needed. The DSME curriculum emphasised involving family in self-management and lifestyle activities. | Yes: indirectly | Yes: Medical visits also involved the development of a treatment plan. Care-coordination involved referrals to other health departments and community resources when needed. | Control patients received primary care provider led usual care, with referrals to health education and behavioral health as needed. |  |
| 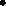 Thom et al., 2013 | Yes: Peer coaches and patients discussed current and target clinical values for HbA1c. | Yes: Peer coaches facilitated medication understanding and adherence. | Yes: Peer coaches discussed self-management skills such as using a glucometer and appropriate strategies for hypoglycaemia | Yes: Peer coaches provided social and emotional support and helped patients with stress management. | Yes: Peer coaches assisted with lifestyle changes such as healthy eating and physical activity. | Yes: Peer coaches provided social support and shared stories about their own lives and families. | Yes: Peer coaches helped patient to navigate the clinic and could accompany the patient during a clinic visit. | Yes: Peer coaches provided information on community resources. | Usual care, including referrals to a nutritionist and diabetes educator if needed. | ^100^ |
| Wang et al., 2018 | No | Yes: covered in the usual care diabetes education. | Yes: the usual care diabetes education covered risk and management of hyperglycaemic and hypoglycaemic situation and blood glucose self-monitoring. Participants also received a Bluetooth enabled glucometer linked to the Diabetes Connect app to track blood glucose. | Yes: the lifestyle intervention sessions cover stress management and balancing thoughts. | Yes: the lifestyle intervention sessions cover various aspects of exercise and healthy eating and includes a grocery shopping tour. Participants were provided with the LoseIt! App, a pedometer, a food scale and a weight scale to track calories, physical activity and weight. | No | No | No | Control group received usual care and diabetes education from their primary care physicians and diabetes educators |  |
|  | No | Yes: covered in the usual care diabetes education. | Yes: the usual care diabetes education covered risk and management of hyperglycaemic and hypoglycaemic situation and blood glucose self-monitoring. Participants also received a regular glucometer to track blood glucose in a paper diary. | Yes: the lifestyle intervention sessions cover stress management and balancing thoughts. | Yes: the lifestyle intervention sessions cover various aspects of exercise and healthy eating and includes a grocery shopping tour. Participants were provided with the CalorieKing paper diary, a pedometer, a food scale, and a weight scale to track calories, physical activity, and weight. | No | No | No |  |  |
| Wayne et al., 2015 | No- not explicitly | Yes: medication adherence was a goal emphasised by the health coaches. | Yes: Participants logged and monitored their blood glucose levels via the app. | Yes: Coaches emphasised stress management as goal for participants. Participants could also log and track their mood on the app. | Yes: Coaches guided participants in setting goals and making plans regarding diet (reducing carbohydrate intake) and increasing exercise frequency. Participants could also access free group exercise classes available at the local community health centre. Participants could log food intake and exercise frequency onto the app for the coaches to monitor and provide guidance when they diverged from their goals. | No | Yes: A goal emphasised by health coaches was participant communication with primary care physicians and, generally, within the health system. | no | Control participants received in-person health coaching only, with no additional mobile support. |  |
| Whittemore-2020 | Yes: session 1 focused on understanding diabetes | Yes: session 1 highlighted the need to take medication and session 5 focused on going diabetes medications. | Yes: Participants were given glucometers, test strips and lancets and were taught the need to self-monitor blood glucose and how this relates to carbohydrate intake. | Yes: 4 of the sessions included stress management activities | Yes: Throughout the sessions and texts/ pictures participants were taught strategies to improve their diet such as how to read nutrition labels, menu planning with limited resources, food portion measurement and the "plate method". Also, the benefits and precautions of physical activity were highlighted, and goals were encouraged through texts. | Yes: family could be invited to sessions. | Yes: session 5 covered how to talk to health care professionals. | No |  | ^100^ |
| Aikens et al., 2022 | Yes: pre-recorded messages contained information on depression symptoms | Yes: pre-recorded messages highlighted the importance of adhering to their anti-depressant regime and advice on how to do so and get refills. | Yes: at the beginning of each pre-recorded call, patients answered PHQ-9 items to track their depression symptom severity, which then tailored the advice they were given. | Yes | Yes: pre-recorded messages covered lifestyle advice such as physical activity and sleep. | Yes: Patients had an "in-home" supporter and CP who lived outside the home. They provided social support. At the end of each IVR call, CPs were sent a structured report along with advice on how to support the patient with their depression. | Yes: pre-recorded messages included advice on when and how to reach out to their physician. | If patients reported suicidal feelings, the system alerted their primary care team. | Control patients received enhanced usual care. While both they and their nominated CP received printed materials on depression self-management, they did not receive additional self-management support via the IVR | ^101^ |
| Apter et al., 2019 | Yes: CHWs taught patients how to search for health information online and access educational materials on the portal. | Yes: CHWs explained the difference between controller and rescue medications, and proper inhaler use. | Yes: CHWs drafted individualised asthma management plans with each patient and taught patients how to mitigate asthma triggers. | No | Yes: signposting to relevant community resources such as smoking cessation and housing programs. | Yes: CHWs also established relationships with patients' families. | Yes: CHWs taught patients how to chat with doctors via the portal, especially regarding exposure to key allergens and booking appointments. | CHWs were involved in care navigation and referrals. | Portal training only + usual care | ^102^ |
|  |  |  |  |  |  |  |  |  |  |  |
|  |  |  |  |  |  |  |  |  |  |  |
|  |  |  |  |  |  |  |  |  |  |  |
| Krieger et al., 2015 | Yes: visits covered asthma basics including asthma pathophysiology, when to seek emergency care and important vaccines. | Yes: visits covered medication adherence including providing participants with a medication box; problem solving concerns about side effects, cost, access and getting to pharmacy; and CHWs assessing participants' understanding of when to use controller medications. | Yes: Visits covered symptom management including: peak flow monitoring- participants were given a peak flow monitor and diary and taught the correct technique; getting help during an asthma attack- CHWs demonstrated “Belly Breathing” and other relaxation techniques. | Yes: visits covered stress management. | Yes: visits covered environmental control of the home and a cleaning checklist. Participants were given a vacuum, vacuum bag and cleaning kit. | Yes: CHWs could engage family support during visits. | Yes: Visits covered working with the healthcare system including communication strategies, pointers and roleplay. If needed, CHWs could accompany participants to a medical appointment to act as a “cultural translator.” | CHWS made referrals to community resources for childcare, food, employment, and citizenship assistance. CHWs also faxed visit details to the clinics for feedback. | Usual care, information on community resources for asthma self-management and educational pamphlets. At the end of the study, participants in the control group received a home visit by a CHW and the intervention group resources. |  |
| Martin et al., 2009 | Yes: group session 1 covered asthma anatomy and physiology and understanding physiologic reactions to stressors. Home visit 3 covered asthma triggers. | Yes: home visit 1 covered controller medications, spacers, inhalers | Yes: home visit 2 covered symptom recognition and management | Yes: group session 2 covered sociocultural definitions of stress; effects of stress on asthma management and an action plan to improvement ability to manage stress. | Yes: groups session 3 and 4 and home visit 5 covered benefits of physical activity an action plan for physical activity. Home visit 4 covered smoking cessation and tobacco smoke avoidance | Yes: group session 4 covered discussions on current positive social support. All home visits also covered social support. | Yes: home visit 2 covered communicating with providers. CHWs encouraged proactive communications between patients and their health care providers. | no | 2 mailings covering the asthma education information presented at the group sessions for the intervention and a US$30 cheque. |  |
| Young et al., 2012 | No | Yes: Pharmacists followed the communication guide to identity and address patient barriers to medication adherence. Pharmacists also used a series of questions to assess whether patients required additional education regarding inhaler technique. | No | No | No | No | No | Pharmacists review their electronic health records. If they identified severe asthma related problems, they referred patients to their primary healthcare provider. | Usual care, including mail receipt of a prescription refill with written instructions on medication use. |  |
| Evans-Hudnall et al., 2014 | Yes: Patients were given a detailed workbook on the signs and symptoms of stroke and risk factors for primary and secondary stroke. | no | Yes: Patients were given a detailed workbook on the signs and symptoms of stroke | Yes: Patients were taught cognitive reframing techniques to minimize negative thoughts concerning their ability to change lifestyle habits. Also, they were taught deep breathing and guided imagery skills to help identify and decrease their stress levels | Yes: Patients were given a dietary and exercise tracking form. Patients were asked to identify lifestyle habits that increased their risk for secondary stroke and how to assess change in these habits, including potential barriers and problem solving. Patients were taught stimulus control- removing environmental factors associated with unhealthy habits. Diet and exercise advice was tailored to each patient. | Yes: Patients were encouraged to engage friends and family members as a source of support to achieve their goals. They were also encouraged to set goals that focused on changing family lifestyle behaviours rather than individual change. | No | The health educator facilitated phone calls to aid the patient in getting access to community resources. | Usual care |  |
| Kronish et al., 2014 | Yes: the workshop covered the biology of stroke and stroke treatments. | Yes: the workshop stressed the importance of adherence to preventive medications to reduce stroke recurrence and provided suggestions for optimizing medication adherence. | Yes: the workshop covered key symptom management related to blood pressure and cholesterol. | No | No | Yes: Participants could bring a family member, friend, or home attendant to the workshops. | Yes: the workshop covered Working with your health care team, including communication. | Participants were also given a list of local health providers, including those that accepted patients without health insurance. | Usual care and wait list. Control participants received the workshop after a 1-year waiting period. | ^103^ |
| Tiliakos et al., 2013 | Yes: Class content included an overview of arthritis pathophysiology. | Yes: Class content included an overview of arthritis medications. | Yes: Class content covered appropriate use of injured joints. | Yes: Class content involved individualised relaxation programs. | Yes: Class content involved the development of individualised exercise programs. | No | Yes: Class content covered aspects of patient-physician communication. | No | Usual care | ^104^ |
| Eakin et al., 2007 | no | no | no | No | Yes: Patients set a self-management goal related to physical activity or healthy eating, and identified one or two types of social environmental resources they could use to help them reach their goal. Patients received a goal sheet that summarised their action plan. Phone calls addressed problem solving. Also, tailored newsletters reinforced these goals. | Yes: Family and friends were included as potential social-environmental resources. | no | NA | Control patients were mailed a local area community resources guide and three newsletters on basic financial management. |  |
| Kangovi et al., 2017 | Yes: If participants wanted further disease education CHWs navigated them to the appropriate clinician. | Yes: the tailored action plans could include strategies for medication adherence. | Yes: the tailored action plans could include strategies for symptom management such as blood glucose and blood pressure monitoring. | Yes: the support groups discussed psychosocial stressors. | Yes: the tailored action plans could include strategies for lifestyle changes such as increased physical activity, healthy eating and quitting smoking. CHW provided support such as food pantry visits with participants. | Yes: The taction plans could involve strategies for involving family in the participants’ goal. Also, the support discussed relationships with friends and family members. | Yes: the action plans could involve discussion pointers to bring up with the participants primary care provider. | CHW also navigated participants towards appropriate community resources and sent progress reports to the participants primary care team. | One time collaborate goal setting, followed by usual care. | ^105,106^ |
| Kennedy et al., 2013 | NP | NP | NP | NP | NP | NP | NP | No | Wait list control | ^107^ |
| McKee et al., 2011 | NP | NP | Yes: Patients were leased Cardiocom telemonitoring equipment to send their daily self-monitored blood pressure and glucose readings. Results were transmitted to the program manager and formatted as weekly reports. The reports were sent to the primary care provider via secure clinical email for review and treatment modification if necessary. | NP | NP | NP | NP | Primary care providers used weekly report to modify treatment plans. | Usual care |  |
| Mercer et al., 2016 | NP | NP | NP | Yes: mindfulness-based stress management CDs | NP | NP | NP | Practitioners were encouraged to link patients with relevant local resources and community services when appropriate. | Usual care | ^108^ |
| Riley et al., 2001 | Yes: CIRS covers whether or not the participant has access to information about theSir condition | Yes: CIRS cover medication taking as a behaviour. | No- not explicitly | No | Yes: CIRS covers behavioural targets such as eating more fruits and vegetables, getting more physical activity and quitting smoking. | Yes: CIRS involves questions arounds family and friend support e.g. "Have your family or friends exercised with you?" | Yes: CIRS covers questions around support/ communication with the participants' healthcare team e.g., "Has your doctor or other health care provider listened carefully to what you had to say about your illness?" | No | Wait list control: received the intervention one month after the intervention group. |  |
| Swerissen et al. 2006 | No- not explicitly | No-not explicitly | Yes: The program manual covers symptom management. | Yes: The program manual covers dealing with the emotions of chronic illness (e.g., anger and depression) and relaxation techniques. | Yes: Program covers exercise and healthy eating. There was weekly action planning and feedback on progress. Also there was modelling of self-management behaviours and problem solving strategies. | Yes: Program covers communication skills with friends and family. | Yes: Program covers communication skills with health care providers. The program emphasizes the critical role of the patient managing their own health in partnership with health professionals. | No | Wait list control- participants received the intervention six months later. |  |
| Willard-grace-2015 | Yes: The health coach assesses the patient’s knowledge about HbA1c, systolic blood pressure (SBP), or low density lipoprotein (LDL). They discuss the patient's most recent results for these measures, their goal for these numbers and how to reach the goal | Yes: During the pre-visit, health coaches go through "medication reconciliation" with the patient, which includes reviewing the medications under prescription, assessing patients knowledge of the purpose of the medications and identifying barriers to adherence. | No- Not mentioned explicitly. | Yes: During the "post visit", health coaches negotiate an "action plan" with the patient which includes strategies to reduce stress. | Yes: The "action plan" also addresses diet, exercise, and other relevant lifestyle factors. The telephone follow-ups address barriers and problems with meetings these goals. | No | Yes: During the medical visit the health coach acts as an advocate for the patient, helping them to remember questions or concerns raised during the pre-visit and praising the patients, relaying to the clinician steps the patient has taken to care for their health. | Health coaches were also responsible for further referrals to specialists and resource navigation. | Usual care, including access to clinic resources that would normally be available such as visits with a clinician, diabetes educator, nutritionist, chronic care nurse and educational classes. | ^109,110^ |

|  | | | | | | **Summary of Findings** | | | |
| --- | --- | --- | --- | --- | --- | --- | --- | --- | --- |
| **Quality Assessment** | | | | | | **Number of participants** | | **Mean change (95% confidence interval)** | **Certainty of evidence** |
| **Number of studies (design)** | **Risk of Bias** | **Inconsistency** | **Indirectness** | **Imprecision** | **Publication Bias** | **Self-management intervention** | **Control** |  |  |
| **Mean change in HbA1c%** | | | | | | | |  |  |
| Thirteen (RCT and pilot studies) | Serious ^a^ | Not serious ^b^ | Not serious ^c^ | Not serious ^d^ | Undetected ^e^ | 1239 | 1255 | -0.29 (-0.48 to -0.10) | Mode rate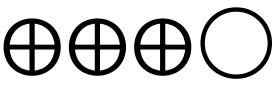 |

*Supplementary table 6: GRADE Assessment*

^a^ 9 out of 13 studies had moderate or high risk of methodological bias. However, this was mainly due to insufficient outcome data.

^b^ I^2^ = 32.46%, which suggests low heterogeneity. Cochrane Q- P=0.16, which provide strong evidence for homogeneity. Majority of confidence intervals overlap. Consistent direction of effect, which favours the intervention groups.

^c^ Similar population across all studies. While HbA1c could be considered a surrogate outcome, it is well established as a marker of improvement in diabetes control.

^d^ 13 studies (>10) included in the meta-analysis. The magnitude of the median sample size (110) is intermediate. The confidence interval of the pooled difference does not cross the line of null effect.

^e^ Funnel plot is mostly symmetrical and the Egger test for funnel plot asymmetry suggest that there is no evidence of small study effects (P-0.2355). This review used a comprehensive search strategy. However, we excluded non-English texts and did not include grey literature.

*Supplementary figure 1: Tabulation of positive and non-positive outcomes. Note n=52 as one 3-arm RCT had separate results for the two intervention arms*




 Supplementary Figure 2 : Contour-enhanced funnel plot with contours set at P>0.1, 0.05< P<0.1 and 0.01<P<0.05. The contour enhanced funnel plot is fairly symmetrical and there are seemingly few missing studies in the dark grey region, representing studies with non-significant results. Similarly, the Egger test suggests that there is funnel plot symmetry and no evidence of small study effects (P=0.2355).
